# Fair human-centric image dataset for ethical AI benchmarking

**DOI:** 10.1038/s41586-025-09716-2

**Published:** 2025-11-05

**Authors:** Alice Xiang, Jerone T. A. Andrews, Rebecca L. Bourke, William Thong, Julienne M. LaChance, Tiffany Georgievski, Apostolos Modas, Aida Rahmattalabbi, Yunhao Ba, Shruti Nagpal, Orestis Papakyriakopoulos, Dora Zhao, Jinru Xue, Victoria Matthews, Linxia Gong, Austin T. Hoag, Mircea Cimpoi, Swami Sankaranarayanan, Wiebke Hutiri, Morgan K. Scheuerman, Albert S. Abedi, Peter Stone, Peter R. Wurman, Hiroaki Kitano, Michael Spranger

**Affiliations:** 1 Sony AI, New York, NY USA; 2 Sony AI, Zurich, Switzerland; 3 Sony AI, Tokyo, Japan; 4 Sony AI, Bengaluru, India

**Keywords:** Ethics, Computer science, Interdisciplinary studies, Databases, Ethics

## Abstract

Computer vision is central to many artificial intelligence (AI) applications, from autonomous vehicles to consumer devices. However, the data behind such technical innovations are often collected with insufficient consideration of ethical concerns^1–3^. This has led to a reliance on datasets that lack diversity, perpetuate biases and are collected without the consent of data rights holders. These datasets compromise the fairness and accuracy of AI models and disenfranchise stakeholders^4–8^. Although awareness of the problems of bias in computer vision technologies, particularly facial recognition, has become widespread^9^, the field lacks publicly available, consensually collected datasets for evaluating bias for most tasks^3,10,11^. In response, we introduce the Fair Human-Centric Image Benchmark (FHIBE, pronounced ‘Feebee’), a publicly available human image dataset implementing best practices for consent, privacy, compensation, safety, diversity and utility. FHIBE can be used responsibly as a fairness evaluation dataset for many human-centric computer vision tasks, including pose estimation, person segmentation, face detection and verification, and visual question answering. By leveraging comprehensive annotations capturing demographic and physical attributes, environmental factors, instrument and pixel-level annotations, FHIBE can identify a wide variety of biases. The annotations also enable more nuanced and granular bias diagnoses, enabling practitioners to better understand sources of bias and mitigate potential downstream harms. FHIBE therefore represents an important step forward towards trustworthy AI, raising the bar for fairness benchmarks and providing a road map for responsible data curation in AI.

## Abstract (web summary)

The Fair Human-Centric Image Benchmark (FHIBE, pronounced ‘Feebee’)—an image dataset that implements best practices for consent, privacy, compensation, safety, diversity and utility—can be used responsibly as a fairness evaluation dataset for many human-centric computer vision applications.

## Main

Image datasets have played a foundational role in the history of AI development, with ImageNet^12^ enabling the rise of deep learning methods in the early 2010s^13^. While AI technologies have made tremendous strides in their capabilities and adoption since then, bias in data and models remains a persistent challenge^2,14^. Inadequate evaluation data can result in fairness and robustness issues, making it challenging to identify potential harms^1,10,15^. These harms include the perpetuation of racist, sexist and physiognomic stereotypes^2,4^, as well as the exclusion or misrepresentation of entire populations^3,5,16^. Such data inadequacies therefore compromise the fairness and accuracy of AI models.

The large-scale scraping of images from the web without consent^2,6,17^ not only exacerbates issues related to data bias, but can also present legal issues, particularly related to privacy^7,18,19^ and intellectual property (IP)^20^. Consequently, prominent datasets have been modified or retracted^8^. Moreover, the lack of fair compensation for data and annotations presents critical concerns about the ethics of supply chains in AI development^21,22^.

Datasets made available by government agencies such as NIST^23^ or using third-party licensed images^24^ often have similar issues with the absence of informed consent and compensation. Many dataset developers mistakenly assume that using images with Creative Commons licences addresses relevant privacy concerns^3^. Only a few consent-based fairness datasets with self-reported labels exist^25–27^. However, these datasets have little geographical diversity. They also lack pixel-level annotations, meaning that they can be used for only a small number of human-centric computer vision tasks^3^.

Evaluating models and mitigating bias are key for ethical AI development. Recent methods such as PASS^28^, FairFaceVar^29^ and MultiFair^30^ aim to reduce demographic leakage or enforce fairness constraints through adversarial training and fairness-aware representations. Previous work has also shown that many face-recognition models and benchmarks encode structural biases, underscoring the need for fairness at every stage of development^31^. Yet, these methods remain constrained by the same dataset limitations that they seek to address, including a lack of consent, demographic self-identification and global representation. Most research in the computer vision fairness literature relies on repurposing non-consensual datasets that lack self-reported demographic information. This lack of self-reported demographic information then leads researchers to guess complex social constructs, such as the race and gender of image subjects, from images alone. These inferences can entrench stereotypes^32,33^, cause psychological harm to data subjects when inaccurate^34,35^ and compromise the validity of downstream tasks^36^.

The dearth of responsibly curated datasets creates an ethical dilemma for practitioners who would like to audit bias in their models. Their options are to use (1) diverse and densely annotated public datasets that carry legal or ethical risks; (2) one of the few publicly available consent-based but highly limited datasets (requiring them to add their own pixel-level annotations); (3) proprietary datasets that do not provide transparency to external parties; (4) datasets that have been quietly retracted due to ethical concerns but continue to circulate in derivative forms^37^; or (5) nothing—simply to not check for bias^7,11,18^.

To address these challenges, we introduce the FHIBE, a publicly available, consensually collected, globally diverse fairness evaluation dataset for a wide range of vision-based tasks, from face verification to visual question answering (VQA). FHIBE comprises 10,318 images of 1,981 unique individuals from 81 countries/areas^38^. Current consent-based fairness datasets^25–27^ lack data from regions with stringent regulations, such as the European Union (EU), making FHIBE, to our knowledge, the first publicly available, human-centric computer vision dataset to include consensually collected images from the EU. FHIBE features the most comprehensive annotations to date of demographic and physical attributes, environmental conditions, camera settings and pixel-level annotations. To assess FHIBE’s capabilities, we used it to evaluate bias in a wide variety of narrow models (designed for specific tasks) and foundation models (general purpose) commonly used in human-centric computer vision. Our analyses spanned eight narrow model tasks (pose estimation, person segmentation, person detection, face detection, face parsing, face verification, face reconstruction and face super-resolution), along with VQA for foundation models. We affirm previously documented biases, and we show that FHIBE can support more granular diagnoses on the factors leading to such biases. We also identify previously undocumented biases, including lower model performance for older individuals and strong stereotypical associations in foundation models based on pronouns and ancestry.

A large number of participants were involved in the data collection, annotation and quality assurance (QA) processes for our project (as described in Supplementary Information C). To collect a dataset as globally diverse as possible, we worked with data vendors to collect data from crowdsourced image subjects. Additional annotations were also collected from crowdsourced and vendor-employed annotators. We provided extensive guidelines to vendors and performed additional steps for QA, privacy preservation, IP protection and consent revocation to further protect the rights of those involved in the data-collection process (Methods). By creating FHIBE, we not only provide researchers with a new evaluation dataset, but we also show the possibilities and limitations of responsible data collection and curation in practice.

## FHIBE

### Overview

FHIBE comprises 10,318 images of 1,981 unique individuals, averaging six images per primary subject. We used a crowdsourcing approach, working with data vendors that operate globally to collect the dataset. We developed comprehensive data-collection guidelines and implemented a rigorous quality assessment protocol, which we discuss in detail in the Methods.

**Fig. 1:**
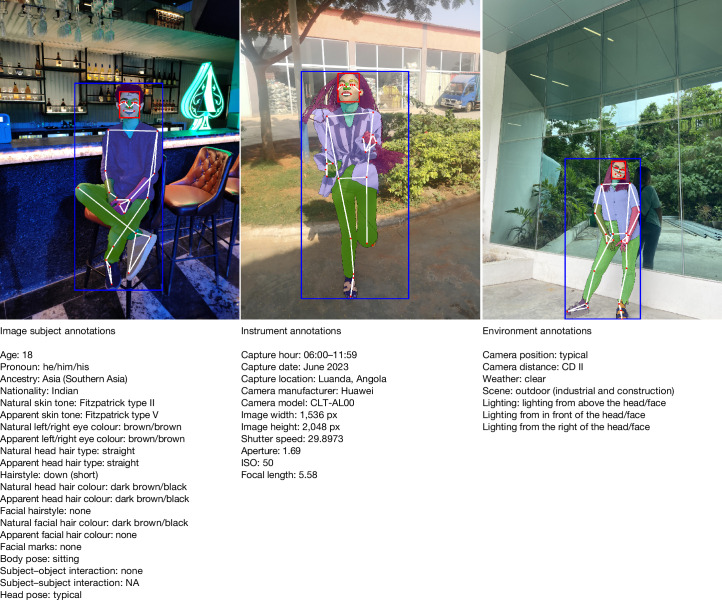
Annotations about the image subjects, instrument and environment are available for all images in FHIBE. For visualization purposes, we display one type of metadata per image in this figure. Each annotation is linked to the annotators who made or checked the annotation. If the annotator disclosed their demographic attributes (age, pronouns, ancestry), that information is also provided. A full list of annotations is provided in Supplementary Information A. NA, not applicable.

The dataset includes 1,711 primary subjects (individuals submitting images of themselves; Supplementary Information C) and 417 secondary subjects (individuals who appear alongside primary subjects, increasing the diversity and complexity of the images). Note that some primary subjects are also secondary subjects in other images. In total, 623 images contain both primary and secondary subjects. Captured between May 2011 and January 2024, the images span 81 countries/areas across 5 regions and 16 subregions^38^. To increase the diversity of the images (location, clothing, appearance, environmental conditions and so on), we permitted participants to submit images that they had previously taken of themselves. The images were taken with 785 distinct camera models from 45 manufacturers, and represent a wide range of real-world conditions, including 16 scene types, 6 lighting conditions, 7 weather scenarios, 3 camera positions and 5 camera distances. Example images with the accompanying subject, instrument and environment metadata are provided in Fig. 1.

FHIBE also features self-reported pose and interaction annotations, with predefined labels categorized into 16 body poses, 2 head poses and 47 distinct interactions—14 with other subjects and 33 with objects. The dataset offers a rich array of appearance characteristics, including 15 hair and 4 facial hair styles, 7 hair types, 13 hair and 12 facial hair colours, 9 eye colours and 11 types of facial marks.

There are also 6 pronoun categories, 56 integer ages (18 to 75 years) grouped into 5 age categories, 20 ancestry subregions within 5 regions and 6 Fitzpatrick skin tones^39^. There are 1,234 intersectional groups defined by age group, pronoun, ancestry subregion and Fitzpatrick skin tone, with the number of images per group ranging from 1 to 1,129, with a median of 9 images.

**Fig. 2:**
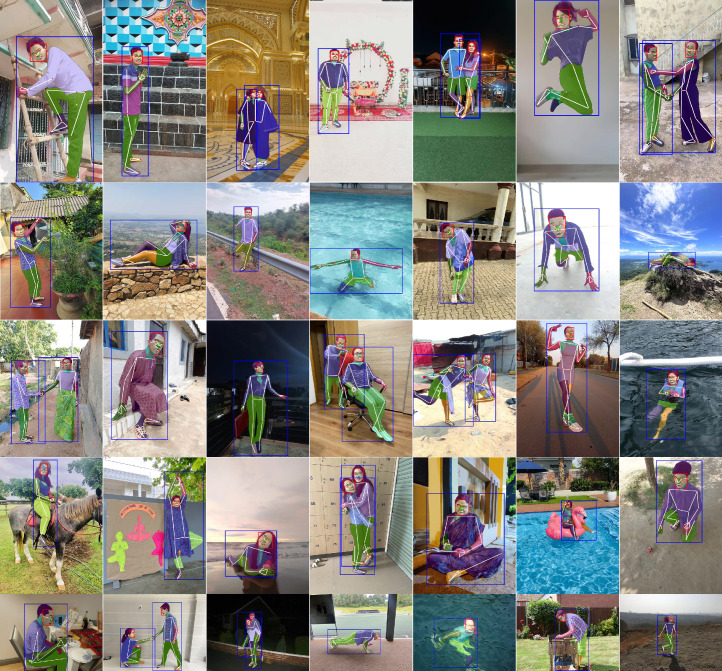
Example FHIBE images annotated with detailed pixel-level annotations, keypoints, segmentation masks and bounding boxes. Pixel-level annotations include keypoint annotations (small red circles) indicating the geometric structure (white lines) of human bodies and faces (for example, right eye inner, left foot index); segmentation masks dividing the human body and face into segments, assigning a label to each pixel (for example, left arm, jewellery); and face and person bounding boxes (red and blue rectangles, respectively).

**Extended Data Fig. 1:**
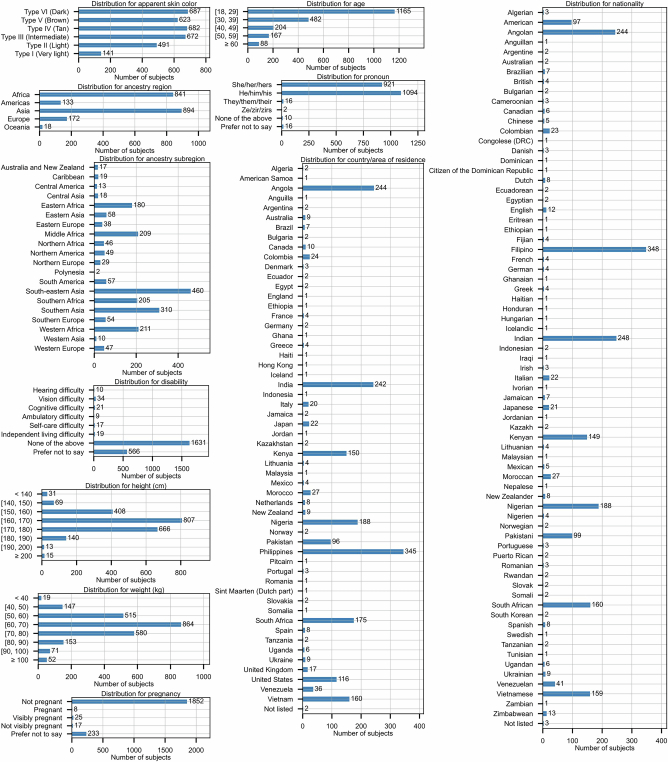
Distribution of subjects associated with key attributes in FHIBE. This figure shows the distribution of subjects corresponding to key attributes in the FHIBE dataset. Some subjects may have multiple annotated labels for specific attributes, resulting in variations in the total sample count across attributes. In compliance with the IRB protocol, certain sensitive attributes are not publicly released, as detailed in Supplementary Information A. For transparency, the aggregated distribution of key sensitive attributes is presented. While a few extreme outliers are observed in the self-reported weight and height values, these do not significantly affect the overall distribution.

**Extended Data Fig. 2:**
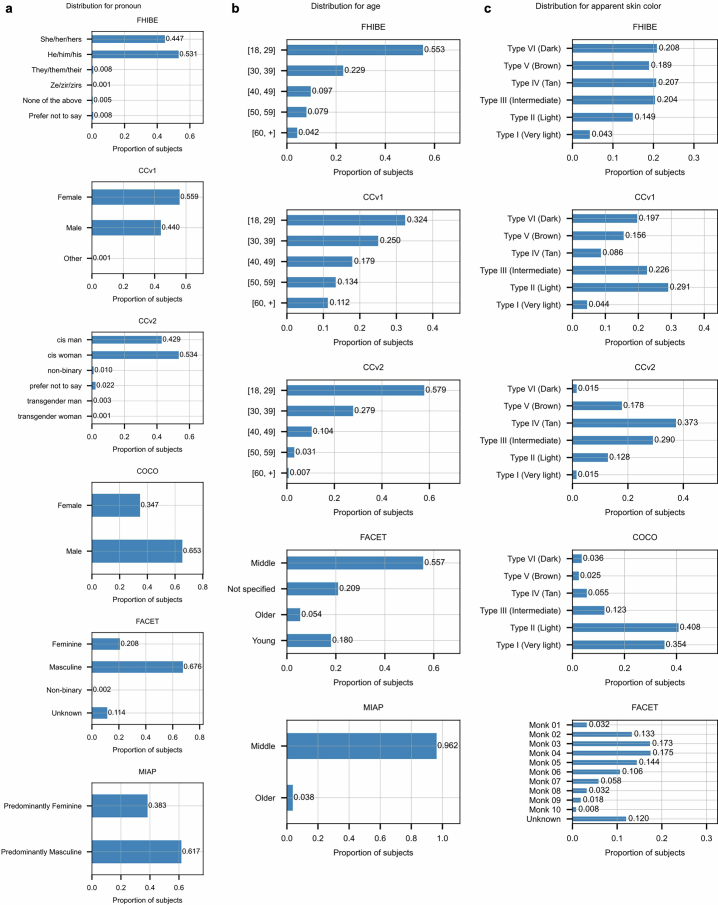
Proportional distribution of subjects for pronoun, age, and apparent skin colour across FHIBE and other datasets. This figure compares the proportional distribution of subjects for (a) pronoun, (b) age, and (c) apparent skin colour attributes in FHIBE and other datasets used in this paper. Original attribute labels are preserved. Datasets lacking a specific attribute are excluded from the corresponding subfigure. Note that some subjects may have multiple annotated labels for specific attributes, resulting in variations in the total sample count across attributes.

FHIBE includes pixel-level annotations for face and person bounding boxes, 33 keypoints and 28 segmentation categories (Fig. 2). Annotator identifiers (an anonymized ID distinguishing each annotator) are provided for each annotation. Annotator demographic information is also included for transparency, if self-disclosed by the annotators. A complete list of annotations is provided in Supplementary Information A. Distribution plots showing the diversity of FHIBE are shown in Extended Data Figs. 1 and 2 and Supplementary Information B and D. The inter-rater reliability analysis, showing the high quality and consistency of FHIBE annotations, is shown in the Methods and Supplementary Information E.

Furthermore, FHIBE includes two derivative face datasets: a cropped-only set with 10,941 images from 1,981 subjects, and a cropped-and-aligned set with 8,370 images from 1,824 subjects. Both face datasets include all annotations.

### Comparison with existing datasets

We compare FHIBE against 27 human-centric computer vision datasets that have been used in fairness evaluations in Extended Data Table 1, considering their collection methods, annotations and ethical dimensions.

The majority of the datasets were scraped from Internet platforms or derived from scraped datasets. Seven well-known datasets were revoked by their authors and are no longer publicly available. Reasons for their removal are typically not stated explicitly, but point to growing criticism due to ethical challenges and concerns around web scraping data for AI development^37^. While a number of datasets have annotated bounding boxes, key points and segmentation masks, their pixel-level annotations do not match the density of FHIBE’s annotations. Datasets with dense pixel-level annotations, like COCO^40^, VQA2.0^41^ and MIAP^42^, contain only limited demographic information, none of which is self-reported.

Only four datasets mention that data were collected after obtaining consent from data subjects. CCv2^26^ and the Chicago Face Database^27^ are consent-based datasets, but provide no further details on how consent was obtained. While Dollar Street^43^ provides details on how consent was obtained, use in AI development was not stated as its purpose for collection, and there is no indication that the subjects consented to the processing of their biometric or other personal information. FHIBE stands out as the only dataset collected with robust consent for AI evaluation and bias mitigation.

FHIBE also has greater utility for diagnosing bias in AI compared with other consent-based datasets. CCv2 and Dollar Street have no pixel-level annotations. This makes them unsuitable for the diverse computer vision task evaluations that FHIBE enables. CCv2 and Chicago Face Database also only feature videos/images of individuals facing the camera, largely indoors, with only their head and shoulders shown. They lack full-body images and diverse backgrounds and poses, limiting their utility for many computer vision tasks, such as pose estimation, and for evaluating how models might perform in deployment contexts in which the individuals might not be looking at the camera.

Moreover, FHIBE stands out from other consent-driven datasets in terms of its detailed and self-reported demographic labels, which enable the investigation of model performance at complex intersections of demographic attributes (Table 1). Although CCv1 has 4.4 times more images and CCv2 has 2.8 times more subjects than FHIBE, FHIBE has 3.4 times more annotations and 16.9 times more attribute values (Table 2). FHIBE also has greater representation from regions that are under-represented in many computer vision datasets, such as Africa (44.7%) and lower-middle income economies (71.5%) (Table 3), making it uniquely suitable for bias evaluation.

**Table 1:** Dataset comparison by intersectional subgroups

Intersectional group	Dataset	Number of subgroups	Number of images		
			Med.	Max.	Min.
Gender × age	FHIBE	23	23	3,353	1
	CCv1	12	220	523	1
	CCv2	23	23	1,598	1
	FACET	9	2,070	22,008	3
	MIAP	4	7,439	21,195	254
Gender × age × skin tone	FHIBE	92	42	1,168	1
	CCv1	62	38	129	1
	CCv2	137	5	909	1
	FACET	82	284	12,506	1
Gender × age × ancestry region	FHIBE	72	128	8,415	4
Gender × age × ancestry subregion	FHIBE	322	31	1,683	1
Gender × age × ancestry region × skin tone	FHIBE	275	36	5,645	4
Gender × age × ancestry subregion × skin tone	FHIBE	1,234	9	1,129	1

This table shows how FHIBE compares with other fairness evaluation datasets based on intersectional groups, including gender or pronoun (only FHIBE uses pronouns), age, ancestry and skin tone. Subgroup counts and the median (med.), minimum (min.) and maximum (max.) number of images per subgroup are shown. FHIBE offers broader demographic representation through comprehensive annotations. Note that FACET and FHIBE images may be counted in multiple attribute categories if they have multiple/nested annotations (for example, multiple gender/pronoun or skin tone selections).

**Table 2:** Dataset comparison by summary statistics

	FHIBE	CCv1	CCv2	FACET	MIAP
Images/video frames	10,318	45,186	26,467	31,702	13,762
Subjects	1,981	3,011	5,567	NA	NA
Attributes	71	7	21	11	9
Attribute values	8,571	294	506	97	222

This table shows how FHIBE compares with other fairness evaluation datasets based on the number of images, number of unique subjects, number of annotated attributes (for example, skin tone, pronouns, ancestry) and number of unique attribute values (for example, six possible values for Fitzpatrick skin tone). MIAP excludes cases with unknown age or gender. FACET and MIAP lack subject identifiers (non-consensual datasets), resulting in a value of not applicable (NA) for the number of subjects. Despite having fewer images and subjects, FHIBE provides the highest number of attributes and attribute values.

**Table 3:** Dataset comparison by geographical region and income level

	FHIBE (%)	CCv1 (%)	CCv2 (%)	FACET (%)	COCO (%)	MIAP (%)
Africa	44.7	0.0	0.0	2.8	3.0	1.7
Asia and Oceania	40.6	0.0	49.8	36.2	11.4	14.3
Europe	4.4	0.0	0.0	49.8	34.2	36.2
Latin America and Caribbean	4.2	0.0	42.5	3.5	3.1	5.0
North America	6.0	100.0	7.7	7.7	48.3	42.8
High-income economies	11.5	100.0	7.7	54.0	89.1	87.5
Upper-middle-income economies	14.5	0.0	50.5	45.0	10.5	12.0
Lower-middle-income economies	71.5	0.0	41.8			
Low-income economies	0.3	0.0	0.0	0.9	0.4	0.5

Income groups are based on World Bank data. Geographical distributions for FACET, COCO and MIAP are estimates from a previous study^78^. These datasets combine upper-middle and lower-middle income levels into a single middle category. For the FHIBE dataset, the geographical distribution is derived from self-reported location annotations. Percentages for CCv1 and CCv2 are based on videos, while the other datasets use image counts.

## Ethical considerations to FHIBE design

In developing FHIBE, we sought to implement best practices for ethical data collection recommended in the literature^2,3,44^. We focused particularly on consent, privacy protection, compensation, safety, diversity and utility. The design decisions discussed below can also provide a starting point for future responsible data collection and curation efforts, including those not focused on fairness evaluation. Detailed descriptions of how these ethical considerations were implemented are provided in the Methods.

### Consent

Informed consent is central to research involving human participants, promoting participant safety and protection while supporting research integrity^19,45^. It involves the participants having sufficient information regarding the project and the potential risks before deciding to participate. Informed consent is also fundamental to data privacy protection, as encoded in various laws and regulations^7,18,19,46^.

Our consent processes were designed to comply with comprehensive data protection laws like the EU General Data Protection Regulation (GDPR)^46^. These processes included developing consent forms with clear language about the uses and disclosures of the collected data, the processing of biometric and sensitive data and the rights of data subjects with regard to their data. Policy considerations imbued in data privacy laws, such as respect for human dignity, also influenced other aspects of our data collection, including decisions regarding the types of attributes we collected (for example, pronouns rather than gender), participant recruitment guidelines (for example, no coercive practices) and restrictions on downstream uses of the dataset (for example, users are prohibited from attempting to reidentify subjects).

To ensure that consent is given on a voluntary basis^46^, data subjects retain control over their personal information and may withdraw their personal data from the dataset at any time, with no impact on the compensation they received from the project. In the event of consent withdrawal, we commit to maintaining dataset integrity by replacing withdrawn images and preserving the dataset’s size and diversity to the extent possible. This commitment makes FHIBE a first in computer vision—a living dataset designed to evolve responsibly.

### Privacy and IP

In addition to obtaining informed consent, we took additional measures to remove incidental personal information from the images. We used a state-of-the-art generative diffusion model^47^ to in-paint over non-consensual subjects (for example, individuals in the background of an image) and personally identifiable information (for example, license plates, credit cards). We then manually checked each image to verify the personal information had been removed, mitigating potential algorithmic biases in the automated methods^48^. This approach avoids the limitations of traditional privacy measures, such as automated face blurring^49^, which can still allow for reidentification through distinctive non-facial features (for example, tattoos, birthmarks)^50^. We further tested our method to ensure that it did not compromise the utility of the data for model evaluation. Moreover, we coarsened certain attributes and release others only in aggregate form.

To secure appropriate rights to license the images for downstream users, the participants submitting images were also required to review and agree to terms affirming they had the rights to provide the images and understood the nature of their contribution. Furthermore, our instructions to data vendors and participants included requirements to minimize the presence of third-party IP, such as trademarks and landmarks. We also implemented automated checks with manual verification to detect and exclude images with prominent third-party IP, such as logos, from our dataset.

### Compensation

Crowdworkers often contend with low wages and demanding working conditions^21,22^, while individuals whose images are included in web-scraped datasets receive no compensation. To address these concerns, we asked data vendors to report minimum payment rates per task per region and to compensate crowdworker participants—image subjects, annotators and QA annotators (definitions are provided in Supplementary Information C)—at least the applicable local minimum wage based on task-time estimates. Vendors’ reported minimum payment rates were cross-referenced against the International Labor Organization’s Global Wage Report^51^ or, where this was not applicable, with the minimum wage of a country with comparable GDP per capita. The median compensation for image subjects was 12× the applicable minimum wage (further information about project costs is provided in the Discussion and Methods).

### Safety

Webscraped datasets frequently include harmful and illegal content, ranging from derogatory annotations to instances of child sexual abuse material (CSAM)^2,6,17^. Although the risk of such content appearing in our dataset was low given our sourcing method, instructions to data subjects and vendor QA, we performed additional manual and automated checks to ensure safety. Each image was manually reviewed to identify and remove any harmful content and the image hashes were cross-referenced against a database of known CSAM maintained by the National Center for Missing & Exploited Children (NCMEC). This dual approach—leveraging both technology and human judgement—helped to create a dataset that is both safe and respectful of human dignity.

### Diversity

While diversity is a relevant consideration for data collection generally, the fact that FHIBE is a fairness evaluation set made it especially important to optimize for diversity across many dimensions: image subject demographics, appearance (for example, not wearing the same clothing in all images), poses, interactions between subjects and objects, and environment.

FHIBE contains detailed demographic information—such as age, pronouns and ancestry, making it possible to use FHIBE to evaluate model bias along many axes of interest. As FHIBE is a publicly available dataset, we sought to balance minimizing the disclosure of sensitive information while maximizing the availability of useful annotations for bias diagnosis. This led to our decision to collect pronouns, as pronouns are more likely to be public-facing information, while gender identity and sex can be quite sensitive, particularly for gender and sex minorities^52^. Moreover, while we collected information on data subjects’ disability status, pregnancy status, height and weight to measure the diversity of our dataset along these dimensions, we do not release these annotations with the dataset and only disclose the summary statistics in aggregate for transparency purposes (Supplementary Information B.1). Note that participant disclosures about pregnancy and disability status were optional.

Collecting pronouns rather than gender identity also reduced risks associated with misgendering^3,53^, and collecting ancestry offered a more stable alternative to country-specific racial categories^3,54^. We further describe the rationales to use pronouns and ancestry in Supplementary Information J.

We also collected annotations on phenotypic and performative markers to enhance bias analysis. Phenotypic attributes—like skin colour, eye colour and hair type—provide observable characteristics related to relevant demographic bias dimensions^9^, while performative markers—such as facial hair, cosmetics and clothing—help to identify social stereotypes and spurious correlations^55^. Moreover, FHIBE includes camera-level metadata and environmental annotations, capturing factors such as illumination, camera position and scene, which are important for understanding model performance across diverse conditions^16,56^.

With the exception of pixel-level annotations, head pose and camera distance, we focused on the collection of self-reported information to address the limitations (as discussed above) of previous data-collection efforts that used annotators to guess subjects’ attributes. Collecting self-reported attributes (as opposed to labelling them later) had the additional benefit of ensuring that the participants were well aware of the information about them that would be used in the project.

### Utility

An evaluation set is valuable only insofar as it enables assessments of model performance on relevant tasks. FHIBE provides extensive annotations for analysing human-centric visual scenes, including face- and person-specific bounding boxes, keypoints and segmentation masks. As a result, FHIBE can be used to evaluate models across a much wider variety of tasks than previously possible using consent-based computer vision datasets. Its combination of pixel-level annotations and attribute labels makes FHIBE to our knowledge the most comprehensively annotated fairness dataset currently available.

Moreover, we compared the utility of FHIBE as a fairness evaluation set with existing datasets. As discussed in the Methods, for each of the eight narrow model computer vision tasks that FHIBE was designed for, we evaluated commonly used models using FHIBE and pre-existing evaluation datasets (Supplementary Information F). The findings are discussed in the ‘Evaluation results’ section below.

## Evaluation results

### Bias discovery in narrow models

FHIBE’s diverse and comprehensive annotations provide both breadth and depth in fairness assessments, enabling the evaluation of models across a range of demographic attributes and their intersections. We examined the performance of a variety of pretrained narrow models—across eight common computer vision tasks: pose estimation, person segmentation, person detection, face detection, face parsing, face verification, face reconstruction and face super-resolution—on FHIBE’s demographic groups and their intersections (that is, pronoun × age group × ancestry × skin tone). The exact methodology is described in the Methods.

Through our benchmarking analysis, we found that intersectional groups combining multiple sensitive attributes—including pronoun, age, ancestry and skin tone—experience the largest performance disparities (Supplementary Fig. 21). Notably, despite the fact that skin tone is often used as a proxy for ancestry/race/ethnicity in fairness evaluations^57^, we find that intersections featuring both skin tone and ancestry have much greater disparities than those with only one of these attributes.

For each task, we also examined the intersectional groups for which the models showed the highest versus lowest disparity in performance. Note that, for this particular analysis, we considered only groups with at least ten subjects, and pairwise group comparisons were filtered using the Mann–Whitney *U*-test for statistical significance. To control for multiple comparisons, we applied Bonferroni correction^58^ by adjusting the significance threshold based on the number of pairwise tests, therefore considering only pairs with a statistically significant difference ($P < \frac{0.05}{{\rm{number}}\,{\rm{of}}\,{\rm{pairwise}}\,{\rm{tests}}}$). Through this analysis (Extended Data Table 2 and Supplementary Information K), we found that younger individuals (aged 18–29 years), those with lighter skin tones and those with Asian ancestry were more frequently among the groups that models performed best on, whereas older individuals (aged 50–59 and 60+ years), those with darker skin tones and those with African ancestry appeared more often among the groups that models performed worst on. However, despite these high-level trends, there was variability across models and specific intersections. For example, for face detection, RetinaFace performed best for ‘she/her/hers × type I × Asia’ and worst for ‘he/him/his × type II × Africa’, whereas MTCNN performed best for ‘she/her/hers × type II × Africa’ and worst for ‘he/him/his × type IV × Europe’.

This variability highlights the importance of testing for intersectional biases on a case-by-case basis, as bias trends can vary depending on the specific model–task combination. Overall, disparities likely arise from a combination of systemic biases—such as demographic under-representation—and task- or model-specific interactions with sensitive attributes. While some patterns align with broader structural inequalities, others reflect localized effects, emphasizing the need for nuanced and intersectional fairness assessments, which FHIBE’s extensive demographic annotations facilitate.

**Extended Data Fig. 3:**
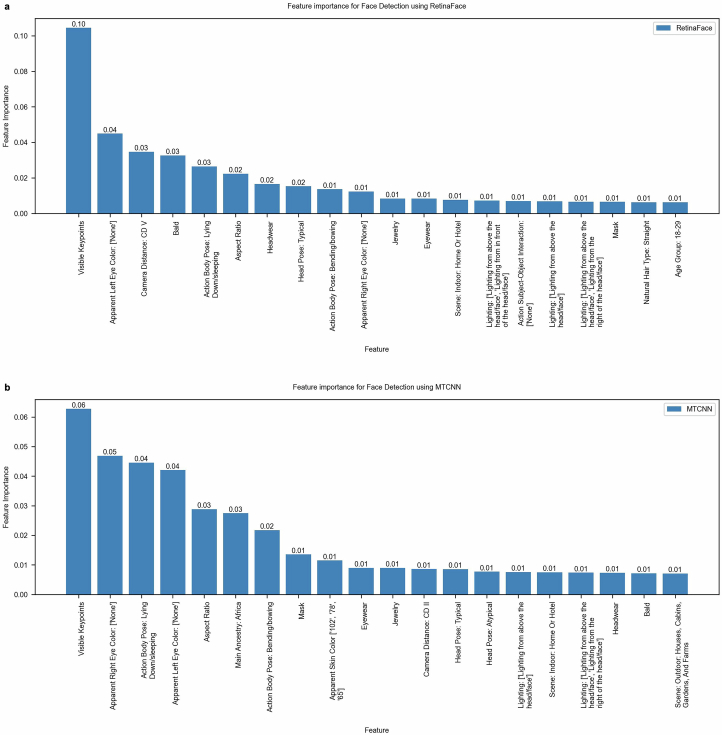
Feature importance for face detection. This figure shows feature importance scores extracted from random forest models for two face detection methods: (a) RetinaFace and (b) MTCNN. Features are ranked from most to least important, and the elbow method was applied to select the top-*K* attributes (*K* = 5 for RetinaFace, *K* = 4 for MTCNN) for use in decision tree models.

**Extended Data Fig. 4:**
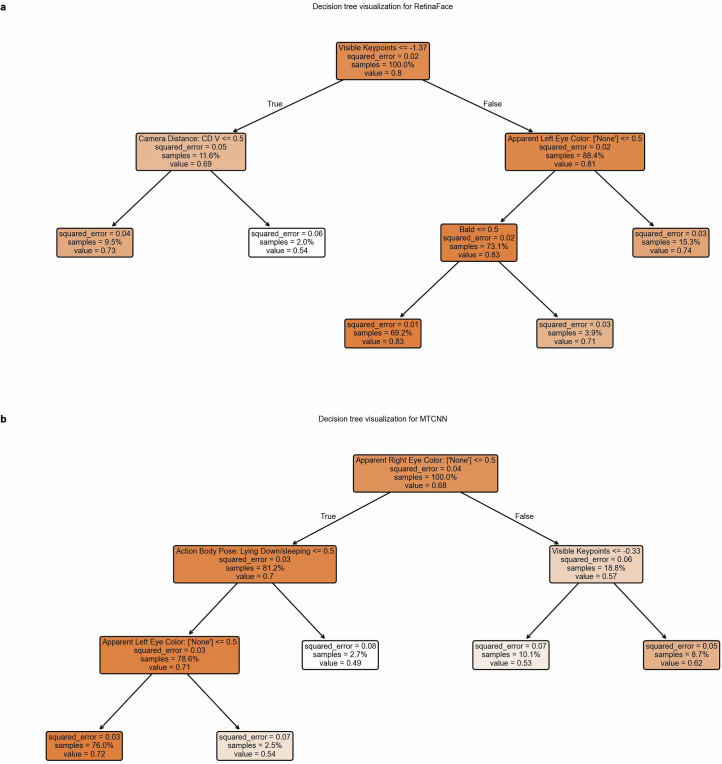
Decision tree models for face detection. This figure illustrates decision tree models for two face detection methods: (a) RetinaFace and (b) MTCNN. The models highlight key attributes predictive of face detection performance. Notably, attributes such as baldness have strong correlations with gender.

FHIBE further enables in-depth analyses of model performance disparities by identifying the specific features contributing to bias trends with greater granularity than what existing datasets facilitate. For example, we found that face-detection models showed consistently higher accuracy for individuals with she/her/hers pronouns compared with he/him/his pronouns (Supplementary Tables 14—16), a finding consistent with previous research^59^. Through our direct error modelling analysis, we used FHIBE’s extensive annotations to identify attributes that statistically significantly contributed to this performance difference (Extended Data Figs. 3 and 4).

**Extended Data Fig. 5:**
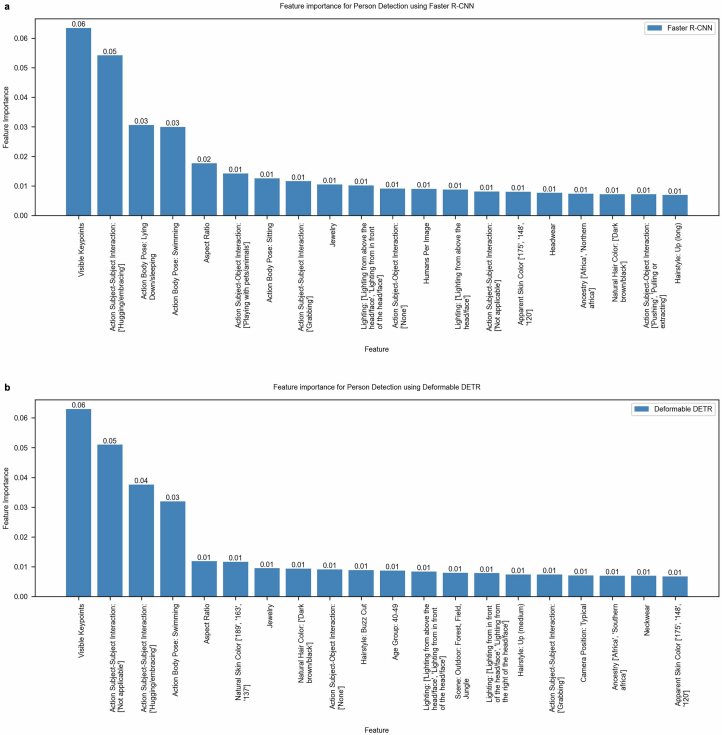
Feature importance for person detection. This figure shows feature importance scores extracted from random forest models for two person detection methods: (a) Faster R-CNN and (b) Deformable DETR. Features are ranked from most to least important. The elbow method was applied to select the top-*K* attributes (*K* = 5 for Faster R-CNN, *K* = 6 for Deformable DETR) for use in decision tree models. Lighting refers to the direction of head/face.

While many of the statistically significant attributes were not obviously related to gender (for example, visible keypoints, camera distance), lack of visible hair was a significant factor driving the gender disparity for RetinaFace (Extended Data Fig. 5). Further analysis conditioned on headwear and qualitative image inspection revealed that no visible hair in ‘he/him/his’ images often indicated baldness, making face detection challenging. Lack of visible hair was not only less common among ‘she/her/hers’ images, but it also typically resulted from headwear closely fitted to the face that preserved clear facial contours, making the task easier. FHIBE can therefore be used to help to explain underlying causes of previously identified biases.

Using FHIBE, we also identified previously unidentified bias trends. For example, face parsing models performed better for younger individuals than for older individuals (Supplementary Table 18). Through our error pattern recognition analysis, we found that much of this disparity was attributable to the models’ particularly poor performance for individuals with grey or white facial hair (Extended Data Fig. 7). For face verification, we conducted fairness evaluations using pretrained models—ArcFace^60^, CurricularFace^61^ and FaceNet^62^. The three mentioned models obtained lower accuracy for the ‘she/her/hers’ pronoun subgroup (Supplementary Table 20), a disparity that we traced to greater hairstyle variability (Extended Data Fig. 8) within this group—a factor that was previously overlooked when using less detailed datasets for bias diagnosis. This level of granularity in identifying the sources of bias can help to inform approaches to bias mitigation. For example, in this case, rather than collecting more training data from individuals of specific demographics, which can exacerbate ethical concerns around the ‘hypervisibility’ faced by certain marginalized groups^7^, a developer could focus on ensuring their face verification model is robust to hairstyle variability.

**Extended Data Fig. 6:**
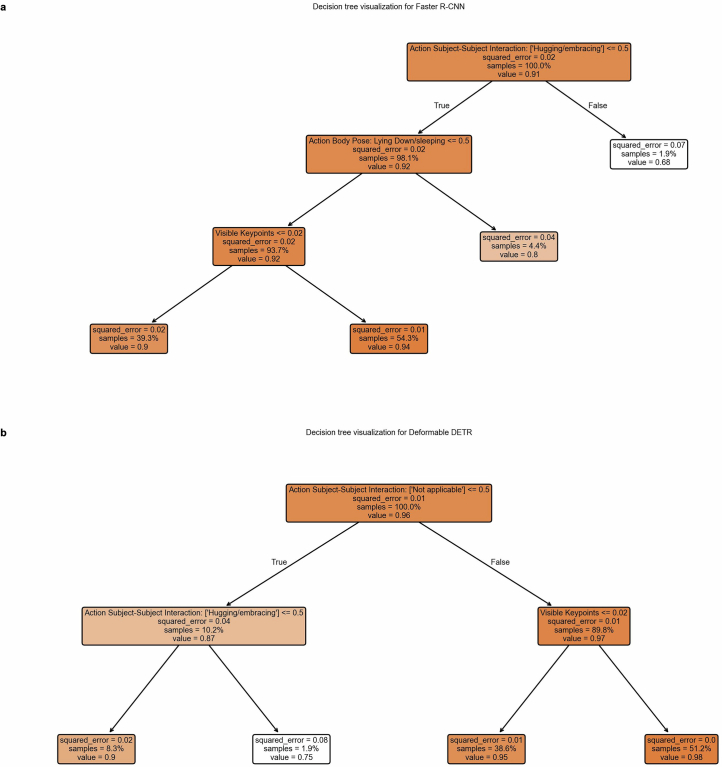
Decision tree models for person detection: Faster R-CNN and Deformable DETR. This figure illustrates decision tree models for person detection using (a) Faster R-CNN and (b) Deformable DETR. Notably, subject interactions, such as hugging or embracing, have a large impact on the performance of both models.

**Extended Data Fig. 7:**
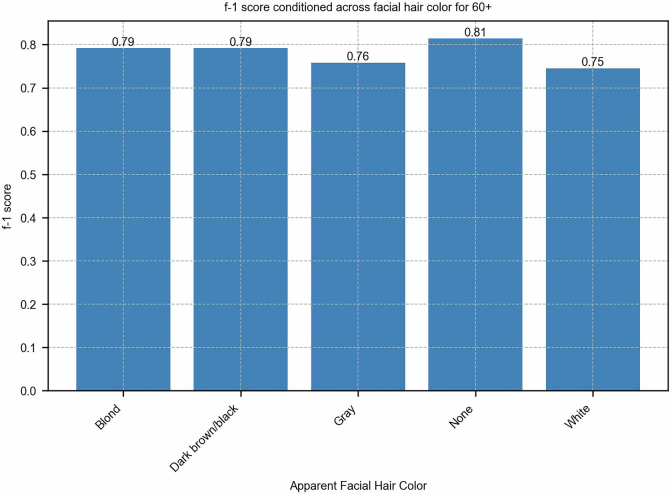
Face parsing performance by age and facial hair colour. This figure illustrates face parsing performance across facial hair colour categories for subjects aged 60+ years using the DML-CSR model. It highlights variations in model performance conditioned on facial hair colour, particularly for individuals with white facial hair.

**Extended Data Fig. 8:**
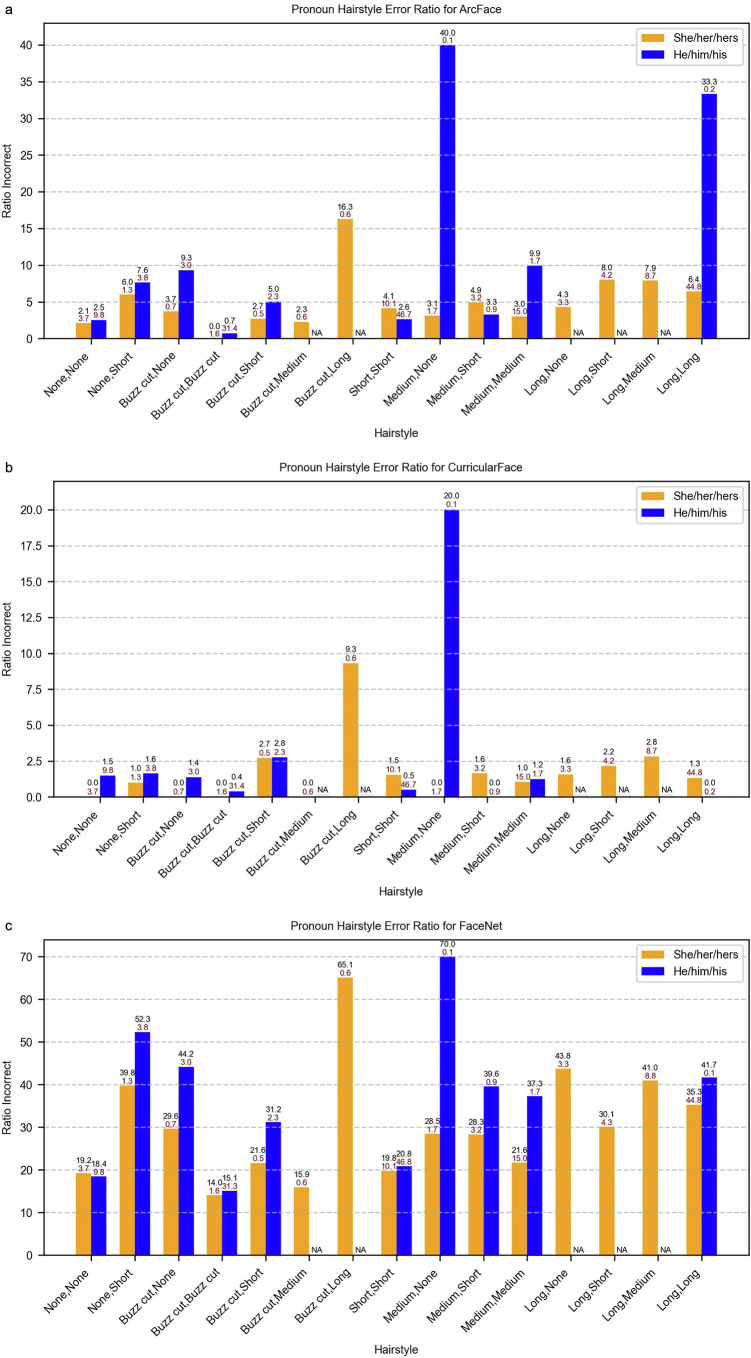
Error rates across hairstyle pairs for face verification models. This figure shows the percentage of incorrect predictions for face verification using (a) ArcFace^60^, (b) CurricularFace^61^, and (c) FaceNet^62^ models. For *He/Him/His* pronouns, errors are concentrated in cases with non-stereotypical hairstyles, whereas for *She/Her/Hers* pronouns, errors remain high whenever hairstyle variation within the pair is large. The number on top of each bar in black denotes the ratio of incorrect samples within that subgroup, while the number in red denotes the percentage of individuals with that pronoun who exhibit the corresponding hairstyle combination. This pattern highlights that hairstyle diversity disproportionately impacts error rates for She/Her/Hers pronouns. Error rates are conditioned on hairstyle changes and pronoun groups, underscoring variability in model performance.

Moreover, when assessing models using different observational datasets, conflicting bias trends often emerge. For example, in person-detection tasks, FHIBE found higher accuracy for individuals with darker skin tones, whereas FACET reported superior performance for lighter skin tones (Supplementary Tables 12 and 13). Leveraging FHIBE’s detailed annotations and our direct error modelling approach (Methods and Supplementary Information G), we identified confounding factors such as body pose (for example, lying down), subject interactions (for example, hugging/embracing), image aspect ratio and the number of visible keypoints (which indicate body occlusion) that significantly correlated with person-detection performance (Extended Data Figs. 5 and 6). To investigate these associations systematically, we applied a direct error modelling approach, using regression and decision trees to determine which features were linked to reduced model performance. In the case of faster-rcnn, our analysis identified the number of visible keypoints as a statistically significant factor in person-detection performance, with a higher count of visible keypoints leading to improved accuracy. When we analysed performance disparities by skin tone within a subset of images with a high number of visible keypoints, we found no statistically significant differences in performance across skin tones. This suggests that most performance disparities are driven by cases in which the subject’s keypoints are not fully visible, probably due to occluded body features.

These findings highlight the importance of addressing relevant sources of model errors and can guide developers in refining their models to enhance fairness and accuracy. FHIBE’s extensive annotations can provide valuable insights into the factors contributing to differences in fairness evaluation results across various benchmarks. FHIBE also enables developers to disentangle the source of bias among possible confounders. This is only possible with access to a rich set of accurate annotations, which FHIBE contains, but most comparable fairness evaluation datasets lack.

### Bias discovery in foundation models

Large-scale, multimodal generative models, which learn associations between text and images, enable diverse tasks such as classification, image search, image segmentation, image captioning and VQA (answering questions about an image). However, the widespread adoption of these technologies has also amplified their potential for harm. Research has shown that these models can perpetuate existing social biases^63^, reinforce harmful stereotypes^14,64^, and marginalize or dehumanize under-represented groups^65^.

Existing benchmarks for vision–language models (VLMs) focus mainly on improving performance in tasks such as object recognition^66^, robustness^67^ or reasoning^68^, and less on evaluating ethical dimensions such as bias and fairness^69^. Similar to datasets used to test narrow models, those that aim to evaluate VLM biases are often based on repurposed, web-scraped data^70,71^ leading to potential data leakage problems, limited coverage of societal dimensions^43,72^ and reliance on synthetically generated data that do not capture the nuances of real-world contexts^73^.

We demonstrate FHIBE’s utility for evaluating VLM foundation models across a range of image comprehension and recognition tasks. In particular, we assess two popular models, CLIP^74^ and BLIP-2^75^ (Methods and Supplementary Information H). We explored how pronoun and ancestry biases show up in general image understanding tasks like scene recognition (with CLIP) and open-ended VQA (with BLIP).

**Fig. 3:**
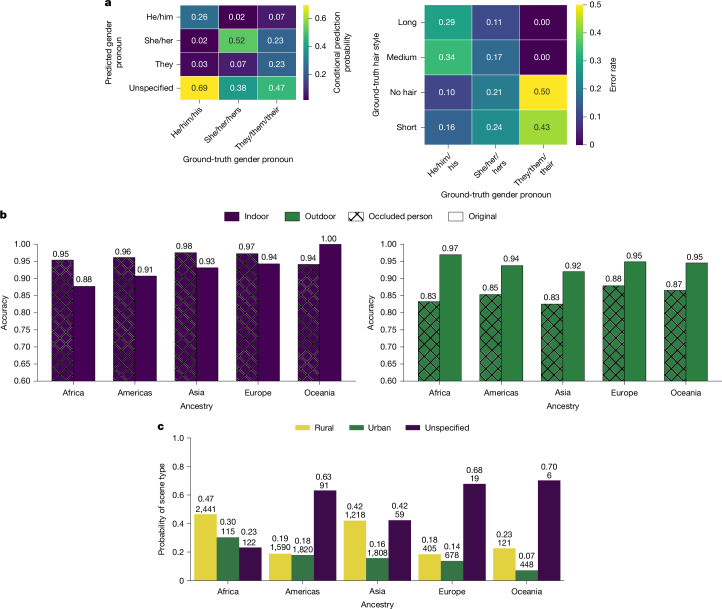
Biases in CLIP predictions on FHIBE. **a***,* Predicted label probabilities (rows) conditioned on ground-truth pronouns (columns) (left); CLIP more often assigns a gender-neutral ‘unspecified’ label to ‘he/him/his’ than to ‘she/her/hers’. Right, gender-classification error rates vary with both pronoun and hairstyle and are lowest for stereotypical pronoun–hairstyle combinations (for example, ‘he/him/his’ with ‘short/no hair’). **b**, For indoor environments, masking the person increases the accuracy, whereas, for outdoor environments, masking decreases the accuracy. This suggests that CLIP may treat the presence of a person as a spurious cue for outdoor scenes, with the effect being particularly pronounced for individuals of African ancestry. **c**, Scene type predictions conditioned on ancestry. CLIP is more likely to predict rural environments for images containing individuals of African or Asian ancestry. The numbers on each bar denote the group size (bottom) and the corresponding probability estimate (top), indicating that perceived rural associations are stronger for these groups.

When asked to classify images using 16 provided gender-related prompts (the prompts are provided in Supplementary Information H), we found that CLIP was far more likely to assign a gender-neutral label (unspecified) to those with ‘he/him/his’ pronouns (0.69) than those with ‘she/her/hers’ pronouns (0.38), reinforcing the idea that male individuals are the default people. Moreover, CLIP’s perception of gender was strongly influenced by hairstyle, with individuals who did not conform to stereotypical hairstyles (for example, ‘he/him/his’ pronouns and long hair) being frequently misgendered (Fig. 3a). We also found that CLIP had biased associations with other image attributes such as scene, disproportionately associating individuals of African ancestry with outdoor environments and linking those of African or Asian ancestry with rural settings (Fig. 3b,c).

**Fig. 4:**
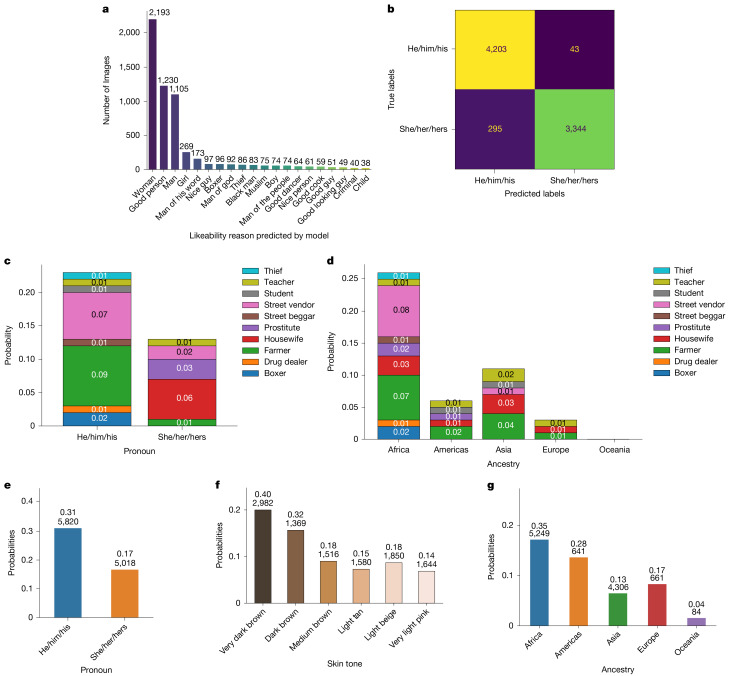
BLIP-2 analysis results. Summary of the gender, occupation, ancestry and toxic response analyses. **a**, Responses to non-gendered likeability prompts show implicit gender attribution. **b**, Pronoun predictions are more accurate for ‘he/him/his’ than ‘she/her/hers’, which exhibits a fivefold higher error rate. **c**,**d**, Neutral prompts about occupations highlight stereotypical associations, revealing gender-based (**c**) and ancestry-related (**d**) stereotypes. **e**–**g**, Negatively framed prompts elicit toxic responses linked to pronouns, skin tone and ancestry, with toxic gender-related responses (**e**), skin-tone-related responses (**f**) and ancestry-related responses (**g**). The numbers on each bar indicate the group size (bottom) and probability estimate (top).

Next, we assessed BLIP-2 in the VQA setting, prompting it with questions about the images with varying tones—positive, neutral or negative (the prompts are provided in Supplementary Information H). None of the prompts asked about or featured information about gender or ancestry. Nonetheless, we found that the model’s outputs still reflected biases based on gender and ancestry. For example, when asked why an individual is likeable, BLIP-2 frequently generated responses that attributed likability to gender, such as “because she is a woman” (Fig. 4a). As with CLIP, BLIP-2 was more likely to misgender individuals identified as ‘she/her/hers’ (Fig. 4b). Neutral prompts (for example, asking what an individual’s occupation is) sometimes produced benign output text (for example, teacher), but other times yielded terms that reinforced harmful stereotypes against specific pronoun and ancestry groups, such as prostitute, drug dealer and thief (Fig. 4c,d). Moreover, we found that negative prompts, for example, about what crimes an individual committed—which should yield a null response—elicited toxic responses at higher rates for individuals of African or Asian ancestry, those with darker skin tones and those identifying as ‘he/him/his’ (Fig. 4e–g).

Using FHIBE, we were therefore able to identify these previously undocumented biases. These observations underscore the persistent biases in these models and highlight the need for bias mitigation strategies.

## Discussion

FHIBE marks an inflection point in enabling the development of more responsible AI. Developers are able to evaluate and compare bias in their models across many computer vision tasks without relying on non-consensually sourced datasets. One of the key contributions of FHIBE is the implementation of many of the principles that have until now been advocated for only in responsible data curation, therefore paving the way for ethical data collection efforts going forward. Insights from the development of FHIBE also provide important learnings that can inform future directions for research.

Creating an ethics-driven human-centric dataset was challenging, as it required an investment into processes that are currently not the norm in the data-collection ecosystem. Overall, to arrive at the 10,318 images for the initial launch of FHIBE, we collected a total of 28,703 images from three data vendors, which cost nearly US$308,500 (average cost of US$10.75 per image). There were additional fixed costs of around US$450,000 for QA, legal services and the cost of building the data platform.

As this demonstrates, the emphasis on consent, fair compensation, rich annotations and global diversity made the data collection expensive. Furthermore, developing and implementing best practices for data collection, ensuring data quality and analysis further required the work of 25 researchers, engineers and project managers who worked at least part-time on the project at various points over the project’s 2–3 year lifespan, along with the extensive support of legal, privacy, IT and QA specialists.

At a time when there are growing calls for ethical data collection^21,22^ and realization of the importance of consent and compensation for data rightsholders^20^, transparency around the costs of data collection is critical for the AI community. Among the 27 human-centric computer vision datasets that we compare FHIBE with, only the Chicago Face Database^27^ provides information about the costs of data collection (they compensated participants US$20, and US$25 was randomly awarded to raters who completed a survey—compensation was given as gift cards). Data collection not featuring human subjects and personal information might be more cost-effective (for example, GeoDE^76^ cost US$1.08 per image for a 61,940-image dataset, not including researcher time). However, overall, the costs of consensual, diverse and fairly compensated data collection remain high considering the large amounts of data needed to train state of the art AI models^77^.

We hope that the practical learnings from FHIBE will help to inform future data collection efforts and encourage more research and investment into developing more scalable ethical data collection methods. As FHIBE is the first of its kind, future efforts can leverage our project as a starting point to substantially reduce the cost and time required, but there is still a need for further research on how to achieve ethical data collection methodologies at a scale that is suitable for AI training.

**Fig. 5:**
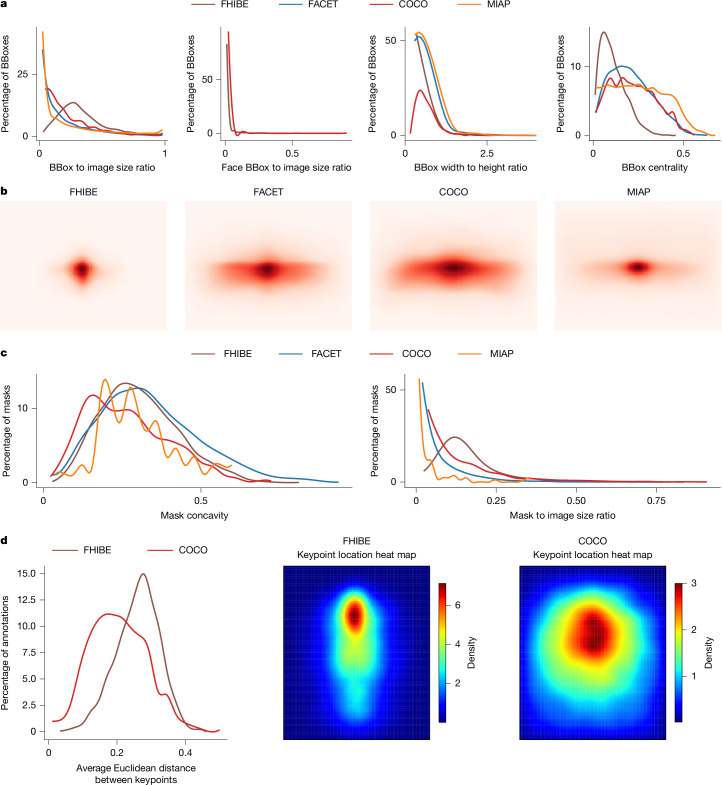
Dataset comparison based on bounding box, segmentation mask and keypoint properties. **a**, The bounding box (BBox) area to image area ratio; larger values indicate larger bounding boxes, suggesting that subjects are closer to the camera (left). Middle left, the face bounding box area to image area ratio; larger values indicate that subjects are closer to the camera. Only FHIBE and COCO were compared as the other datasets lack relevant labels. Middle right, the bounding box width to height ratio; values of <1 suggest that subjects are in vertical positions. Right, the normalized distance between the bounding box centre and image centre; smaller values indicate that the subjects are more centred. **b**, Person bounding box centre distributions. The centres are normalized by the image size to be in [0, 1]. FHIBE subjects are the most centred ones, with COCO and FACET demonstrating the largest spatial coverage. **c**, Person segmentation mask concavity, defined as $1-\frac{{\rm{mask}}\,{\rm{convex}}\,{\rm{area}}}{{\rm{image}}\,{\rm{area}}}$; higher values denote increased mask complexity (left). Right, person segmentation mask area to image area ratio; larger values indicate that subjects are closer to the camera (more detailed masks). Note that non-person categories are ignored. **d**, The average Euclidean distance between keypoint pairs; a greater distribution spread indicates a higher spatial coverage (left). Middle, heat map of FHIBE keypoint locations, showing a canonical shape with keypoints concentrated around standing humans centred in the image, with red density likely representing facial keypoints. Right, heat map of COCO keypoint locations, displaying a less canonical distribution, with keypoints more uniformly dispersed across the image, suggesting the presence of humans in diverse locations.

Aside from cost, compared with web-scraped datasets, there are some additional limitations to consensually collected datasets. Such datasets exhibit less visual diversity compared with web-scraped ones. As shown in Fig. 5, FHIBE’s pixel-level annotations are more standardized, that is, subjects are generally positioned closer to the camera and centred within the frame. FHIBE exhibits moderate segmentation complexity across a range of difficulty levels, but keypoints are predominantly visible and consistently distributed (Fig. 5). These factors probably contributed to models performing better on FHIBE than on web-scraped evaluation datasets for many tasks (Supplementary Information F). That said, FHIBE is much more visually diverse than other consent-based datasets, vastly surpassing both CCv1 and CCv2 (Table 4). Thus, FHIBE helps to bridge the gap between non-consensually and consensually sourced datasets, but future work should explore how to further close this gap.

**Table 4:** Comparison of dataset visual diversity

Dataset	Consent	Image	Scene (masked subject)	Subject (masked scene)
COCO	N	86.17	53.60	33.47
MIAP	N	**151.22**	**74.35**	**53.87**
FACET	N	93.53	62.67	27.48
CCv1	Y	28.65	–	–
CCv2	Y	41.92	–	–
FHIBE	Y	**69.61**	**31.18**	**28.70**

Vendi scores (Methods) for different datasets, categorized by consent requirements, where higher values indicate greater diversity. The image column reports Vendi scores for the original images; the scene (masked subject) column reports scores with human subjects masked by their person bounding boxes, indicating scene diversity; and the subject (masked scene) column reports scores with only the regions within subjects’ person bounding boxes visible, indicating subject diversity. The highest scores within each consent category are indicated in bold. Dashes indicate unavailable data due to missing pixel-level annotations. N, no; Y, yes.

Furthermore, crowdsourcing images made it difficult to verify that the person submitting the image was the same as the image subject. Through our automatic and manual quality checks, including reverse image search and examining consent forms and submission information, we identified possibly suspicious patterns and removed the corresponding images (Methods and Supplementary Information I). It is possible that core ethical considerations, such as fair compensation, increased the potential for fraudulent actors. For example, vendors generally offer higher compensation to demographic and geographical groups that are more difficult to collect consensual data from. This creates greater incentives for individuals to misrepresent themselves (despite the risk of being deplatformed by the vendor), to receive higher payments. It is therefore crucial for dataset curators to consider how their approaches to collecting diverse datasets ethically may attract potentially fraudulent actors. The potential for fraudulent actors is yet another reason for the importance of consent revocation and redaction in the context of ethical dataset collection. Future work should further consider the benefits and shortcomings of different data-collection approaches.

Despite the challenges of implementing a fair human-centric image benchmark, the FHIBE showcases that implementing core ethical considerations in practice is possible. We hope FHIBE will establish a new standard for responsibly curated data for AI systems by integrating comprehensive, consensually sourced images and annotations. FHIBE facilitates nuanced bias evaluations while avoiding many of the ethical concerns typical of modern datasets, particularly related to privacy and IP. Evaluations using FHIBE highlight pressing issues, such as performance disparities and stereotype reinforcement by AI models. By implementing responsible data practices and enabling the computer vision community to test their models for bias, FHIBE can help to enable the development of more inclusive and trustworthy AI systems.

## Methods

### Ethics statement: participants and consent/recruitment procedures

Data collection commenced after 23 April 2023, following Institutional Review Board approval from WCG Clinical (study number 1352290). All of the participants have provided their informed consent to the use of their data, and those who were image subjects further consented to have their identifiable images published.

We developed an informed consent form designed to comply with the EU’s GDPR^46^ and other similarly comprehensive data privacy regulations. Vendors were required to ensure that all image subjects (that is, both primary and secondary) provided signed informed consent forms when contributing their data. Vendors were also required to ensure that each image was associated with a signed copyright agreement to obtain the necessary IP rights in the images from the appropriate rightsholder. Only individuals above the age of majority in their country of residence and capable of entering into contracts were eligible to submit images.

All of the image subjects, regardless of their country of residence, have the right to withdraw their consent to having their images included in the dataset, with no impact to the compensation that they received for the images. This is a right that is not typically provided in pay-for-data arrangements nor in many data privacy laws beyond GDPR and GDPR-inspired regimes.

Data annotators involved in labelling or QA were given the option to disclose their demographic information as part of the study and were similarly provided informed consent forms giving them the right to withdraw their personal information. Some data annotators and QA personnel were crowdsourced workers, while others were vendor employees.

To validate English language proficiency, which was needed to understand the project’s instructions, terms of participation, and related forms, participants (that is, image subjects, annotator crowdworkers and QA annotator crowdworkers) were required to answer at least two out of three randomly selected multiple-choice English proficiency questions correctly from a question bank, with questions presented before project commencement. The questions were randomized to minimize the likelihood of sharing answers among participants. An example question is: “Choose the word or phrase which has a similar meaning to: significant” (options: unimportant, important, trivial).

To avoid possibly coercive data-collection practices, we instructed data vendors not to use referral programs to incentivize participants to recruit others. Moreover, we instructed them not to provide participants support (beyond platform tutorials and general technical support) in signing up for or submitting to the project. The motivation was to avoid scenarios in which the participants could feel pressured or rushed through key stages, such as when reviewing consent forms. We further reviewed project description pages to ensure that important disclosures about the project (such as the public sharing and use of the data collected, risks, compensation and participation requirements) were provided before an individual invested time into the project.

### Image collection guidelines

Images and annotations were crowdsourced through external vendors according to extensive guidelines that we provided. Vendors were instructed to only accept images captured with digital devices released in 2011 or later, equipped with at least an 8-megapixel camera and capable of recording Exif metadata. Accepted images had to be in JPEG or TIFF format (or the default output format of the device) and free from post-processing, digital zoom, filters, panoramas, fisheye effects and shallow depth-of-field. Images were also required to have an aspect ratio of up to 2:1 and be clear enough to allow for the annotation of facial landmarks, with motion blur permitted only if it resulted from subject activity (for example, running) and did not compromise the ability to annotate them. Each subject was allowed to submit a maximum of ten images, which had to depict actual subjects, not representations such as drawings, paintings or reflections.

Submissions were restricted to images featuring one or two consensual image subjects, with the requirement that the primary subject’s entire body be visible (including the head, and a minimum of 5 body landmarks and 3 facial landmarks identifiable) in at least 70% of the images delivered by each vendor, and the head visible (with at least 3 facial landmarks identifiable) in all images. Vendors were also directed to avoid collecting images with third-party IP, such as trademarks and landmarks.

To increase image diversity, we requested that images ideally be taken at least 1 day apart and recommended that images submitted of a subject were taken over as wide a time span as possible, preferably at least 7 days apart. If images were captured less than 7 days apart, the subject had to be wearing different clothing in each image, and the images had to be taken in different locations and at different times of day. Our instructions to vendors requested minimum percentages for different poses to enhance pose diversity, but we did not instruct subjects to submit images with specific poses. Participants were permitted to submit previously captured images provided that they met all requirements.

### Annotation categories and guidelines

We provided extensive annotation guidelines to data vendors that included examples and explanations. A complete list of the annotations, their properties (including whether they were multiple-choice), categories and annotation methods is provided in Supplementary Information A.

A key component of our project was that most annotations were self-reported by the image subjects as they were best suited to provide accurate information about subject demographics and physical characteristics, interactions depicted and scene context. The only annotations that were not self-reported were those that could be objectively observed from the image itself and would benefit from the consistency offered by professional annotators (that is, pixel-level annotations, head pose and camera distance, as defined by the size of an image subject’s face bounding box). We also provided examples and guidance for subject–subject interactions, subject–object interactions and head pose based on the request of our data vendors due to ambiguities in those labels.

We included open-ended, free text options alongside closed-ended responses, enabling subjects to provide input beyond predefined categories. These open-ended responses were coded as ‘Not Listed’. For privacy reasons, we do not report the specific text provided by the subjects. This approach enabled subjects to express themselves more fully^79,80^, resulting in more accurate data and informing better question design for future data collection. Given the mutability of most attributes, annotations were collected on a per-image basis, except for ancestry.

For the pixel-level annotations, face bounding boxes were annotated following the protocol used for the WIDER FACE dataset^81^, a commonly used face detection dataset. Keypoint annotations were based on the BlazePose topology^82^, a composite of the COCO^40^, BlazePalm^83^ and BlazeFace^84^ topologies. While the 17-keypoint COCO topology is widely used in computer vision, it lacks definitions for hand and foot keypoints, making it less suitable for applications such as fitness compared to BlazePose. For person segmentation, we defined 28 semantic segmentation categories based on the most comprehensive categorical schemas for this task, including MHP (v.2.0)^85^, CelebAMask-HQ^86^ and Face Synthetics^87^. Finally, person bounding boxes were automatically derived from human segmentation masks by enclosing the minimum-sized box that contained the entirety of each person’s segmentation mask.

Each annotator, QA annotator and QA specialist was assigned a unique identifier to link them to each annotation that they provided or reviewed, as well as any demographic information they chose to disclose. For annotation tasks involving multiple annotators, we provided the individual annotations from each annotator, rather than aggregated data. These annotations included those made before any vendor QA and those generated during each stage of vendor QA.

For our analyses, images with multiple annotations within a single attribute category (for example, ancestry subregion) are included in all relevant attribute value categories. For example, if an image subject is annotated with multiple ancestry subregions, the subject is counted in each of those subregions during analyses. Nested annotations—such as when a broad category is selected (for example, ‘Africa’ for ancestry)—are handled by counting the image subject in all corresponding subregions (for example, each subregion of ‘Africa’).

### Quality control and data filtering

Quality control for images and annotations was conducted by both the vendors and our team. Vendor QA annotators handled the first round of checking images, annotations, and consent and IPR forms. For non-self-reported annotations, vendor QA workers were permitted to modify the annotation if incorrect. For imageable attributes (such as apparent eye colour, facial marks, apparent head hair type), they could provide their own annotations if they believed the annotations were incorrect, but this would not overwrite the original self-reported annotation (we report both annotations). Vendors were instructed not to QA non-imageable attributes (such as pronouns, nationality, natural hair colour), with the exception of height and weight if there were significant differences in the numbers for the same subject in images taken 48 h or less apart.

Moreover, we developed and ran various automated and manual checks to further examine the images and annotations delivered by the vendors. Our automated checks verified image integrity (for example, readability), resolution, lack of post-processing artifacts and sufficient diversity among images of the same subject. They also assessed annotation reliability by comparing annotations to inferred data (for example, verifying that a scene labelled as ‘outdoor’ corresponds with outdoor characteristics in the image), checked for internal consistency (for example, ensuring body keypoints are correctly positioned within body masks), identified duplicates and checked the images against existing images available on the Internet. Moreover, the automated testing checked for CSAM by comparing image hashes against the database of known CSAM maintained by the National Center for Missing & Exploited Children (NCMEC).

Images containing logos were automatically detected using a logo detector^88^ and the commercial logo detection API from Google Cloud Vision^89^. They were then excluded from FHIBE to avoid trademark issues. We used a detection score threshold of 0.6 to eliminate identified bounding boxes with low confidence, and the positive detection results were reviewed and filtered manually to avoid false positives. However, despite these efforts, logo detection remains a complex challenge due to the vast diversity of global designs, spatial orientation, partial occlusion, background artifacts and lighting variations. Even manual review can be inherently limited, as QA teams cannot be familiar with every logo worldwide and often face difficulty distinguishing between generic text and logos. Our risk-based approach to logo detection and removal was informed by the relatively low risk of IP harms posed by the inclusion of logos in our dataset. The primary concern is that individuals might mistakenly perceive a relationship between our dataset and the companies whose logos appear. However, this is mitigated by the academic nature of this publication and the clear disclosure of author and contributor affiliations.

Manual checks on the data were conducted predominantly by our team of QA specialists, as well as by authors. The QA specialists were a team of four contractors who worked with the authors to evaluate the quality of vendor-delivered data, and conduct corrections where needed. The QA specialists had a background in ML data annotation and QA work, and received training and extensive documentation regarding the quality standards and requirements for images and annotations for this project. Furthermore, they remained in direct contact with our team throughout the project, ensuring that they could clarify quality standards as needed.

The manual checks focused on ensuring the accuracy of annotations for imageable attributes, such as hair colour, scene context and subject interactions. Non-imageable attributes, representing social constructs, such as pronouns or ancestry, were not part of the visual content verification. Moreover, even though the probability of objectionable content (for example, explicit nudity, violence, hate symbols) was low given our sourcing method, instructions to data subjects and QA from vendors, we took the additional step of manually reviewing each image for such content given the public nature of the dataset.

Overall, to arrive at the 10,318 images for the initial launch of FHIBE, we collected a total of 28,703 images from three data vendors. As the result of initial internal assessments, a set of 6,868 images were excluded due to issues with data quality and adherence to project specifications. Another 5,855 images were excluded for consent or copyright form issues. Of the remaining 15,980 images collected from vendors, approximately 0.07% were excluded for minor annotation errors (for example, missing skin colour annotations), 0.17% for offensive content (in free-text or visual content) and 0.01% for other reasons (for example, duplicated subject IDs) before the suspicious-pattern exclusions described in the following section.

### Detection and removal of suspicious images

It was difficult to determine whether the people who submitted the images were the same as the subjects in the image while respecting the privacy of the subjects. There can be fraudulent actors who submit images of other people without their consent to be compensated by data vendors. Given the public and consent-driven nature of our dataset, we did not rely exclusively on vendors to detect and remove suspicious images. We used a combination of automated and manual checks to detect and remove images where we had reason to suspect the data subject(s) might not be the individual who submitted the image. Combining automated and manual checks, we removed 3,848 images from 1,718 subjects from the dataset.

For automated checks, we used Web Detect from Google Cloud Vision API^89^ to identify and exclude images that could have been scraped from the Internet. This was a conservative check as images found online could still have been consensually submitted to our project by the image subject. However, given the importance of consent for our project, and the use of the dataset for evaluation, we excluded these images out of an abundance of caution.

This check resulted in removing 321 images, across 70 subjects, as we removed all the images for a given subject, as long as a single image was found online. However, there were some limitations to this automated approach. Vision API had a high false-positive rate: 62% for our task (that is, images that are visually similar, due to scene elements or popular landmarks). Google Web Detect returned limited results for images containing people and, in some cases, the returned matches focused on clothing items or the landmark. Furthermore, some social media images may not have been indexed by the Vision API because the websites required authentication.

We therefore also performed manual review methods for removing potentially suspicious images. Manual reviewers were instructed to track potentially suspicious patterns during their review of images and consent/copyright forms. For example, they were instructed to examine inconsistencies between self-reported and image metadata (for example, landmarks that contradicted the self-reported location). These patterns were later reviewed for exclusion by the research team.

Moreover, one of our QA specialists developed a manual process to find additional online image matches. The QA specialist used Google Lens to identify the location of the image. For images with distinctive locations (for example, not generic indoor locations or extremely popular tourist locations), the QA specialist performed a time-limited manual search to try to find image matches online. While we were not able to apply this time-intensive process to every image, using this approach, we were able to assess the risk level of different qualitative suspicious patterns and make additional exclusions.

After these exclusions, 2,017 subjects remained. From these subjects, we randomly sampled a set of 400 subjects and conducted the above manual QA process. In total, 14 subjects were found online while inspecting this sample, and we excluded them from the dataset. On the basis of this analysis, we estimated a baseline level of suspiciousness of 3.5 ± −1.7% with 95% confidence.

It is important to note that removing suspicious images also had an impact on the demographic distribution of subjects in the dataset (Supplementary Information I). We found that excluded images were more likely to feature individuals of older ages, with lighter skin tones and of Europe/Americas/Oceania ancestry. While it is not possible for us to determine the true underlying reason why some people might have submitted fraudulent images, we can speculate that some of the ethical design choices of our dataset may have inadvertently incentivized fraudulent behaviours. For example, requiring vendors to pay at least the applicable local minimum wage may have encouraged people to falsely claim to be from regions with higher wages, submitting images from the Internet taken in those locations. Similarly, in our pursuit of diversity, our vendors found certain demographics were more difficult to obtain images of (for example, people of older ages). As such, higher compensation was offered for those demographics, increasing the incentives to fraudulently submit images featuring those demographics.

The priorities of our data collection project also made fraud more feasible and difficult to detect. Given that FHIBE is designed for fairness evaluation, we sought to maximize visual diversity and collect naturalistic (rather than staged) images. As a result, we opted for a crowd-sourcing approach and allowed subjects to submit past photos. Compared with in-person data collection or bespoke data collection in which the setting, clothing, poses or other attributes might be fixed or specified, it was more difficult for our project to verify that the images were intentionally submitted by the data subject for our project. We therefore encourage dataset curators to consider how their ethical goals may inadvertently attract fraudulent submissions.

### Annotation QA

We verified the quality of both pixel-level annotations and imageable categorical attribute annotations using two methods. First, we compared the vendor-provided annotations with the average annotations from three of our QA specialists on a randomly sampled set of 500 images for each annotation type. For pixel-level annotations, agreement between the collected annotations and the QA specialist annotations was above 90% (Supplementary Information E), at a similar or higher level as related works^90–92^, showing the robustness and quality of our collected annotations.

Second, we assessed intra- and inter-vendor annotation consistency by obtaining three sets of annotations for the same 70 images from each vendor. Within each vendor, each image was annotated and reviewed three times by different annotators. To ensure independent assessments, no individual annotator reviewed the same annotation for a given image instance, resulting in mutually exclusive outputs from each labelling pipeline. For dense prediction annotations, intra- and inter-vendor agreement is above 90%, confirming a high quality of collected annotations. For attribute annotations, intra-vendor agreement is above 80% and inter-vendor agreement is at 70%, which indicates that they are more noisy labels than the dense prediction ones (Supplementary Information E).

Regarding metrics for these comparisons, for bounding boxes, we computed the mean intersection over union between the predicted and ground truth bounding boxes. For keypoints, we computed object keypoint similarity^93^. For segmentation masks, we computed the Sørensen–Dice coefficient^94,95^. For categorical attributes (for example, hair type, hairstyle, body pose, scene, camera position), we computed the pairwise Jaccard similarity coefficient^96^ and then the average. Using these analyses, we were able to verify the consistency of the annotations between vendors and our QA specialists, within individual vendors and between different vendors.

### Privacy assurance

We used a text-guided, fine-tuned stable diffusion model^47^ from the HuggingFace Diffusers library^97^ to inpaint regions identified by annotator-generated bounding boxes and segmentation masks containing incidental, non-consensual subjects or personally identifiable information (for example, license plates, identity documents). The model was configured with the following parameters: (1) text prompt: “a high-resolution image with no humans or people in it”; (2) negative text prompt: “human, people, person, human body parts, android, animal”; (3) guidance scale: randomly sampled from a uniform distribution, *w* ~ *U*(12, 16); (4) denoising steps: 20; and (5) variance control: *η* = 0, enabling the diffusion model to function as a denoising diffusion implicit model^98^.

We also manually reviewed the images to ensure the correct removal of personally identifiable information and identified any redaction artifacts. Around 10% of images had some content removed and in-painted. To evaluate any potential loss in data use, we compared performance on a subset of tasks (i.e., pose estimation, person segmentation, person detection and face detection) before and after removal and in-painting. No significant performance differences were observed.

To further address possible privacy concerns with the public disclosure of personal information, a subset of the attributes of consensual image subjects (that is, biological relationships to other subjects in a given image, country of residence, height, weight, pregnancy and disability/difficulty status) are reported only in aggregate form. Moreover, the date and time of image capture were coarsened to the approximate time and month of the year. Subject and annotator identifiers were anonymized, and Exif metadata from the images were stripped.

### Consent revocation

We are committed to upholding the right of human participants to revoke consent at any time and for any reason. As long as FHIBE is publicly available, we will remove images and other data when consent is revoked. If possible, the withdrawn image will be replaced with one that most closely matches key attributes, such as pronoun, age group and regional ancestry. To the extent possible, we will also consider other features that could impact the complexity of the image for relevant tasks when selecting the closest match.

### FHIBE derivative datasets

We release both the original images and downsampled versions in PNG format. The downsampled images were resized to have their largest side set to 2,048 pixels while maintaining the original aspect ratio. These downsampled versions were used in our analyses to prevent memory overflows when feeding images to the downstream models.

FHIBE also includes two face datasets created from the original images (that is, not the downsampled versions), both in PNG format: a cropped-only set and a cropped-and-aligned set. These datasets feature both primary and secondary subjects. For the cropped-and-aligned set, we followed a procedure similar to existing datasets^99,100^ by cropping oriented rectangles based on the positions of two eye landmarks and two mouth landmarks. These rectangles were first resized to 4,096 × 4,096 pixels using bilinear filtering and then downsampled to 512 × 512 pixels using Lanczos filtering^101^. Only faces with visible eye and mouth landmarks were included in the final cropped-and-aligned set.

For the cropped-only set, facial regions were directly cropped based on the face bounding box annotations, with each bounding box enlarged by a factor of two to capture all necessary facial pixels. This set includes images with resolutions ranging from 85 × 144 to 5,820 × 8,865 pixels. If facial regions extended beyond the original image boundaries, padding was applied using the mean value along each axis for both face derivative datasets.

### Datasets for fairness evaluation

We evaluated FHIBE’s effectiveness as a fairness benchmarking dataset by comparing it against several representative human-centric datasets commonly used in the computer vision literature. These datasets were selected based on their relevance to fairness evaluation, the availability of demographic annotations, and/or their use in previous fairness-related studies. Our analysis is limited to datasets that are publicly available; we did not include datasets that have been discontinued, like the JANUS program datasets (IJB-A, IJB-B, IJB-C, IJB-D)^102^. The results are shown in Supplementary Information F.

COCO is constructed from the MS-COCO 2014 validation split^40^, COCO Caption Bias^103^ and COCO Whole Body^104^ datasets. We used the images and annotations from the MS-COCO 2014 validation set, and added the perceived gender and skin tone (dark, light) annotations from COCO Caption Bias, excluding entries for which the label was ‘unsure’. We then used COCO Whole Body to filter the dataset for images containing at least one person bounding box. After filtering, this dataset contained 1,355 images with a total of 2,091 annotated person bounding boxes.

FACET^24^ is a benchmark and accompanying dataset for fairness evaluation, consisting of 32,000 images and 50,000 subjects, with annotations for attributes like perceived skin tone (using the Monk scale^105^), age group and perceived gender. For our evaluations, we used 49,500 person bounding box annotations and 17,000 segmentation masks, spread across 31,700 images.

Open Images MIAP^42^ is a set of annotations for 100,000 images from the Open Images Dataset, including attributes such as age presentation and gender presentation. In our evaluations, we used the test split, excluding images for which the annotations of age or gender are unknown, as well as the ‘younger’ category—to ensure that only adults were included in the evaluation. With this filtering, we used a set of 13,700 images with 36,000 associated bounding boxes and masks.

WiderFace^81^ is a face detection benchmark dataset containing images and annotations for faces, including the attributes perceived gender, age, skin tone, hair colour and facial hair. We used the validation split in our evaluations after excluding annotations for which perceived gender, age and skin tone were marked as ‘Not Sure’. After the filtering, we used a set of 8,519 face annotations across 2,856 files.

CelebAMask-HQ^86^ consists of 30,000 high-resolution face images of size 512 × 512 from the CelebA-HQ dataset, which were annotated with detailed segmentation of facial components across 19 classes. From this dataset, we used the test split in our evaluations, consisting of 2,824 images with binarized attributes for age, skin colour and gender.

CCv1^106^ contains 45,186 videos from 3,011 participants across five US cities. Self-reported attributes include age and gender, with trained annotators labelling apparent skin tone using the Fitzpatrick scale. For dataset statistics, we extracted a single frame per video. For Vendi score computation, we used 10 frames per video.

CCv2^26^ contains 26,467 videos from 5,567 participants across seven countries. Self-reported attributes include age, gender, language, disability status and geolocation, while annotators labelled skin tone (Fitzpatrick and Monk scales), voice timbre, recording setups and per-second activity. For dataset statistics, we extract a single frame per video. For Vendi score computation, we use three frames per video.

IMDB-WIKI^107^ is a dataset of public images of actors crawled from IMDB and Wikipedia. The images were captioned with date taken such that age could be labelled. From this dataset, we randomly sampled 10% to use for face verification task, resulting in 17,000 images.

### Narrow models for evaluation

To assess the use of FHIBE and FHIBE face datasets, we compared the performance of specialized narrow models (spanning eight classic computer vision tasks) using both FHIBE and pre-existing benchmark datasets as listed above. As FHIBE is designed only for fairness evaluation and mitigation, we did not train any models from scratch. Instead, we evaluated existing, pretrained state-of-the-art models on our dataset to assess their performance and fairness. The results are shown in Supplementary Information F.

Pose-estimation models aim to locate face and body landmarks in cropped and resized images derived from ground truth person bounding boxes, following^108–110^. For this task, we used Simple Baseline^108^, HRNet^109^ and ViTPose^110^, all of which were pretrained on the MS-COCO dataset^40^.

Person-segmentation models generate segmentation masks that label each pixel of the image with specific body parts or clothing regions of a person. For this task, we used Mask RCNN^111^, Cascade Mask RCNN^112^ and Mask2Former^113^, all of which were trained on MS-COCO dataset^40^.

Person-detection models identify individuals from images by relying on object detection models, retaining only the outputs for the class ‘person’. For this task, we used DETR^114^, Faster RCNN^115^, Deformable DETR^116^ and DDOD^117^ with the ResNet-50 FPN^115^ backbone, all of which were trained on MS-COCO dataset^40^.

Face-detection models locate faces in images by predicting bounding boxes that encompass each detected face. For this task, we used the MTCNN^118^ model trained on VGGFaces2^119^ and the RetinaFace^120^ model trained on WiderFace^81^ using publicly available source code^121,122^.

Face-segmentation models generate pixel-level masks that classify facial regions into specific facial features (such as eyes, nose, mouth or skin) or background, enabling detailed facial analysis and manipulation. For this task, we used the DML CSR^123^ model trained on CelebAMask-HQ^86^.

Face-verification models determine whether two face images belong to the same person by comparing their facial features against a preset similarity threshold. For extracting facial features, we used FaceNet^62^ trained on VGGFaces2^119^, and ArcFace^60^ and CurricularFace^61^, both trained on refined MS-Celeb-1M^124^, using publicly available implementations^61,121,125^.

Face-reconstruction models encode facial images into latent codes and decode these codes back into images, enabling controlled manipulation of facial attributes. For this task, we used ReStyle^126^ applied over e4e^127^ and pSp^128^, and trained on FFHQ^99^.

Face super-resolution models generate high-resolution facial images from low-resolution inputs, enhancing facial details and overall image quality. For this task, we used GFP-GAN^129^ and GPEN^130^, trained on FFHQ^99^.

### Narrow model evaluation metrics

We used the standard metrics reported in the literature to assess the performance of the narrow models on different tasks.

For pose estimation, we reported the percentage correct keypoints at a normalized distance of 50% of head length (PCK@0.5)^131^, which measures the portion of predicted landmarks (keypoints) falling within 0.5 × head-length radius from their true positions.

For person segmentation, person detection, and face detection, we reported the average recall across intersection over union (IoU) thresholds ranging [0.5, 0.95] with step size 0.05, to assess the average detection completeness of the models across multiple IoU thresholds.

For face segmentation, we reported the average F1 score (that is, the Sørensen–Dice coefficient^94,95^) across all segmentation mask categories, where F1 measures the intersection between the predicted and ground truth masks relative to their average size.

For face verification, we sampled image pairs of the same person (positive) and different people (negative) within each demographic subgroup. For each subgroup, we reported true acceptance rate (TAR) at a false acceptance rate (FAR) of 0.001. TAR@FAR = 0.001 measures the proportion of correctly accepted positive pairs when classification threshold is set to allow only 0.1% incorrectly accepted negative pairs.

For face reconstruction and face super-resolution, we reported learned perceptual image patch similarity^132^, which evaluates the perceived visual similarity between reference image *I*_ref_ and generated image *I*_gen_ by comparing their feature representations extracted by a pretrained VGG16^133^ model.

For face reconstruction, we also assessed perceptual quality using peak signal-to-noise ratio and measured identity preservation using cosine similarity between facial embeddings of *I*_ref_ and *I*_gen_ extracted by a CurricularFace model^61^.

### Dataset diversity

To compare FHIBE’s visual diversity with other datasets, we used the Vendi Score^134,135^, which quantifies diversity using a similarity function.

To construct the similarity matrix *K*, we first extracted image features (embeddings) using the self-supervised SEER^136^ model, which exhibits strong expressive power for vision tasks. We then constructed *K* by computing the cosine similarity between every feature pair. For extracting feature embeddings with SEER, all images are pre-processed using the ImageNet protocol: rescaling to 224 × 224 and applying *z*-score normalization using the ImageNet per-channel mean and s.d.

### Bias discovery in narrow models

We tested and compared FHIBE’s capabilities for bias diagnosis using a variety of methods.

#### Benchmarking analysis

For this analysis, we evaluated FHIBE on seven (note that for this analysis we excluded face verification owing to the inability to compute per-image scores for that task) different downstream computer vision tasks: pose estimation, person segmentation, person detection, face detection, face parsing, face reconstruction and face super-resolution. For each task and its respective models, we obtained a performance score for each image and subject, enabling us to conduct a post hoc analysis to explore the relationship between labelled attributes and performance.

For every task and model, we performed the following analyses. For each annotation attribute (for example, hair colour), we first isolated individual attribute groups (for example, blond, red, white). For each group, we compiled a set of performance scores (for example, scores for all subjects with blond hair, red hair or white hair). Only groups with at least ten subjects were considered in the analysis. We next performed pairwise comparisons (for example, blond versus red, blond versus white) using the Mann–Whitney *U*-test to determine whether the groups had similar median performance scores (null hypothesis, two-tailed). To control for multiple comparisons, we applied the Bonferroni correction^58^ by adjusting the significance threshold based on the number of pairwise tests. For pairs with a statistically significant difference ($P < \frac{0.05}{{\rm{number}}\,{\rm{of}}\,{\rm{pairwise}}\,{\rm{tests}}}$), we identified the groups with the lowest and highest median scores as the worst group and best group, respectively, and computed the min–max group disparity, *D*, between them:

$$D=1-\frac{{\rm{MED}}({\rm{worst}}\,{\rm{group}})}{{\rm{MED}}({\rm{best}}\,{\rm{group}})},\,\,D\in [0,1],$$

where MED(*g*) denotes the median performance score for group *g*. A value *D* → 0 indicates minimal disparity, while *D* → 1 indicates maximal disparity. We repeated this process for each attribute, identifying group pairs with statistically significant disparities and their corresponding values. For each attribute, we selected the pair with the highest disparity.

#### Direct error modelling

Using this approach, we aimed to examine which features were associated with reduced model performance using regression analysis. Although regression analysis is widely used to identify underlying relationships within datasets, its application to image data has traditionally been limited due to the lack of extensive structured annotations. However, the comprehensive scope and detail of the FHIBE annotations enabled us to effectively apply this method and achieve meaningful results. For each task and model, we predicted the model’s performance on individual images as the target variable. To this end, we collected, processed and extracted a range of annotations related to both images and subjects, including features derived from pixel-level annotations, such as the number of visible keypoints or visible head hair, or the absence of it (categorized as the binary attribute ‘bald’), which served as predictor variables. We used decision trees and random forests—an ensemble of decision trees—due to their interpretability, modelling power and low variance. We used the available implementation in the scikit-learn v.1.5.1 library for both of these models. Feature importance was obtained from the random forests model by assessing how each variable (for example, body pose) contributed to reducing variance when constructing decision trees, helping to identify the most predictive features. We then identified the most significant features (top six in most experiments) using the elbow method^137^. These selected features were then used in a decision tree model to assess the direction of their contribution to prediction—determining whether higher feature values are associated with better or worse model performance. To assess the robustness and statistical significance of observed differences across subgroups, we conducted bootstrap resampling with 5,000 iterations estimating standard errors. This approach enabled us to evaluate differences across groups even within smaller intersectional subgroups.

#### Error pattern recognition

We used association rule mining, a method frequently used in data mining to identify relationships between variables within a dataset. We applied association rule mining to identify attribute values that frequently co-occur with low performance. This approach enabled us to systematically identify and analyse patterns of bias within the model’s outputs. We used the FP-growth algorithm^138^. After obtaining the frequently occurring rules, we identified the attributes that are potential modes of error and investigated them further. We did this by studying the error disparities across the unique values of the attribute and evaluating its effect in conjunction with the sensitive attributes.

For face verification, we modified the protocol described above in the ‘Narrow model evaluation metrics’ section. Given that we wanted to look at the whole dataset, unconstrained to specific attributes, positive and negative pairs were computed using all face images from the FHIBE face dataset. All possible positive pairs were computed (15,474 pairs), while all negative pairs were sampled with the constraint as described previously^139^ to extract hard pairs: the gallery and probe images had the same pronoun, and their skin colour differed by no more than one of the six possible levels, yielding 4,945,896 pairs.

### Bias discovery in foundation models

Our analysis focuses on two foundation models: CLIP and BLIP-2. CLIP^74^ is a highly influential vision-language model that is widely recognized for its applications in zero-shot classification and image search. BLIP-2^75^ advances vision–language alignment by using a captioning and filtering mechanism to refine noisy web-scraped training data, thereby enhancing performance in image captioning, VQA and instruction following.

#### CLIP

We used the official OpenAI CLIP model^74^. We analysed CLIP in an open-vocabulary zero-shot setting to examine the model’s biases towards different image concepts, such as demographic attributes or image concepts (for example, scene). For each value of the given attribute, we presented four distinct text prompts. These prompts were intentionally varied in wording to reduce potential bias or sensitivity to specific phrasing. The prompts were standardized, clear and consistent across various values to minimize the influence of prompt engineering (the set of prompts is provided in Supplementary Information H). We further encoded FHIBE images using the CLIP image encoder. For pre-processing, we used the same pre-processing function as the official implementation. We analysed different variants of the FHIBE dataset to control for various effects related to the human subject and image background. These variants included the original images, images with individuals masked in black, images with individuals blurred with Gaussian noise of radius 100 and images with the background blacked out.

For the zero-shot classification analysis, we calculated the cosine similarity between the image embeddings and the text embeddings for each attribute. For example, for the scene attribute, we used two sets of prompts, each consisting of four text descriptions for indoor and outdoor environments. We computed the similarity between each text description and the image, selecting the description with the highest similarity as the assigned label for the image.

#### BLIP-2

The analysis protocol for the BLIP-2^75^ model consisted of specifying a prompt pair composed of an image and a text string in the form of a request or a question (the prompts are provided in Supplementary Information H). In our experiments, we instantiated the model to perform VQA tasks. We used the HuggingFace open-source BLIP-2 implementation (Salesforce/blip2-opt-2.7b) for all our bias analysis experiments. We defined a set of prompts that will be used to probe the model along with images drawn from the FHIBE dataset. Each prompt from the prompt set was used to probe the model and the final set of prompts was chosen to maximize the model response rate (that is, the prompts for which the model provided the most non-empty responses). The full set of prompts, their corresponding model response rate and the chosen prompts are provided in Supplementary Information H.

Each prompt from this chosen set was paired with every image from the FHIBE dataset and the pair was used to prompt the BLIP-2 model. Depending on the type of prompt, the model response was filtered to extract the relevant information. The filtering mechanism varied according to the prompt tone.

The positive prompts asked the model to determine the reason for likeability, while the neutral prompts asked the model for the person’s occupation based only on the provided image input. The model responses were in the form of ‘(S)he is likable because (s)he is a good person or (S)he’s a teacher’. To filter responses like these, we used regex expressions to isolate the predicted gender pronoun and the predicted occupation/likability reason from the model response.

When the model was prompted with the negatively toned prompt about convictions, the model responses included toxic and discriminatory language. The model response was labelled toxic if it contained any of the words contained in our keyword set as shown in the Supplementary Information H.

For this analysis, we prompted BLIP-2 with questions about individuals’ social attributes, but we do not condone the use of these tasks outside of bias detection. Predicting social attributes from facial images has long been a popular but problematic task in computer vision. For example, the ChaLearn First Impressions Challenge^140^ tasked participants with predicting personality traits like warmth and trustworthiness from videos or images. Deep learning models have been used to map facial features to social judgements^141,142^. With the rise of foundational models, such uses have also emerged for VQA models, which have been employed to predict personality traits of individuals from a single image of them^143^.

Such tasks are highly problematic due to their reliance on physiognomic beliefs that personality traits or social attributes can be inferred from appearance alone^144^. We use such tasks in our paper solely to identify biases in the model, not to use the model’s inferences themselves. While VQA models should in theory refuse to answer such questions, BLIP-2 generally did answer them, with its answers revealing learned societal biases. Building on recent efforts to identify biases in VQA models by using targeted questions to identify biases^145–147^ (for example, “Does this person like algebra?” and “Is this person peaceful or violent?”), our work shows how FHIBE can reveal biases in foundation models and cautions against the flawed assumptions they may promote.

### Reporting summary

Further information on research design is available in the Nature Portfolio Reporting Summary linked to this article.

## Online content

Any methods, additional references, Nature Portfolio reporting summaries, source data, extended data, supplementary information, acknowledgements, peer review information; details of author contributions and competing interests; and statements of data and code availability are available at 10.1038/s41586-025-09716-2.

## Supplementary information

Supplementary Information Supplementary Tables, Figures and Supplementary Discussion.

Reporting Summary

## Figures and Tables

**Extended Data Table 1:**
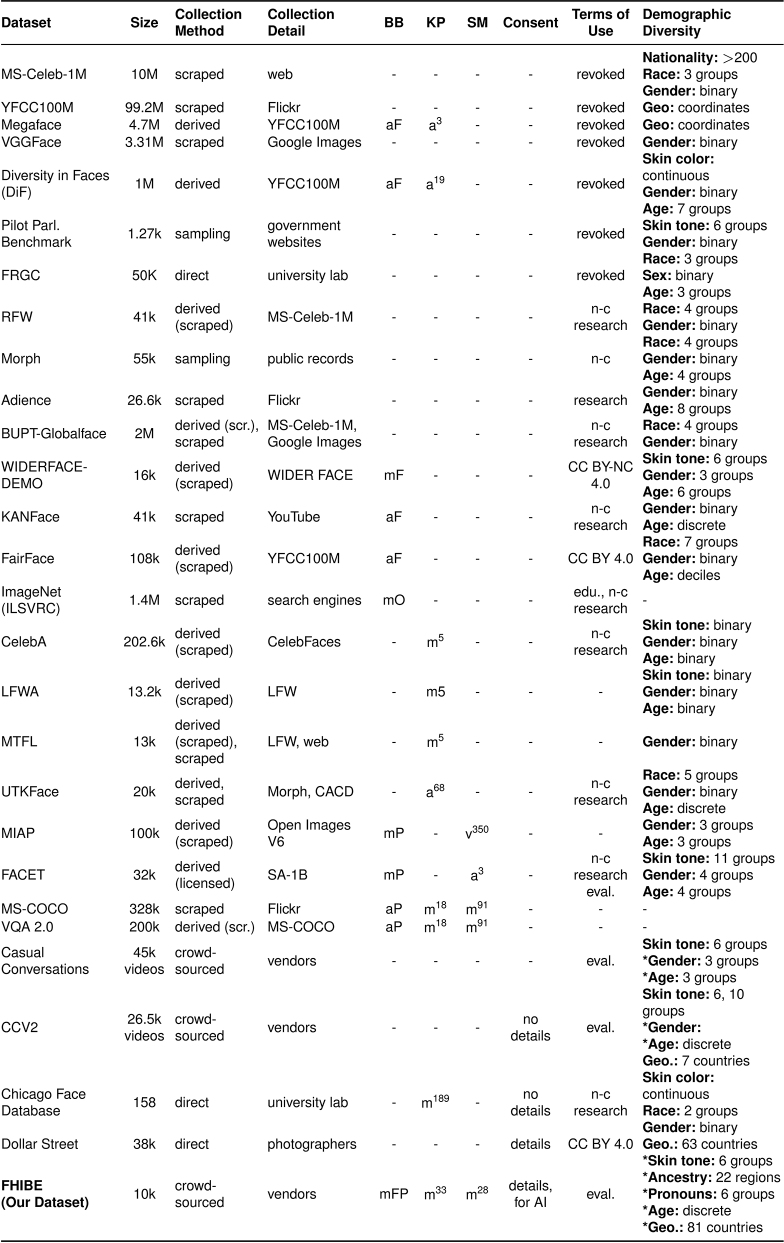
Overview of human-centric computer vision (HCCV) datasets commonly used for fairness

This table compares the properties of 27 HCCV datasets frequently used for evaluating bias in computer vision models. Features include dataset size, collection method, availability of annotations (Bounding Boxes [BB], Key Points [KP], Segmentation Masks [SM]), consent details, terms of use, and demographic diversity attributes. The abbreviations used are defined as follows: BB (a: automatic, m: manual, F: face, O: object, P: person), KP/SM (a: automatic, m: manual, v: manually verified, with the integer value denoting the number of key points or landmarks, or segmentation categories), Consent (no details: consent obtained, but no details provided; details: consent details provided, but no explicit mention of AI; details, for AI: consent details provided, including data processing for AI fairness purposes), and Terms of Use (n-c: non-commercial, research: research only, eval.: evaluation only, edu.: educational use, revoked: authors no longer make dataset available). Attributes marked with * are self-reported. (-) denotes where the relevant information was not available. MS-Celeb-1M^127^, YFCC100M^149^, Megaface^150^, VGGFace^151^, Diversity in Faces (DiF)^152^, Pilot Parl. Benchmark^9^, FRGC^153^, RWF^154^, Morph^155^, Adience^156^, BUPT-Globalface^157^, WIDERFACE-DEMO^158^, KANFace^159^, FairFace^160^, ImageNet (ILSVRC)^161^, CelebA^86^, LFWA^162^, MTFL^163^, UTKFace^164^, MIAP^42^, FACET^24^, MS-COCO^40^, VQA 2.0^41^, Casual Conversations^25^, CCV2^26^, Chicago Face Database^27^, Dollar Street^43^.

**Extended Data Table 2:**
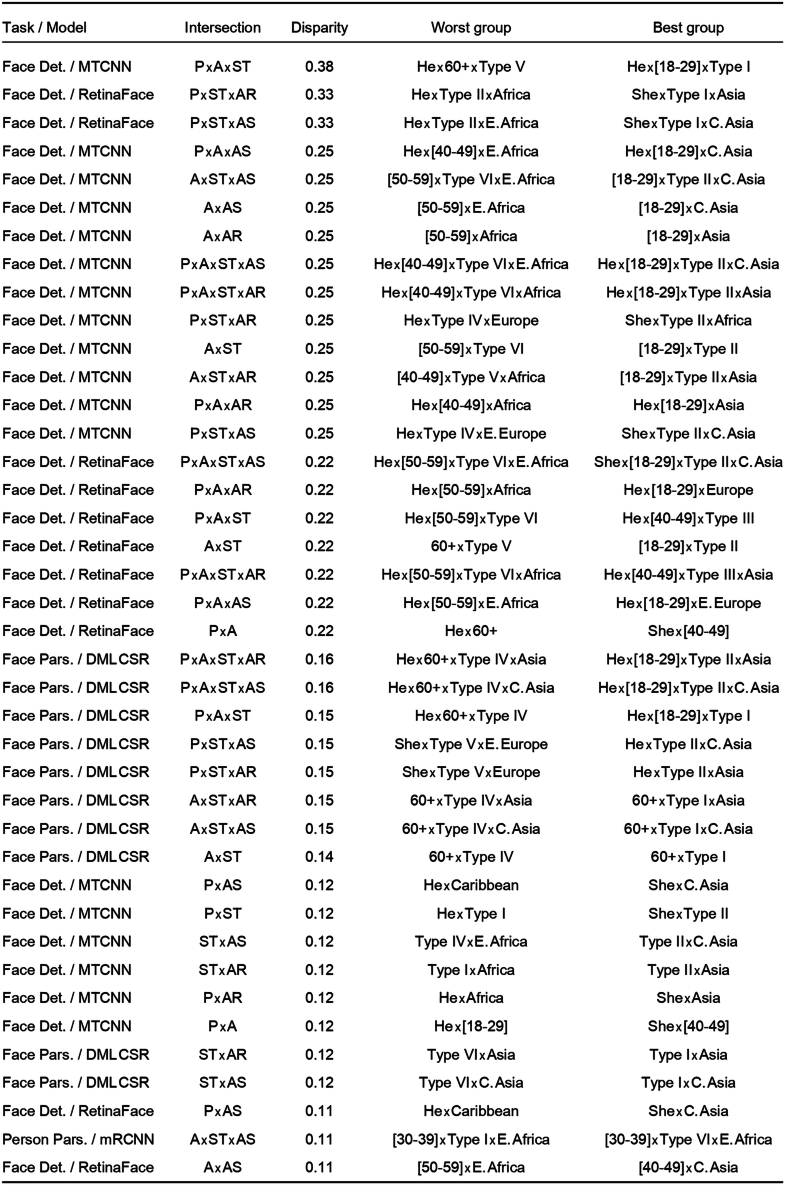
Top-40 (out of 278) cases of highest min-max intersectional group disparity across tasks and models

Disparity is defined as $1-\frac{MED(worst\,group)}{MED(best\,group)}$, with MED(*g*) being the median performance of group *g*. The table includes the task/model under evaluation, the intersectional attributes analysed, the disparity value, and the groups with the worst/best median performance. Abbreviations: **FD** (Face Detection), **FP** (Face Parsing), **PP** (Person Parsing), **mRCNN** (Mask R-CNN), **P** (Pronoun), **A** (Age), **AR** (Ancestry Region), **AS** (Ancestry Subregion), **ST** (Skin Tone in Fitzpatrick scale).

## Data Availability

The FHIBE dataset is publicly available at https://fairnessbenchmark.ai.sony. At this site, users are required to register an account with a valid email address and to agree to the terms of use, after which access is immediately provided. Such controls ensure that data protection terms and other legal provisions are agreed to and that notices and obligations related to the handling of the dataset can be communicated. The terms of use permit FHIBE to be used only for fairness/bias evaluation and mitigation purposes. FHIBE cannot be used for training, with the narrow exception of training bias mitigation tools. This restriction preserves the utility of FHIBE as an evaluation set (models cannot be first trained on and then evaluated on FHIBE). It also reduces potential harms, such as the use of the data to train prediction algorithms for sensitive (for example, gender, race, sexual orientation) or objectionable (for example, attractiveness, criminality) attributes or the reproduction of individuals’ likeness through being included in generative AI training sets. Individuals may request the removal of their data and the dataset will be updated and rereleased (to maintain size and diversity), as appropriate, in response to removal requests. Users with access to the dataset will then be notified and directed to delete portions of the dataset or to delete it in its entirety and use the updated version of the dataset, as required in our terms of use. Other datasets used in the study to compare FHIBE are listed in the Methods.

## Code availability

The code for running the fairness benchmarks across various computer vision tasks on the FHIBE dataset is publicly available on Github^148^ (https://github.com/SonyResearch/fairness-benchmark-public).

## References

[1] Sambasivan, N. et al. “Everyone wants to do the model work, not the data work”: data cascades in high-stakes AI. In *Proc. ACM CHI Conference on Human Factors in Computing Systems* (ACM, 2021).

[2] Birhane, A. & Prabhu, V. U. Large image datasets: a pyrrhic win for computer vision? In *Proc. IEEE Winter Conference on Applications of Computer Vision (WACV)* 1536–1546 (IEEE, 2021).

[3] Andrews, J. T. et al. Ethical considerations for collecting human-centric image datasets. In *Proc. Advances in Neural Information Processing Systems Datasets and Benchmarks Track (NeurIPS D&B) 55320–55360* (Curran Associates, 2023).

[4] Hundt, A., Agnew, W., Zeng, V., Kacianka, S. & Gombolay, M. Robots enact malignant stereotypes. In *Proc. ACM Conference on Fairness, Accountability, and Transparency (FAccT)* 743–756 (ACM, 2022).

[5] Wilson, B., Hoffman, J. & Morgenstern, J. Predictive inequity in object detection. In *Proc. IEEE/CVF Conference on Computer Vision and Pattern Recognition Workshops* (CVPRW) (IEEE, 2019).

[6] Birhane, A. et al. Into the LAION’s den: investigating hate in multimodal datasets. In *Proc. Advances in Neural Information Processing Systems Datasets and Benchmarks Track (NeurIPS D&B) 21268–21284* (2024).

[7] Xiang, A. Being ‘seen’ vs. ‘mis-seen’: tensions between privacy and fairness in computer vision. *Harvard J. Law Technol.* **36**, 1–60 (2022).

[8] Peng, K., Mathur, A. & Narayanan, A. Mitigating dataset harms requires stewardship: lessons from 1000 papers. In *Proc. Advances in Neural Information Processing Systems Datasets and Benchmarks Track (NeurIPS D&B)* (Curran Associates, 2021).

[9] Buolamwini, J. & Gebru, T. Gender shades: intersectional accuracy disparities in commercial gender classification. In *Proc.**ACM Conference on Fairness, Accountability, and Transparency (FAccT)* 77–91 (ACM, 2018).

[10] Bergman, A. S. et al. Representation in AI evaluations. In *Proc. ACM Conference on Fairness, Accountability, and Transparency (FAccT)* 519–533 (ACM, 2023).

[11] Holstein, K., Wortman Vaughan, J., Daumé III, H., Dudik, M. & Wallach, H. Improving fairness in machine learning systems: what do industry practitioners need? In *Proc. Conference on Human Factors in Computing Systems (CHI)* 1–16 (ACM, 2019).

[12] Deng, J. et al. Imagenet: a large-scale hierarchical image database. In *Proc. IEEE/CVF Conference on Computer Vision and Pattern Recognition (CVPR)* 248–255 (IEEE, 2009).

[13] Li, F.-F. & Krishna, R. Searching for computer vision North Stars. *Daedalus* **151**, 2 (2022).

[14] Lee, N. et al. Survey of social bias in vision-language models. Preprint at arxiv.org/abs/2309.14381 (2023).

[15] Andrus, M., Spitzer, E., Brown, J. & Xiang, A. What we can’t measure, we can’t understand: challenges to demographic data procurement in the pursuit of fairness. In *Proc. ACM Conference on Fairness, Accountability, and Transparency (FAccT)*249–260 (ACM, 2021).;

[16] Zhao, D., Andrews, J. T. A. & Xiang, A. Men also do laundry: multi-attribute bias amplification. In *Proc. 40th International Conference on Machine Learning* 42000–42017 (ACM, 2023).

[17] Thiel, D. *Identifying and Eliminating CSAM in Generative ML Training Data and Models* (Stanford Univ., 2023); 10.25740/kh752sm9123.

[18] Yew, R.-J. & Xiang, A. Regulating facial processing technologies: tensions between legal and technical considerations in the application of Illinois BIPA. *In Proc. ACM Conference on Fairness, Accountability, and Transparency (FAccT)* 1017–1027 (ACM, 2022).

[19] Politou, E., Alepis, E. & Patsakis, C. Forgetting personal data and revoking consent under the GDPR: challenges and proposed solutions. *J. Cybersec.* **4**, 001 (2018).

[20] Longpre, S. et al. A large-scale audit of dataset licensing and attribution in AI. *Nat. Mach. Intell.* **6**, 975–987 (2024).

[21] Gray, M. L. & Suri, S. *Ghost Work: How to Stop Silicon Valley from Building a New Global Underclass* (Eamon Dolan Books, 2019).

[22] Wang, D., Prabhat, S. & Sambasivan, N. Whose AI dream? In search of the aspiration in data annotation. In *Proc. 2022 CHI Conference on Human Factors in Computing Systems* 1–16 (ACM, 2022).

[23] Grother, P., Ngan, M., Hanaoka, K., Yang, J. C. & Hom, A. *Face Recognition Technology Evaluation (FRTE) Part 1: Verification* Technical Report (NIST, 2025); www.nist.gov/programs-projects/face-recognition-vendor-test-frvt-ongoing.

[24] Gustafson, L. et al. FACET: fairness in computer vision evaluation benchmark. In *Proc. International Conference on Computer Vision (ICCV)* 20370–20382 (IEEE, 2023).

[25] Hazirbas, C. et al. Casual conversations: a dataset for measuring fairness in AI. In *Proc. IEEE/CVF Conference on Computer Vision and Pattern Recognition Workshops (CVPRW)* 2289–2293 (IEEE, 2021).

[26] Porgali, B., Albiero, V., Ryda, J., Ferrer, C. C. & Hazirbas, C. The Casual Conversations v2 dataset. In *Proc. IEEE/CVF Conference on Computer Vision and Pattern Recognition* 10–17 (IEEE, 2023).

[27] Ma, D. S., Correll, J. & Wittenbrink, B. The Chicago Face Database: a free stimulus set of faces and norming data. *Behav. Res. Methods* **47**, 1122–1135 (2015). 25582810 10.3758/s13428-014-0532-5

[28] Dhar, P., Gleason, J., Roy, A., Castillo, C. D. & Chellappa, R. Pass: protected attribute suppression system for mitigating bias in face recognition. In *Proc.**IEEE/CVF Conference on Computer Vision and Pattern Recognition (CVPR)* 15087–15096 (IEEE, 2023); 10.1109/ICCV48922.2021.01481.

[29] Serna, I., Morales, A., Alonso-Fernandez, F. & Fierrez, J. Insidebias: measuring bias in deep networks using FairFaceVar. In *Proc.**IEEE/CVF Conference on Computer Vision and Pattern Recognition (CVPR)* 11071–11081 (IEEE, 2022).

[30] Yan, Z., Gong, S. & Hospedales, T. M. Mitigating demographic bias in face recognition via MultiFair representation. In *Proc. European Conference on Computer Vision (ECCV)* 1–18 (2022).

[31] Nagpal, S., Singh, M., Singh, R. & Vatsa, M. Deep learning for face recognition: pride or prejudiced? Preprint at arxiv.org/abs/1904.01219 (2019).

[32] Khan, Z. & Fu, Y. One label, one billion faces: usage and consistency of racial categories in computer vision. In *Proc. ACM Conference on Fairness, Accountability, and Transparency (FAccT)* 587–597 (ACM, 2021).

[33] Scheuerman, M. K., Paul, J. M. & Brubaker, J. R. How computers see gender: an evaluation of gender classification in commercial facial analysis services. *Proc. ACM Hum. Comput. Interact.* **3**, 1–33 (2019).34322658

[34] Keyes, O. The misgendering machines: trans/HCI implications of automatic gender recognition. *Proc. ACM on Hum. Comput. Interact.* **2**, 1–22 (2018).

[35] Hamidi, F., Scheuerman, M. K. & Branham, S. M. Gender recognition or gender reductionism? The social implications of embedded gender recognition systems. In *Proc. Conference on Human Factors in Computing Systems (CHI)* 1–13 (ACM, 2018).

[36] Wang, J., Liu, Y. & Levy, C. Fair classification with group-dependent label noise. In *Proc. ACM Conference on Fairness, Accountability, and Transparency (FAccT)* 526–536 (ACM, 2021).

[37] The rise and fall (and rise) of datasets. *Nat. Mach. Intell.* **4**, 1–2 (2022).

[38] *Standard Country or Area Codes for Statistical Use* (United Nations Department of Economic and Social Affairs, Statistics Division, 2024).

[39] Fitzpatrick, T. B. Soleil et peau. *J. Med. Esthet.* **2**, 33–34 (1975).

[40] Lin, T.-Y. et al. Microsoft COCO: common objects in context. In *Proc. European Conference on Computer Vision (ECCV)* 740–755 (Springer, 2014).

[41] Goyal, Y., Khot, T., Summers-Stay, D., Batra, D. & Parikh, D. Making the V in VQA matter: elevating the role of image understanding in visual question answering. In *Proc. IEEE/CVF Conference on Computer Vision and Pattern Recognition (CVPR) 16904-106913* (IEEE, 2017).

[42] Schumann, C., Ricco, S., Prabhu, U., Ferrari, V. & Pantofaru, C. R. A step toward more inclusive people annotations for fairness. In *Proc. AAAI/ACM Conference on AI, Ethics, and Society (AIES)* 916–925 (AAAI/ACM, 2021).

[43] Gaviria Rojas, W. et al. The Dollar Street dataset: images representing the geographic and socioeconomic diversity of the world. *Adv. Neur. Inform. Process. Syst.* **35**, 12979–12990 (2022).

[44] Gebru, T. et al. Datasheets for datasets. *Commun. ACM* **64**, 86–92 (ACM, 2021).

[45] *The Nuremberg Code. Trials of War Criminals Before the Nuremberg Military Tribunals Under Control Council Law no. 10* 181–182 (US Government, 1949).

[46] *General Data Protection Regulation* (European Commission, 2016); gdpr-info.eu/.

[47] Rombach, R., Blattmann, A., Lorenz, D., Esser, P. & Ommer, B. High-resolution image synthesis with latent diffusion models. In *Proc. Computer Vision and Pattern Recognition (CVPR)* 10684–10695 (IEEE, 2022).

[48] Luccioni, S., Akiki, C., Mitchell, M. & Jernite, Y. Stable bias: evaluating societal representations in diffusion models. In *Proc. 37th International Conference on Neural Information Processing Systems (NIPS)* 56338–56351 (Curran Associates, 2023).

[49] Yang, K., Yau, J.H., Fei-Fei, L., Deng, J. & Russakovsky, O. A study of face obfuscation in ImageNet. In *Proc. International Conference on Machine Learning (ICML)* 25313–25330 (PMLR, 2022).

[50] Orekondy, T., Fritz, M. & Schiele, B. Connecting pixels to privacy and utility: automatic redaction of private information in images. In *Proc. IEEE/CVF Conference on Computer Vision and Pattern Recognition (CVPR)* 8466–8475 (IEEE, 2018).

[51] *Global Wage Report 2020-21: Wages and Minimum Wages in the Time of COVID-19* 5–207 (International Labour Organization, 2020); www.ilo.org/wcmsp5/groups/public/---dgreports/---dcomm/---publ/documents/publication/wcms_762534.pdf.

[52] Thornton, S. & Tractenberg, R. E. Ethical considerations for data involving human gender and sex variables. In *Ethics in Statistics: Opportunities & Challenges* 260–291 (Ethics International Press, 2024).

[53] National Institutes of Health—Division of Program Coordination, Planning and Strategic Initiatives. *Gender Pronouns & Their Use in Workplace Communications* (2022); dpcpsi.nih.gov/sgmro/gender-pronouns-resource.

[54] Aspinall, P. J. Operationalising the collection of ethnicity data in studies of the sociology of health and illness. *Sociol. Health Illness* **23**, 829–862 (2001).

[55] Zhang, Y., Wang, J. & Sang, J. Counterfactually measuring and eliminating social bias in vision-language pre-training models. In *Proc. 30th ACM International Conference on Multimedia* 4996–5004 (ACM, 2022).

[56] Mitchell, M. et al. Model cards for model reporting. In *Proc. ACM Conference on Fairness, Accountability, and Transparency (FAccT)* 220–229 (ACM, 2019).

[57] Thong, W., Joniak, P. & Xiang, A. Beyond skin tone: a multidimensional measure of apparent skin color. In *Proc. IEEE/CVF International Conference on Computer Vision* 4903–4913 (IEEE, 2023).

[58] Bonferroni, C. Teoria statistica delle classi e calcolo delle probabilita. *Pubbl. R. Istitut. Super. Sci. Econ. Commer. Firenze* **8**, 3–62 (1936).

[59] Mittal, S., Thakral, K., Kartik, Majumdar, P., Vatsa, M. & Singh, R. Are face detection models biased? In *Proc. 2023 IEEE 17th International Conference on Automatic Face and Gesture Recognition (FG)* 1–7 (IEEE, 2023).

[60] Deng, J., Guo, J., Xue, N. & Zafeiriou, S. ArcFace: additive angular margin loss for deep face recognition. In *Proc. Computer Vision and Pattern Recognition (CVPR)* 4690–4699 (IEEE, 2019). 10.1109/TPAMI.2021.3087709 34106845

[61] Huang, Y. et al. Curricularface: adaptive curriculum learning loss for deep face recognition. In *Proc. Computer Vision and Pattern Recognition (CVPR)* 5901–5910 (IEEE, 2020).

[62] Schroff, F., Kalenichenko, D. & Philbin, J. FaceNet: a unified embedding for face recognition and clustering. In *Proc. Computer Vision and Pattern Recognition* 815–823 (IEEE, 2015).

[63] Wang, J., Liu, Y. & Wang, X. Are gender-neutral queries really gender-neutral? Mitigating gender bias in image search. In *Proc. 2021 Conference on Empirical Methods in Natural Language Processing* 1995–2008 (ACL, 2021).

[64] Bhargava, S. & Forsyth, D. Exposing and correcting the gender bias in image captioning datasets and models. Preprint at arxiv.org/abs/1912.00578 (2019).

[65] Agarwal, S. et al. Evaluating CLIP: towards characterization of broader capabilities and downstream implications. Preprint at arxiv.org/abs/2108.02818 (2021).

[66] Bordes, F. et al. Pug: photorealistic and semantically controllable synthetic data for representation learning. In *Proc. 37th International Conference on Neural Information Processing System*s (NIPS) 1952, 45020–45054 (Curran Associates, 2023).

[67] Li, X. et al. Imagenet-e: benchmarking neural network robustness via attribute editing. In *Proc. IEEE/CVF Conference on Computer Vision and Pattern Recognition* 20371–20381 (IEEE, 2023).

[68] Paiss, R. et al. Teaching clip to count to ten. In *Proc. IEEE/CVF International Conference on Computer Vision* 3170–3180 (IEEE< 2023).

[69] Lee, T. et al. VHELM: a holistic evaluation of vision language models. In *Proc. The Thirty-eight Conference on Neural Information Processing Systems (NIPS) Datasets and Benchmarks Track.* 4464, 140632–140666 (Curran Associates, 2025).

[70] Schumann, C., Ricco, S., Prabhu, U., Ferrari, V. & Pantofaru, C. A step toward more inclusive people annotations for fairness. In *Proc. 2021 AAAI/ACM Conference on AI, Ethics, and Society* 916–925 (AAAI/ACM, 2021).

[71] Krishna, R. et al. Visual genome: connecting language and vision using crowdsourced dense image annotations. *Int. J. Comput. Vis.* **123**, 32–73 (2017).

[72] Wu, X., Wang, Y., Wu, H.-T., Tao, Z. & Fang, Y. Evaluating fairness in large vision-language models across diverse demographic attributes and prompts. Preprint at arxiv.org/abs/2406.17974 (2024).

[73] Sathe, A., Jain, P. & Sitaram, S. A unified framework and dataset for assessing societal bias in vision-language models. In *Proc. Findings of the Association for Computational Linguistics: EMNLP 2024* 1208–1249 (ACL, 2024).

[74] Radford, A. et al. Learning transferable visual models from natural language supervision. In *Proc. International Conference on Machine Learning* 8748–8763 (PMLR, 2021).

[75] Li, J., Li, D., Savarese, S. & Hoi, S. Blip-2: bootstrapping language-image pre-training with frozen image encoders and large language models. *In Proc. International Conference on Machine Learning* 19730–19742 (PMLR, 2023).

[76] Ramaswamy, V. V. et al. Geode: a geographically diverse evaluation dataset for object recognition. In *Proc. 37th International Conference on Neural Information Processing Systems (NIPS)* 2888, 66127–66137 (Curran Associates, 2023).

[77] Hestness, J. et al. Deep learning scaling is predictable, empirically. Preprint at arxiv.org/abs/1712.00409 (2017).

[78] Kirillov, A. et al. Segment anything. In *Proc. IEEE/CVF International Conference on Computer Vision* 4015–4026 (IEEE, 2023).

[79] Singer, E. & Couper, M. P. Some methodological uses of responses to open questions and other verbatim comments in quantitative surveys. *mda* **11**, 115–134 (2017).

[80] Neuert, C., Meitinger, K., Behr, D. & Schonlau, M. The use of open-ended questions in surveys. *MDA* **15**, 3–6 (2021).

[81] Yang, S., Luo, P., Loy, C.-C. & Tang, X. Wider Face: a face detection benchmark. In *Proc. Conference on Computer Vision & Pattern Recogntion (CVPR)* 5525–5533 (IEEE, 2016).

[82] Bazarevsky, V. et al. Blazepose: on-device real-time body pose tracking. Preprint at arxiv.org/abs/2006.10204 (2020).

[83] Zhang, F. et al. MediaPipe Hands: on-device real-time hand tracking. Preprint at arxiv.org/abs/2006.10214 (2020).

[84] Bazarevsky, V., Kartynnik, Y., Vakunov, A., Raveendran, K. & Grundmann, M. BlazeFace: sub-millisecond neural face detection on mobile GPUs. Preprint at arxiv.org/abs/1907.05047 (2019).

[85] Zhao, J. et al. Understanding humans in crowded scenes: deep nested adversarial learning and a new benchmark for multi-human parsing. In *Proc. 26th ACM International Conference on Multimedia* 792–800 (ACM, 2018).

[86] Lee, C.-H., Liu, Z., Wu, L. & Luo, P. MaskGAN: towards diverse and interactive facial image manipulation. In *Proc. IEEE/CVF Conference on Computer Vision and Pattern Recognition (CVPR)* 5549–5558 (IEEE, 2020).

[87] Wood, E. et al. Fake it till you make it: face analysis in the wild using synthetic data alone. In *Proc. IEEE/CVF International Conference on Computer Vision (ICCV)* 3681–3691 (IEEE, 2021).

[88] Chen, H., Li, X., Wang, Z. & Hu, X. Robust logo detection in e-commerce images by data augmentation. In *Proc. 29th ACM International Conference on Multimedia* 4789–4793 (ACM, 2021).

[89] Google Cloud Vision API (Google, accessed 8 June 2023); cloud.google.com/vision/

[90] Papadopoulos, D. P., Uijlings, J. R., Keller, F. & Ferrari, V. Extreme clicking for efficient object annotation. In *Proc.**International Conference on Computer Vision (ICCV)* 4930–4939 (IEEE, 2017).

[91] Ruggero Ronchi, M. & Perona, P. Benchmarking and error diagnosis in multi-instance pose estimation. In *Proc.**International Conference on Computer Vision (ICCV)* 369–378 (IEEE, 2017).

[92] Boehringer, A. S., Sanaat, A., Arabi, H. & Zaidi, H. An active learning approach to train a deep learning algorithm for tumor segmentation from brain MR images. *Insights Imag.* **14**, 141 (2023). 10.1186/s13244-023-01487-6 PMC10449747 37620554

[93] *COCO Keypoints Evaluation* (COCO Consortium, 2016); cocodataset.org/#keypoints-eval.

[94] Dice, L. R. Measures of the amount of ecologic association between species. *Ecology* **26**, 297–302 (1945).

[95] Sorensen, T. A method of establishing groups of equal amplitude in plant sociology based on similarity of species content and its application to analyses of the vegetation on danish commons. *Biol. Skrift.* **5**, 1–34 (1948).

[96] Jaccard, P. The distribution of the flora in the Alpine Zone. 1. *N. Phytol.* **11**, 37–50 (1912).

[97] Platen, P. et al. Diffusers: state-of-the-art diffusion models. (GitHub, 2022).

[98] Song, J., Meng, C. & Ermon, S. Denoising diffusion implicit models. In *Proc. International Conference on Learning Representations (ICLR)* (2021); openreview.net/forum?id=St1giarCHLP.

[99] Karras, T., Laine, S. & Aila, T. A style-based generator architecture for generative adversarial networks. In *Proc. Computer Vision and Pattern Recognition (CVPR)* 4217–4228 (2019). 10.1109/TPAMI.2020.2970919 32012000

[100] Karras, T., Aila, T., Laine, S. & Lehtinen, J. Progressive growing of GANs for improved quality, stability, and variation. In *Proc. International Conference on Learning Representations (ICLR)* 1–26 (2018); openreview.net/forum?id=Hk99zCeAb.

[101] Duchon, C. E. Lanczos filtering in one and two dimensions. *J. App. Meteorol. Climatol.* **18**, 1016–1022 (1979).

[102] *Technology Face Challenges* (NIST, 2024); www.nist.gov/programs-projects/face-challenges.

[103] Zhao, D., Wang, A. & Russakovsky, O. Understanding and evaluating racial biases in image captioning. In *Proc. International Conference on Computer Vision (ICCV)* 14830–14840 (IEEE, 2021).

[104] Jin, S. et al. Whole-body human pose estimation in the wild. In *Proc. European Conference on Computer Vision (ECCV)* 196–214 (Springer, 2020).

[105] Monk Jr, E. P. The cost of color: skin color, discrimination, and health among African-Americans. *Am. J. Sociol.* **121**, 396–444 (2015). 10.1086/682162 26594713

[106] Hazirbas, C. et al. Towards measuring fairness in AI: the casual conversations dataset. *IEEE Trans. Biometr. Behav. Iden. Sci.* **4**, 324–332 (2021).

[107] Rothe, R., Timofte, R. & Gool, L. V. Dex: deep expectation of apparent age from a single image. In *Proc. IEEE International Conference on Computer Vision Workshops (ICCVW)* (IEEE, 2015).

[108] Xiao, B., Wu, H. & Wei, Y. Simple baselines for human pose estimation and tracking. In *Proc. European Conference on Computer Vision (ECCV)* 466–481 (Springer, 2018).

[109] Sun, K., Xiao, B., Liu, D. & Wang, J. Deep high-resolution representation learning for human pose estimation. In *Proc. Computer Vision and Pattern Recognition (CVPR)* 5693–5703 (IEEE, 2019).

[110] Xu, Y., Zhang, J., Zhang, Q. & Tao, D. ViTPose: simple vision transformer baselines for human pose estimation. In *Proc. 36th International Conference on Neural Information Processing Systems (NIPS)* 2795, 38571–38584 (Curran Associates, 2022).

[111] He, K., Gkioxari, G., Dollár, P. & Girshick, R. Mask R-CNN. In *Proc. International Conference on Computer Vision (ICCV)* 2961–2969 (IEEE, 2017).

[112] Cai, Z. & Vasconcelos, N. Cascade R-CNN: high quality object detection and instance segmentation. *IEEE Trans. Pattern Anal. Mach. Intell.* **43**, 1483–1498 (2019). 10.1109/TPAMI.2019.2956516 31794388

[113] Cheng, B., Misra, I., Schwing, A. G., Kirillov, A. & Girdhar, R. Masked-attention mask transformer for universal image segmentation. In *Proc. Computer Vision and Pattern Recognition (CVPR)* 1290–1299 (IEEE, 2022).

[114] Carion, N. et al. End-to-end object detection with transformers. In *Proc. European Conference on Computer Vision (ECCV)* 213–229 (Springer, 2020).

[115] Ren, S., He, K., Girshick, R. & Sun, J. Faster R-CNN: towards real-time object detection with region proposal networks. In *Proc. 29th International Conference on Neural Information Processing Systems (NIPS)* 1, 91–99 (MIT Press, 2015).

[116] Zhu, X. et al. Deformable DETR: deformable transformers for end-to-end object detection. In *Proc. International Conference on Learning Learning Representations (ICLR)* (2021).

[117] Chen, Z. et al. Disentangle your dense object detector. In *Proc. 29th ACM International Conference on Multimedia* 4939–4948 (ACM, 2021).

[118] Xiang, J. & Zhu, G. Joint face detection and facial expression recognition with MTCNN. In *Proc. 2017 4th International Conference on Information Science and Control Engineering (ICISCE)* 424–427 (IEEE, 2017).

[119] Cao, Q., Shen, L., Xie, W., Parkhi, O. M. & Zisserman, A. VGGFace2: a dataset for recognising faces across pose and age. In *Proc. International Conference on Automatic Face and Gesture Recognition* 67–74 (IEEE, 2018).

[120] Deng, J., Guo, J., Ververas, E., Kotsia, I. & Zafeiriou, S. Retinaface: single-shot multi-level face localisation in the wild. In *Proc. Computer Vision and Pattern Recognition (CVPR)* 5203–5212 (IEEE, 2020).

[121] facenet-pytorch contributors. Face recognition using PyTorch (2019); github.com/timesler/facenet-pytorch.

[122] FaceXLib contributors. FaceXLib (2021); github.com/xinntao/facexlib.

[123] Zheng, Q., Deng, J., Zhu, Z., Li, Y. & Zafeiriou, S. Decoupled multi-task learning with cyclical self-regulation for face parsing. In *Proc. Computer Vision and Pattern Recognition (CVPR)* 4156–4165 (IEEE, 2022).

[124] Guo, Y., Zhang, L., Hu, Y., He, X. & Gao, J. Ms-celeb-1m: a dataset and benchmark for large-scale face recognition. In *Proc. European Conference on Computer Vision (ECCV)* 87–102 (Springer, 2016).

[125] Wang, Q., Zhang, P., Xiong, H. & Zhao, J. Face.evoLVe: a high-performance face recognition library. *Neurocomputing* **494**, 443–445 (2022).

[126] Alaluf, Y., Patashnik, O. & Cohen-Or, D. Restyle: a residual-based stylegan encoder via iterative refinement. In *Proc. International Conference on Computer Vision (ICCV)* 6711–6720 (IEEE, 2021).

[127] Tov, O., Alaluf, Y., Nitzan, Y., Patashnik, O. & Cohen-Or, D. Designing an encoder for stylegan image manipulation. *ACM Trans. Graph.* **40**, 1–14 (2021).

[128] Richardson, E. et al. Encoding in style: a StyleGAN encoder for image-to-image translation. In *Proc. Computer Vision and Pattern Recognition (CVPR)* 2287–2296 (IEEE, 2021).

[129] Wang, X., Li, Y., Zhang, H. & Shan, Y. Towards real-world blind face restoration with generative facial prior. In *Proc. Computer Vision and Pattern Recognition (CVPR)* 8494–8508 (IEEE, 2021).

[130] Yang, T., Ren, P., Xie, X. & Zhang, L. GAN prior embedded network for blind face restoration in the wild. In *Proc. Computer Vision and Pattern Recognition (CVPR)* 672–681 (IEEE, 2021).

[131] Andriluka, M., Pishchulin, L., Gehler, P. & Schiele, B. 2D human pose estimation: new benchmark and state of the art analysis. In *Proc. IEEE/CVF Conference on Computer Vision and Pattern Recognition (CVPR)* 3686–3693 (IEEE, 2014).

[132] Zhang, R., Isola, P., Efros, A.A., Shechtman, E. & Wang, O. The unreasonable effectiveness of deep features as a perceptual metric. In *Proc. Computer Vision and Pattern Recognition (CVPR)* 586–595 (IEEE, 2018).

[133] Simonyan, K. & Zisserman, A. Very deep convolutional networks for large-scale image recognition. In *Proc. 3rd International Conference on Learning Representations (ICLR)* (2015).

[134] Friedman, D. & Dieng, A. B. The Vendi score: a diversity evaluation metric for machine learning. *Trans. Mach. Learn. Res.* **6** (2023).

[135] Pasarkar, A. & Dieng, A. B. Cousins of the Vendi score: a family of similarity-based diversity metrics for science and machine learning. In *Proc. 27th International Conference on Artificial Intelligence & Statistics (AISTATS)* 238 (2024).

[136] Goyal, P. et al. Vision models are more robust and fair when pretrained on uncurated images without supervision. Preprint at arxiv.org/abs/2202.08360 (2022).

[137] Gareth, J., Daniela, W., Trevor, H. & Robert, T. *An Introduction to Statistical Learning: With Applications in R* (Springer, 2013).

[138] Han, J., Pei, J., Yin, Y. & Mao, R. Mining frequent patterns without candidate generation: a frequent-pattern tree approach. *Data Mining Knowl. Discov.* **8**, 53–87 (2004).

[139] Klare, B. F. et al. Pushing the frontiers of unconstrained face detection and recognition: IARPA Janus Benchmark A. In *Proc. 2015 IEEE Conference on Computer Vision and Pattern Recognition (CVPR)* 1931–1939 (IEEE, 2015); 10.1109/CVPR.2015.7298803

[140] Ponce-López, V. et al. Chalearn lap 2016: first round challenge on first impressions-dataset and results. In *Proc. European Conference on Computer Vision* 400–418 (Springer, 2016).

[141] Zhao, A. A. & Zietsch, B. P. Deep neural networks generate facial metrics that overcome limitations of previous methods and predict in-person attraction. *Evol. Hum. Behav.* **45**, 106632 (2024).

[142] Peterson, J. C., Uddenberg, S., Griffiths, T. L., Todorov, A. & Suchow, J. W. Deep models of superficial face judgments. *Proc. Natl Acad. Sci. USA* **119**, 2115228119 (2022). 10.1073/pnas.2115228119 PMC9169911 35446619

[143] Biswas, K., Shivakumara, P., Pal, U., Liu, C.-L. & Lu, Y. Vqapt: A new visual question answering model for personality traits in social media images. *Pattern Recogn. Lett.* **175**, 66–73 (2023).

[144] Andrews, M., Smart, A. & Birhane, A. The reanimation of pseudoscience in machine learning and its ethical repercussions. *Patterns* **5**, 1–14 (2024). 10.1016/j.patter.2024.101027 PMC11573791 39568649

[145] Ruggeri, G. et al. A multi-dimensional study on bias in vision-language models. In *Proc. Findings of the Association for Computational Linguistics* 6445–6455 (ACL, 2023).

[146] Fraser, K. C. & Kiritchenko, S. Examining gender and racial bias in large vision-language models using a novel dataset of parallel images. In *Proc. 18th Conference of the European Chapter of the Association for Computational Linguistics* 1, 690–713 (ACL, 2024).

[147] Huang, J.-t. et al. VisBias: measuring explicit and implicit social biases in vision language models. Preprint at arxiv.org/abs/2503.07575 (2025).

[148] Xiang, A. et al. Code for ‘Fair human-centric image dataset for ethical AI benchmarking’. *Github* https://github.com/SonyResearch/fairness-benchmark-public (2025). 10.1038/s41586-025-09716-2 PMC12675298 41193813

[149] Thomee, B. et al. YFCC100M: the new data in multimedia research. *Commun. ACM* **59**, 64–73 (2016).

[150] Kemelmacher-Shlizerman, I., Seitz, S. M., Miller, D. & Brossard, E. The MegaFace benchmark: 1 million faces for recognition at scale. In *Proc. IEEE Conference on Computer Vision and Pattern Recognition* 4873–4882 (IEEE, 2016).

[151] Cao, Q., Shen, L., Xie, W., Parkhi, O. M. & Zisserman, A. VGGFace2: a dataset for recognising faces across pose and age. In *Proc. 2018 13th IEEE International Conference on Automatic Face & Gesture Recognition (FG 2018)* 67–74 (IEEE, 2018).

[152] Merler, M., Ratha, N., Feris, R. S. & Smith, J. R. Diversity in Faces. Preprint at https://arxiv.org/abs/1901.10436 (2019).

[153] Phillips, P. J. et al. Overview of the face recognition grand challenge. In *Proc*. *2005 IEEE Computer Society Conference on Computer Vision and Pattern Recognition (CVPR’05)* 947–954 (IEEE, 2005); 10.1109/CVPR.2005.268.

[154] Wang, M., Deng, W., Hu, J., Tao, X. & Huang, Y. Racial faces in the wild: reducing racial bias by information maximization adaptation network. In *Proc. IEEE/CVF International Conference on Computer Vision (ICCV)* 692–702 (IEEE, 2019).

[155] Ricanek, K. & Tesafaye, T. Morph: a longitudinal image database of normal adult age-progression. In *Proc. 7th International Conference on Automatic Face and Gesture Recognition (FGR06)* 341–345 (IEEE, 2006).

[156] Eidinger, E., Enbar, R. & Hassner, T. Age and gender estimation of unfiltered faces. *IEEE Trans. Inform. Foren. Secur.* **9**, 2170–2179 (2014).

[157] Wang, M. & Deng, W. Mitigating bias in face recognition using skewness-aware reinforcement learning. In *Proc. IEEE/CVF Conference on Computer Vision and Pattern Recognition (CVPR)* 9322–9331 (IEEE, 2020).

[158] Yang, Y. et al. Enhancing fairness in face detection in computer vision systems by demographic bias mitigation. In *Proc. AAAI/ACM Conference on AI, Ethics, and Society (AIES)* 813–822 (AAAI/ACM, 2022).

[159] Georgopoulos, M., Panagakis, Y. & Pantic, M. Investigating bias in deep face analysis: the KANFace dataset and empirical study. *Image Vis. Comput.* **102**, 103954 (2020).

[160] Karkkainen, K. & Joo, J. FairFace: face attribute dataset for balanced race, gender, and age for bias measurement and mitigation. In *Proc. IEEE Winter Conference on Applications of Computer Vision (WACV)* 1548–1558 (IEEE, 2021).

[161] Russakovsky, O. et al. ImageNet large scale visual recognition challenge. *Int. J. Comput. Vis.* **115**, 211–252 (2015).

[162] Liu, Z., Luo, P., Wang, X. & Tang, X. Deep learning face attributes in the wild. In *Proc. IEEE International Conference on Computer Vision (ICCV)* 3730–3738 (IEEE, 2015).

[163] Zhang, Z., Luo, P., Loy, C.C. & Tang, X. Facial landmark detection by deep multi-task learning. In *Proc. European Conference on Computer Vision (ECCV)* 94–108 (Springer, 2014).

[164] Zhang, Z., Song, Y. & Qi, H. Age progression/regression by conditional adversarial autoencoder. In *Proc. IEEE/CVF Conference on Computer Vision and Pattern Recognition (CVPR)* 5810–5818 (IEEE, 2017).

