# Supplementary material for: Fair human-centric image dataset for ethical AI benchmarking
Source: Nature. 2025 Nov 5;648(8092):97–108. doi: 10.1038/s41586-025-09716-2 (PMC12675298; doi:10.1038/s41586-025-09716-2)
Supplement: Supplementary file 1 — Supplementary Tables, Figures and Supplementary Discussion. [file 41586_2025_9716_MOESM1_ESM.pdf]

nature portfolio

https://doi.org/10.1038/s41586-025-09716-2

Supplementary information

Fair human-centric image dataset for ethical AI benchmarking

In the format provided by the authors and unedited

Supplementary Tables and Figures

A Available Annotations

Self-reported annotations by each subject	
Ancestry	Regional level is a required annotation. Sub-region is optional. Multiple selection allowed from the following [167]:<br>• [0. Africa]: [1. Northern Africa, 2. Eastern Africa, 3. Middle Africa, 4. Southern Africa, 5. Western Africa]<br>• [6. Americas]: [7. Caribbean, 8. Central America, 9. South America, 10. Northern America]<br>• [11. Asia]: [12. Central Asia, 13. Eastern Asia, 14. South-eastern Asia, 15. Southern Asia, 16. Western Asia]<br>• [17. Europe]: [18. Eastern Europe, 19. Northern Europe, 20. Southern Europe, 21. Western Europe]<br>• [22. Oceania]: [23. Australia and New Zealand, 24. Melanesia, 25. Micronesia, 26. Polynesia]
Natural skin tone	Single selection from the following RGB values:<br>• 0. [102, 78, 65]: Dark (Fitzpatrick Type VI)<br>• 1. [136, 105, 81]: Brown (Fitzpatrick Type V)<br>• 2. [164, 131, 103]: Tan (Fitzpatrick Type IV)<br>• 3. [175, 148, 120]: Intermediate (Fitzpatrick Type III)<br>• 4. [189, 163, 137]: Light (Fitzpatrick Type II)<br>• 5. [198, 180, 157]: Very light (Fitzpatrick Type I)
Natural eye color(s)	Multiple selection allowed from the following: [0. None, 1. Blue, 2. Gray, 3. Green, 4. Hazel, 5. Brown, 6. Red and violet, 7. Not listed, 8. Report string], except when option *[0. None]* is selected
Natural head hair type	Single selection from the following:<br>• 0. None<br>• 1. Straight<br>• 2. Wavy<br>• 3. Curly<br>• 4. Kinky-coily<br>• 5. Not listed<br>• 6. Report string
Natural head hair color(s)	Multiple selections allowed from the following: [0. None, 1. Very light blond, 2. Light blond, 3. Blond, 4. Dark blond, 5. Light brown to medium brown, 6. Dark brown/black, 7. Red, 8. Red blond, 9. Gray, 10. White, 11. Not listed, 12. Report string], except when option *[0. None]* is selected
Natural face hair color(s)	Multiple selection allowed from the following: [0. None, 1. Very light blond, 2. Light blond, 3. Blond, 4. Dark blond, 5. Light brown to medium brown, 6. Dark brown/black, 7. Red, 8. Red blond, 9. Gray, 10. White, 11. Not listed, 12. Report string], except when option *[0. None]* is selected
**Self-reported by each subject at capture**	
Age (biological)	Value between 0 and 130
Pronouns	Multiple selection allowed (except when 'None of the above' or 'Prefer not to say' is selected) from the following options: [0. She/her/hers, 1. He/him/his, 2. They/them/theirs, 3. Ze/zir/zirs, 4. None of the above, 5. Prefer not to say]

Continued on next page

Continued from previous page	
Nationality(ies)	Multiple selection allowed from the following options: [0. Afghan, 1. Albanian, 2. Algerian, 3. American, 4. Andorran, 5. Angolan, 6. Anguillan, 7. Citizen of Antigua and Barbuda, 8. Argentine, 9. Armenian, 10. Australian, 11. Austrian, 12. Azerbaijani, 13. Bahamian, 14. Bahraini, 15. Bangladeshi, 16. Barbadian, 17. Belarusian, 18. Belgian, 19. Belizean, 20. Beninese, 21. Bermudian, 22. Bhutanese, 23. Bolivian, 24. Citizen of Bosnia and Herzegovina, 25. Botswanan, 26. Brazilian, 27. British, 28. British Virgin Islander, 29. Bruneian, 30. Bulgarian, 31. Burkinan, 32. Burmese, 33. Burundian, 34. Cambodian, 35. Cameroonian, 36. Canadian, 37. Cape Verdean, 38. Cayman Islander, 39. Central African, 40. Chadian, 41. Chilean, 42. Chinese, 43. Colombian, 44. Comoran, 45. Congolese (Congo), 46. Congolese (DRC), 47. Cook Islander, 48. Costa Rican, 49. Croatian, 50. Cuban, 51. Cymraes, 52. Cymro, 53. Cypriot, 54. Czech, 55. Danish, 56. Djiboutian, 57. Dominican, 58. Citizen of the Dominican Republic, 59. Dutch, 60. East Timorese, 61. Ecuadorean, 62. Egyptian, 63. Emirati, 64. English, 65. Equatorial Guinean, 66. Eritrean, 67. Estonian, 68. Ethiopian, 69. Faroese, 70. Fijian, 71. Filipino, 72. Finnish, 73. French, 74. Gabonese, 75. Gambian, 76. Georgian, 77. German, 78. Ghanaian, 79. Gibraltarian, 80. Greek, 81. Greenlandic, 82. Grenadian, 83. Guamanian, 84. Guatemalan, 85. Citizen of Guinea-Bissau, 86. Guinean, 87. Guyanese, 88. Haitian, 89. Honduran, 90. Hong Konger, 91. Hungarian, 92. Icelandic, 93. Indian, 94. Indonesian, 95. Iranian, 96. Iraqi, 97. Irish, 98. Israeli, 99. Italian, 100. Ivorian, 101. Jamaican, 102. Japanese, 103. Jordanian, 104. Kazakh, 105. Kenyan, 106. Kittitian, 107. Citizen of Kiribati, 108. Kosovan, 109. Kuwaiti, 110. Kyrgyz, 111. Lao, 112. Latvian, 113. Lebanese, 114. Liberian, 115. Libyan, 116. Liechtenstein citizen, 117. Lithuanian, 118. Luxembourger, 119. Macanese, 120. Macedonian, 121. Malagasy, 122. Malawian, 123. Malaysian, 124. Maldivian, 125. Malian, 126. Maltese, 127. Marshallese, 128. Martiniquais, 129. Mauritanian, 130. Mauritian, 131. Mexican, 132. Micronesian, 133. Moldovan, 134. Monegasque, 135. Mongolian, 136. Montenegrin, 137. Montserratian, 138. Moroccan, 139. Mosotho, 140. Mozambican, 141. Namibian, 142. Nauruan, 143. Nepalese, 144. New Zealander, 145. Nicaraguan, 146. Nigerian, 147. Nigerien, 148. Niuean, 149. North Korean, 150. Northern Irish, 151. Norwegian, 152. Omani, 153. Pakistani, 154. Palauan, 155. Palestinian, 156. Panamanian, 157. Papua New Guinean, 158. Paraguayan, 159. Peruvian, 160. Pitcairn Islander, 161. Polish, 162. Portuguese, 163. Prydeinig, 164. Puerto Rican, 165. Qatari, 166. Romanian, 167. Russian, 168. Rwandan, 169. Salvadorean, 170. Sammarinese, 171. Samoan, 172. Sao Tomean, 173. Saudi Arabian, 174. Scottish, 175. Senegalese, 176. Serbian, 177. Citizen of Seychelles, 178. Sierra Leonean, 179. Singaporean, 180. Slovak, 181. Slovenian, 182. Solomon Islander, 183. Somali, 184. South African, 185. South Korean, 186. South Sudanese, 187. Spanish, 188. Sri Lankan, 189. St Helenian, 190. St Lucian, 191. Stateless, 192. Sudanese, 193. Surinamese, 194. Swazi, 195. Swedish, 196. Swiss, 197. Syrian, 198. Taiwanese, 199. Tajik, 200. Tanzanian, 201. Thai, 202. Togolese, 203. Tongan, 204. Trinidadian, 205. Tristanian, 206. Tunisian, 207. Turkish, 208. Turkmen, 209. Turks and Caicos Islander, 210. Tuvaluan, 211. Ugandan, 212. Ukrainian, 213. Uruguayan, 214. Uzbek, 215. Vatican citizen, 216. Citizen of Vanuatu, 217. Venezuelan, 218. Vietnamese, 219. Vincentian, 220. Wallisian, 221. Welsh, 222. Yemeni, 223. Zambian, 224. Zimbabwean, 225. Not listed, 226. Report string]. The list of nationalities is obtained from [168].
Continued on next page	

Continued from previous page

Country/territory of residence	Multiple selection allowed from the following: [0. Afghanistan, 1. Aland Islands, 2. Albania, 3. Alderney, 4. Algeria, 5. American Samoa, 6. Andorra, 7. Angola, 8. Anguilla, 9. Antarctica, 10. Antigua and Barbuda, 11. Argentina, 12. Armenia, 13. Aruba, 14. Australia, 15. Austria, 16. Azerbaijan, 17. Bahamas, 18. Bahrain, 19. Bangladesh, 20. Barbados, 21. Belarus, 22. Belgium, 23. Belize, 24. Benin, 25. Bermuda, 26. Bhutan, 27. Bolivia, 28. Bonaire, Sint Eustatius and Saba, 29. Bosnia and Herzegovina, 30. Botswana, 31. Bouvet Island, 32. Brazil, 33. British Indian Ocean Territory, 34. Brunei Darussalam, 35. Bulgaria, 36. Burkina Faso, 37. Burundi, 38. Cambodia, 39. Cameroon, 40. Canada, 41. Cape Verde, 42. Cayman Islands, 43. Central African Republic, 44. Chad, 45. Chile, 46. China, 47. Christmas Island, 48. Cocos (Keeling) Islands, 49. Colombia, 50. Comoros, 51. Congo, 52. Congo, the Democratic Republic of the, 53. Cook Islands, 54. Costa Rica, 55. Croatia, 56. Cuba, 57. Curacao, 58. Cyprus, 59. Czech Republic, 60. Denmark, 61. Djibouti, 62. Dominica, 63. Dominican Republic, 64. East Timor, 65. Ecuador, 66. Egypt, 67. El Salvador, 68. England, 69. Equatorial Guinea, 70. Eritrea, 71. Estonia, 72. Eswatini, 73. Ethiopia, 74. Falkland Islands, 75. Faroe Islands, 76. Fiji, 77. Finland, 78. France, 79. French Guiana, 80. French Polynesia, 81. French Southern Territories, 82. Gabon, 83. Gambia, 84. Georgia, 85. Germany, 86. Ghana, 87. Gibraltar, 88. Greece, 89. Greenland, 90. Grenada, 91. Guadeloupe, 92. Guam, 93. Guatemala, 94. Guernsey, 95. Guinea, 96. Guinea-Bissau, 97. Guyana, 98. Haiti, 99. Heard Island and McDonald Islands, 100. Herm, 101. Honduras, 102. Hong Kong, 103. Hungary, 104. Iceland, 105. India, 106. Indonesia, 107. Iran, 108. Iraq, 109. Ireland, 110. Isle of Man, 111. Israel, 112. Italy, 113. Ivory Coast, 114. Jamaica, 115. Japan, 116. Jersey, 117. Jordan, 118. Kazakhstan, 119. Kenya, 120. Kiribati, 121. Kosovo, 122. Kuwait, 123. Kyrgyzstan, 124. Laos, 125. Latvia, 126. Lebanon, 127. Lesotho, 128. Liberia, 129. Libya, 130. Liechtenstein, 131. Lithuania, 132. Luxembourg, 133. Macao, 134. Macedonia, 135. Madagascar, 136. Malawi, 137. Malaysia, 138. Maldives, 139. Mali, 140. Malta, 141. Marshall Islands, 142. Martinique, 143. Mauritania, 144. Mauritius, 145. Mayotte, 146. Mexico, 147. Micronesia, 148. Moldova, 149. Monaco, 150. Mongolia, 151. Montenegro, 152. Montserrat, 153. Morocco, 154. Mozambique, 155. Myanmar, 156. Namibia, 157. Nauru, 158. Nepal, 159. Netherlands, 160. New Caledonia, 161. New Zealand, 162. Nicaragua, 163. Niger, 164. Nigeria, 165. Niue, 166. Norfolk Island, 167. North Korea, 168. Northern Ireland, 169. Northern Mariana Islands, 170. Norway, 171. Oman, 172. Pakistan, 173. Palau, 174. Palestine, State of, 175. Panama, 176. Papua New Guinea, 177. Paraguay, 178. Peru, 179. Philippines, 180. Pitcairn, 181. Poland, 182. Portugal, 183. Puerto Rico, 184. Qatar, 185. Reunion, 186. Romania, 187. Russia, 188. Rwanda, 189. Saint Barthelemy, 190. Saint Helena, Ascension and Tristan da Cunha, 191. Saint Kitts and Nevis, 192. Saint Lucia, 193. Saint Martin (French part), 194. Saint Pierre and Miquelon, 195. Saint Vincent and the Grenadines, 196. Samoa, 197. San Marino, 198. Sao Tome and Principe, 199. Sark, 200. Saudi Arabia, 201. Scotland, 202. Senegal, 203. Serbia, 204. Seychelles, 205. Sierra Leone, 206. Singapore, 207. Sint Maarten (Dutch part), 208. Slovakia, 209. Slovenia, 210. Solomon Islands, 211. Somalia, 212. South Africa, 213. South Georgia and the South Sandwich Islands, 214. South Korea, 215. South Sudan, 216. Spain, 217. Sri Lanka, 218. Sudan, 219. Suriname, 220. Svalbard and Jan Mayen, 221. Sweden, 222. Switzerland, 223. Syria, 224. Taiwan, 225. Tajikistan, 226. Tanzania, 227. Thailand, 228. Togo, 229. Tokelau, 230. Tonga, 231. Trinidad and Tobago, 232. Tunisia, 233. Turkey, 234. Turkmenistan, 235. Turks and Caicos Islands, 236. Tuvalu, 237. Uganda, 238. Ukraine, 239. United Arab Emirates, 240. United Kingdom, 241. United States, 242. United States Minor Outlying Islands, 243. Uruguay, 244. Uzbekistan, 245. Vanuatu, 246. Vatican City, 247. Venezuela, 248. Vietnam, 249. Virgin Islands, British, 250. Virgin Islands, U.S., 251. Wales, 252. Wallis and Futuna, 253. Western Sahara, 254. Yemen, 255. Zambia, 256. Zimbabwe, 257. Not listed, 258. Report string]. The list of geographical names is obtained from [169]. Only released as aggregate statistic or upon request.
Disability(ies) (optional)	Multiple selection allowed (except when 'None of the above' or 'Prefer not to say' is selected) from the following: [0. Hearing difficulty, 1. Vision difficulty, 2. Cognitive difficulty, 3. Ambulatory difficulty, 4. Self-care difficulty, 5. Independent living difficulty, 6. None of the above, 7. Prefer not to say]. Only released as aggregate statistic.
Height	Value in centimeters (e.g., 185 cm). Only released as aggregate statistic.
Weight	Value in kilograms (kgs). (e.g., 60 kgs). Only released as aggregate statistic.
Pregnancy status (optional)	Single selection from the following options:<br>• 0. Not pregnant<br>• 1. Pregnant<br>• 2. Visibly pregnant<br>• 3. Not visibly pregnant<br>• 4. Prefer not to say<br>Only released as aggregate statistic.
Biologically related image subjects	Binary value: True or False. Only released as aggregate statistic.

Continued on next page

Continued from previous page

Body pose	Single selection from the following options:<br>• 0. Standing<br>• 1. Sitting<br>• 2. Walking<br>• 3. Bending/bowing<br>• 4. Lying down/sleeping<br>• 5. Performing martial/fighting arts<br>• 6. Dancing<br>• 7. Running/jogging<br>• 8. Crouching/kneeling<br>• 9. Getting up<br>• 10. Jumping/leaping<br>• 11. Falling down<br>• 12. Crawling<br>• 13. Swimming<br>• 14. Not listed<br>• 15. Report string
Apparent skin tone	Single selection allowed from the following RGB values (vendors were instructed to provide visual representations of these RGB values to subjects):<br>• 0. [102, 78, 65]: Dark (Fitzpatrick Type VI)<br>• 1. [136, 105, 81]: Brown (Fitzpatrick Type V)<br>• 2. [164, 131, 103]: Tan (Fitzpatrick Type IV)<br>• 3. [175, 148, 120]: Intermediate (Fitzpatrick Type III)<br>• 4. [189, 163, 137]: Light (Fitzpatrick Type II)<br>• 5. [198, 180, 157]: Very light (Fitzpatrick Type I)
Apparent eye color(s)	Multiple selections allowed from the following: [0. None, 1. Blue, 2. Gray, 3. Green, 4. Hazel, 5. Brown, 6. Red and violet, 7. Not listed, 8. Report string], except when option *[0. None]* is selected
Apparent head hair type	Single selection from the following:<br>• 0. None<br>• 1. Straight<br>• 2. Wavy<br>• 3. Curly<br>• 4. Kinky-coily<br>• 5. Not listed<br>• 6. Report string
Head hairstyle	Single selection from the following:<br>• 0. None<br>• 1. Buzz cut<br>• 2. Short<br>• 3. Up (Short)<br>• 4. Half-up (Short)<br>• 5. Down (Short)<br>• 6. Not listed (Short)<br>• 7. Medium<br>• 8. Up (Medium)<br>• 9. Half-up (Medium)<br>• 10. Down (Medium)<br>• 11. Not listed (Medium)<br>• 12. Long<br>• 13. Up (Long)<br>• 14. Half-up (Long)<br>• 15. Down (Long)<br>• 16. Not listed (Long)<br>• 17. Not listed<br>• 18. Report string
Apparent head hair color(s)	Multiple selection allowed from the following: [0. None, 1. Very light blond, 2. Light blond, 3. Blond, 4. Dark blond, 5. Light brown to medium brown, 6. Dark brown/black, 7. Red, 8. Red blond, 9. Gray, 10. White, 11. Not listed, 12. Report string], except when option *[0. None]* is selected.
Facial hairstyle	Multiple selection allowed from the following: [0. None, 1. Beard, 2. Mustache, 3. Goatee]
Apparent facial hair color(s)	Multiple selection allowed from the following: [0. None, 1. Very light blond, 2. Light blond, 3. Blond, 4. Dark blond, 5. Light brown to medium brown, 6. Dark brown/black, 7. Red, 8. Red blond, 9. Gray, 10. White, 11. Not listed, 12. Report string], except when option *[0. None]* is selected.

Continued on next page

Continued from previous page	
Facial marks	Multiple selection allowed from the following: [0. None, 1. Tattoos, 2. Birthmarks, 3. Scars, 4. Burns, 5. Growths, 6. Make-up, 7. Face paint, 8. Acne 9. Not listed, 10. Report string]
Subject-object interaction(s)	Multiple selection allowed from the following: [0. None, 1. Riding, 2. Driving, 3. Watching, 4. Smoking, 5. Eating, 6. Drinking, 7. Opening or closing, 8. Lifting/picking up or putting down, 9. Writing/drawing or painting, 10. Catching or throwing, 11. Pushing, pulling or extracting, 12. Putting on or taking off clothing, 13. Entering or exiting, 14. Climbing, 15. Pointing at, 16. Shooting at, 17. Digging/shoveling, 18. Playing with pets/animals, 19. Playing musical instrument, 20. Playing, 21. Using an electronic device, 22. Cutting or chopping, 23. Cooking, 24. Fishing, 25. Rowing, 26. Sailing, 27. Brushing teeth, 28. Hitting, 29. Kicking, 30. Turning, 31. Not listed, 32. Report string]
Subject-subject interaction(s)	Multiple selection allowed from the following: [0. Not applicable, 1. Talking/listening/singing, 2. Watching/looking, 3. Grabbing, 4. Hitting, 5. Kicking, 6. Pushing, 7. Hugging/embracing, 8. Giving/serving or taking/receiving, 9. Kissing, 10. Lifting, 11. Hand shaking, 12. Playing with, 13. Not listed, 14. Report string]
**Reported by primary subject at capture**	
Weather	Multiple selection allowed from the following: [0. Fog, 1. Haze, 2. Snow/hail, 3. Rain, 4. Humid, 5. Cloud, 6. Clear]
Camera position	Single selection from the following:<br>• 0. Typical: Camera was at the primary subject's eye line.<br>• 1. Atypical High: Camera was above the primary subject's eye line.<br>• 2. Atypical Low: Camera was below the primary subject's eye line.
Illumination	Multiple selection allowed from the following: [0. Lighting from above the head/face, 1. Lighting from below the head/face, 2. Lighting from in front of the head/face, 3. Lighting from behind the head/face, 4. Lighting from the left of the head/face, 5. Lighting from the right of the head/face]
Image capture scene	Single selection from the following:<br>• 0. Outdoor: Water, ice, snow<br>• 1. Outdoor: Mountains, hills, desert, sky<br>• 2. Outdoor: Forest, field, jungle<br>• 3. Outdoor: Man-made elements<br>• 4. Outdoor: Transportation<br>• 5. Outdoor: Cultural or historical building/place<br>• 6. Outdoor: Sports fields, parks, leisure spaces<br>• 7. Outdoor: Industrial and construction<br>• 8. Outdoor: Houses, cabins, gardens, and farms<br>• 9. Outdoor: Commercial buildings, shops, markets, cities, and towns<br>• 10. Indoor: Shopping and dining<br>• 11. Indoor: Workplace<br>• 12. Indoor: Home or hotel<br>• 13. Indoor: Transportation<br>• 14. Indoor: Sports and leisure<br>• 15. Indoor: Cultural
Image capture date	Date of capture in Month-YYYY format (e.g., February-2021).
Image capture time window	Approximate time of capture in six-hour time window in 24-hour format (e.g., 00:00-05:59).
Image capture place	Region of capture and country of capture.
Subject position	Selection from the following options:<br>• 0. Above: Primary subject is above secondary subject in the image.<br>• 1. Below: Primary subject is below secondary subject in the image.<br>• 2. Right: Primary subject is to the right of secondary subject in the image.<br>• 3. Left: Primary subject is to the left of secondary subject in the image.<br>• 4. None: Primary subject only.
**Obtained from human annotators**	
Head pose	Single selection from the following:<br>• 0. Typical: The absolute pitch is smaller than 30° and the absolute yaw is less than 30°.<br>• 1. Atypical: The absolute pitch is larger than 30° and/or the absolute yaw is larger than 30°.
Continued on next page	

Continued from previous page	
Segmentation masks	Masks are encoded as polygons with x and y coordinates for the vertices of the polygon (as in MS-COCO) for the following: `[0. Face skin, 1. Upper body skin, 2. Left arm skin, 3. Right arm skin, 4. Left leg skin, 5. Right leg skin, 6. Head hair, 7. Left eyebrow, 8. Right eyebrow, 9. Left eye, 10. Right eye, 11. Nose, 12. Upper lip, 13. Lower lip, 14. Inner mouth, 15. Left shoe, 16. Right shoe, 17. Headwear, 18. Mask, 19. Eyewear, 20. Upper body clothes, 21. Lower body clothes, 22. Full body clothes, 23. Sock or legwarmer, 24. Neckwear, 25. Bag, 26. Glove, 27. Jewelry or timepiece]`
Facial bounding box	$[x, y, width, height]$, where `x` and `y` are the upper-left coordinates and `width` and `height` are the dimensions of the bounding box.
Camera Distance	Derived from the face bounding box. Single selection from the following:<br>• `0. CD I`: Face height is between 10–49 pixels.<br>• `1. CD II`: Face height is between 50–299 pixels.<br>• `2. CD III`: Face height is between 300–899 pixels.<br>• `3. CD IV`: Face height is between 900–1499 pixels.<br>• `4. CD V`: Face height is 1500+ pixels.
Keypoints	$[x, y, visibility]$ for every keypoint where x and y are the keypoint coordinates and *visibility* is a binary value (0 if not visible, 1 if visible) (as in MS-COCO) for the following keypoints: `[0. Nose, 1. Right eye inner, 2. Right eye, 3. Right eye outer, 4. Left eye inner, 5. Left eye, 6. Left eye outer, 7. Right ear, 8. Left ear, 9. Mouth right, 10. Mouth left, 11. Right shoulder, 12. Left shoulder, 13. Right elbow, 14. Left elbow, 15. Right wrist, 16. Left wrist, 17. Right pinky knuckle, 18. Left pinky knuckle, 19. Right index knuckle, 20. Left index knuckle, 21. Right thumb knuckle, 22. Left thumb knuckle, 23. Right hip, 24. Left hip, 25. Right knee, 26. Left knee, 27. Right ankle, 28. Left ankle, 29. Right heel, 30. Left heel, 31. Right foot index, 32. Left foot index]`
Non-consensual person segmentation mask	Required if a non-consensual subject exists and can be annotated with a segmentation mask. Masks are encoded as polygons with x and y coordinates for the vertices of the polygon (as in MS-COCO) with class name: `28. Non-consensual person`. The field is an empty list if a non-consensual subject, or a crowd, is annotated with a bounding box. This annotation will not be released.
Non-consensual person bounding box	Required if a non-consensual subject exists and can be annotated with a bounding box. The bounding box is in $[x, y, width, height]$ format, where `x` and `y` are the upper-left coordinates and `width` and `height` are the dimensions of the bounding box. The field is an empty list if a non-consensual subject, or a crowd, is not allowed to be annotated with a bounding box. This annotation will not be released.

Supplementary Table 1: List of all of FHIBE's available attribute annotations and associated values for each attribute.

B FHIBE Summary Statistics

B.1 FHIBE Distribution

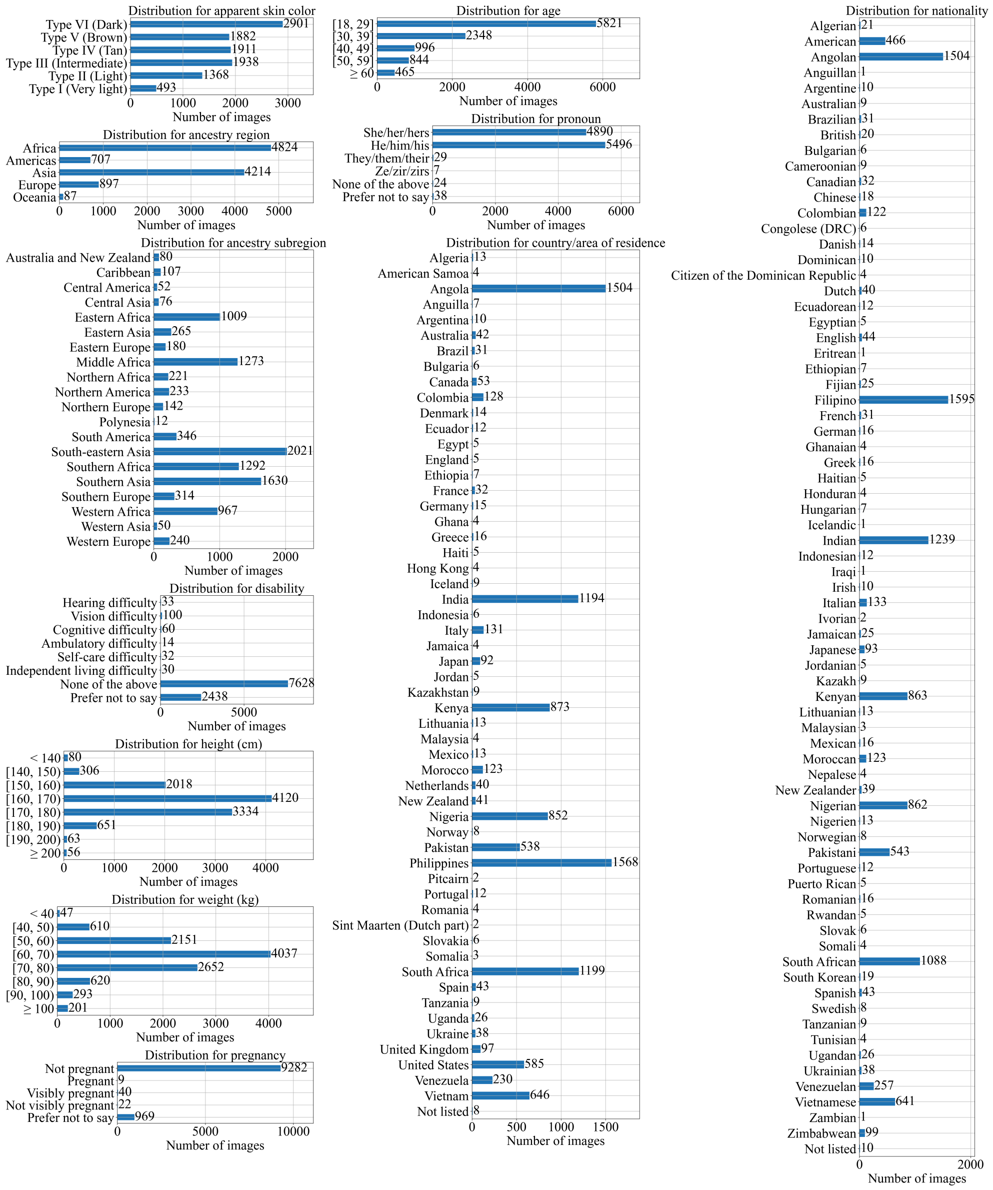

Supplementary Figure 1: Distribution of images associated with key attributes in FHIBE. This figure depicts the distribution of images corresponding to key attributes within the FHIBE dataset. Some images may have multiple annotated labels for specific attributes, resulting in variations in the total sample count across attributes. In compliance with the IRB protocol, certain sensitive attributes are not publicly released, as outlined in Appendix A. For transparency, the aggregated distribution of key sensitive attributes is presented. For self-reported weight and height values, a few extreme outliers are observed; however, these do not significantly influence the overall distribution. The distributions by subject are shown in the Extended Data section of the manuscript.

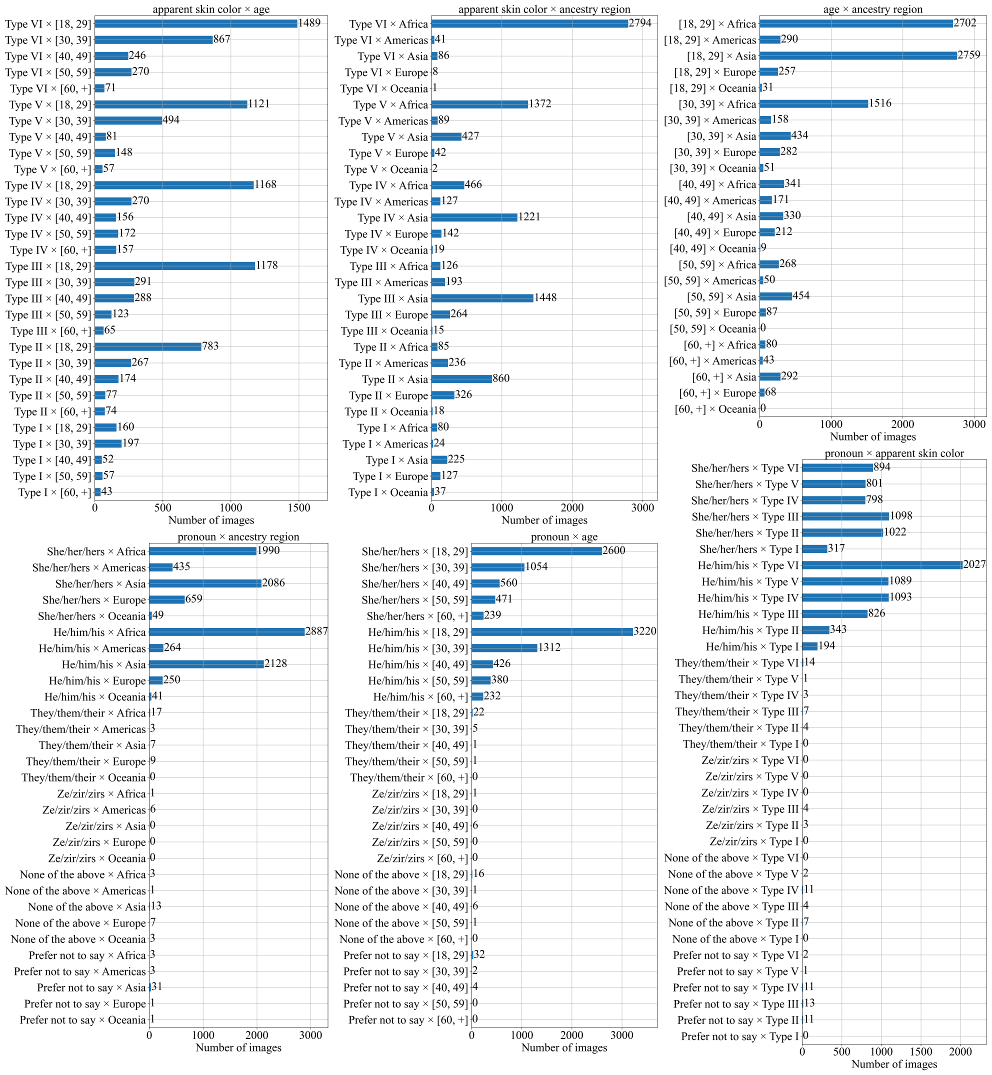

Supplementary Figure 2: **Intersectional distribution of FHIBE key attributes based on image counts.** This figure illustrates the intersectional distribution of key attributes in the FHIBE dataset, measured by the number of images. Some images may have multiple annotated labels for specific attributes, resulting in variations in the total sample count across attributes.

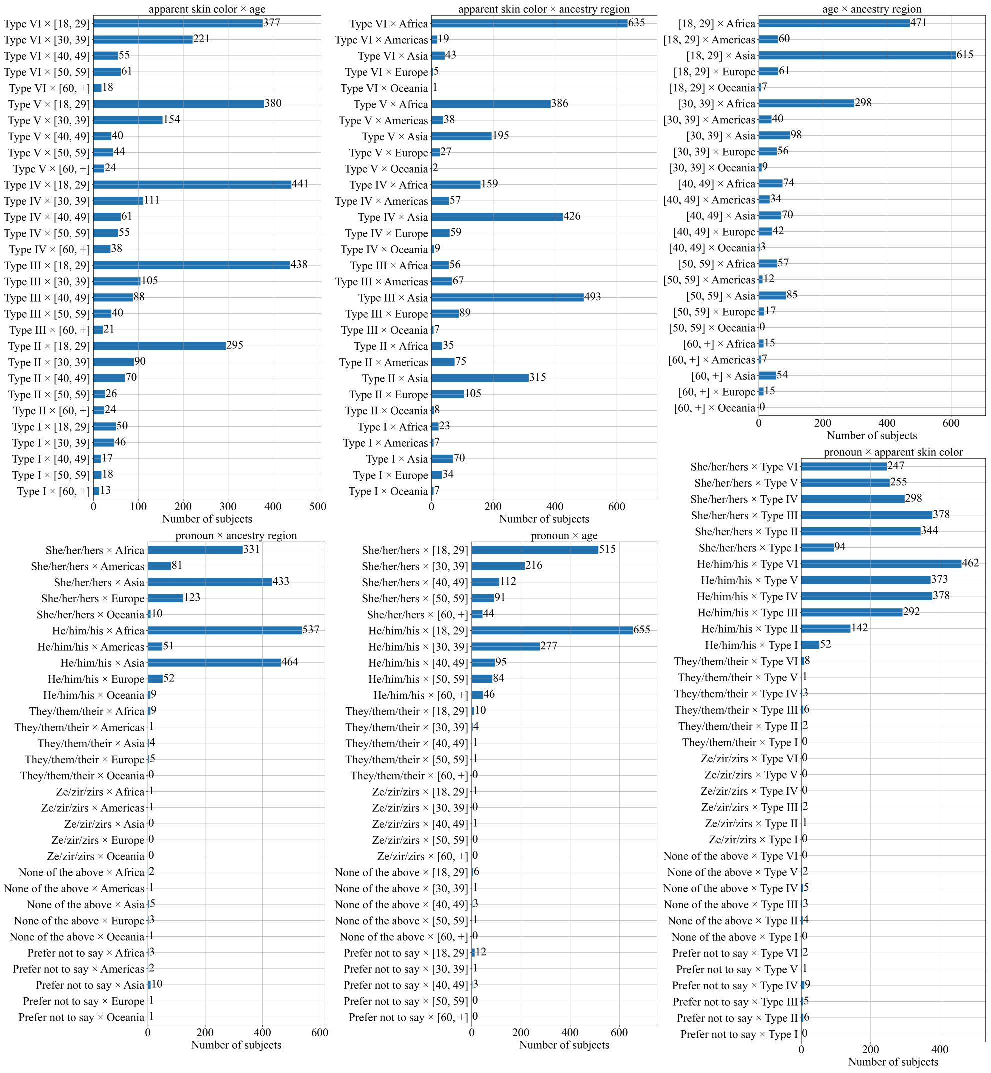

Supplementary Figure 3: **Intersectional distribution of FHIBE key attributes based on subject counts.** This figure illustrates the intersectional distribution of key attributes in the FHIBE dataset, measured by the number of subjects. Some subjects may have multiple annotated labels for specific attributes, resulting in variations in the total sample count across attributes.

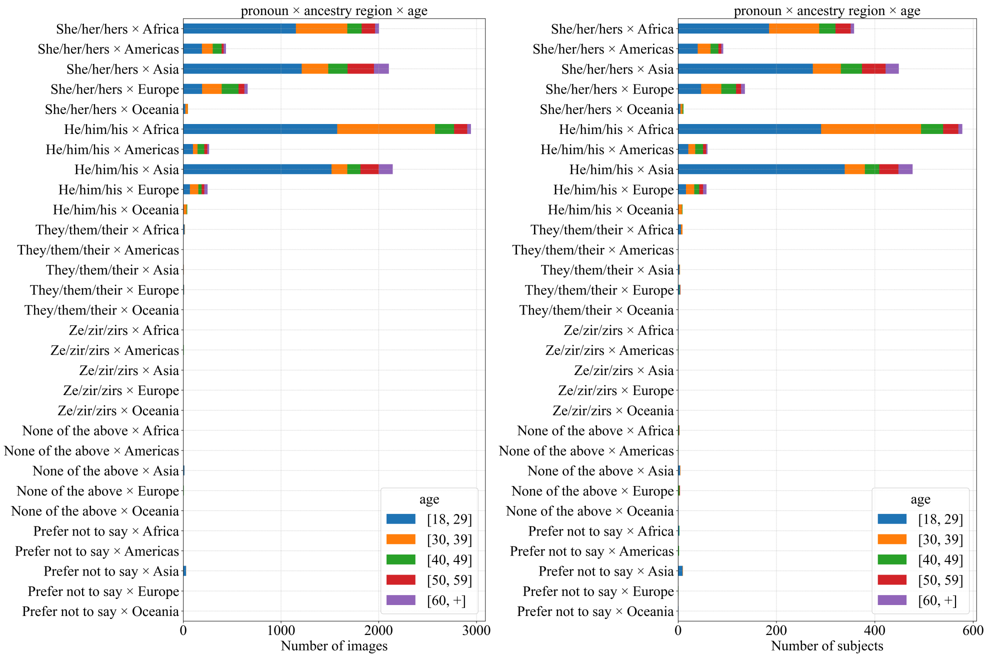

Supplementary Figure 4: Intersectional distribution of FHIBE key attributes. This figure illustrates the intersectional distribution of key attributes in the FHIBE dataset. Some samples may have multiple annotated labels for specific attributes, resulting in variations in the total sample count across attributes.

B.2 FHIBE vs. Other Datasets

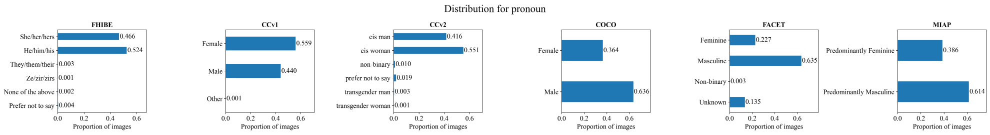

(a) Proportional distribution of images for *pronoun*.

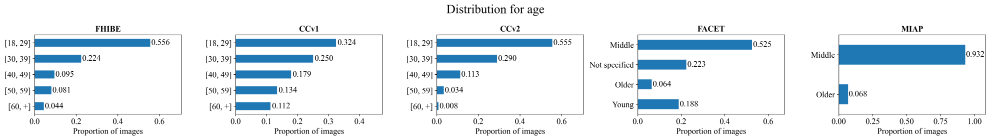

(b) Proportional distribution of images for *age*.

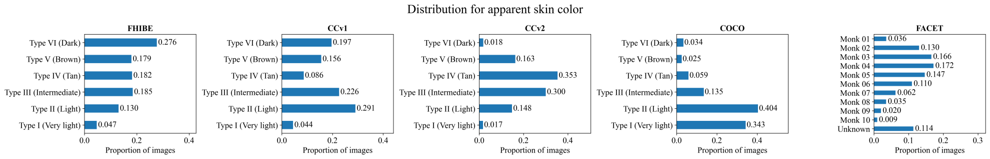

(c) Proportional distribution of images for *apparent skin color*.

Supplementary Figure 5: **Proportional distribution of images for pronoun, age, and apparent skin color across FHIBE and other datasets.** This figure compares the proportional distribution of images for pronoun, age, and apparent skin color attributes in FHIBE and other datasets used in this paper. Original attribute labels are preserved. Datasets lacking a specific attribute are excluded from the corresponding subfigure. Note that some images may have multiple annotated labels for specific attributes, resulting in variations in the total sample count across attributes. The comparative distributions by subject are shown in the Extended Data section of the manuscript.

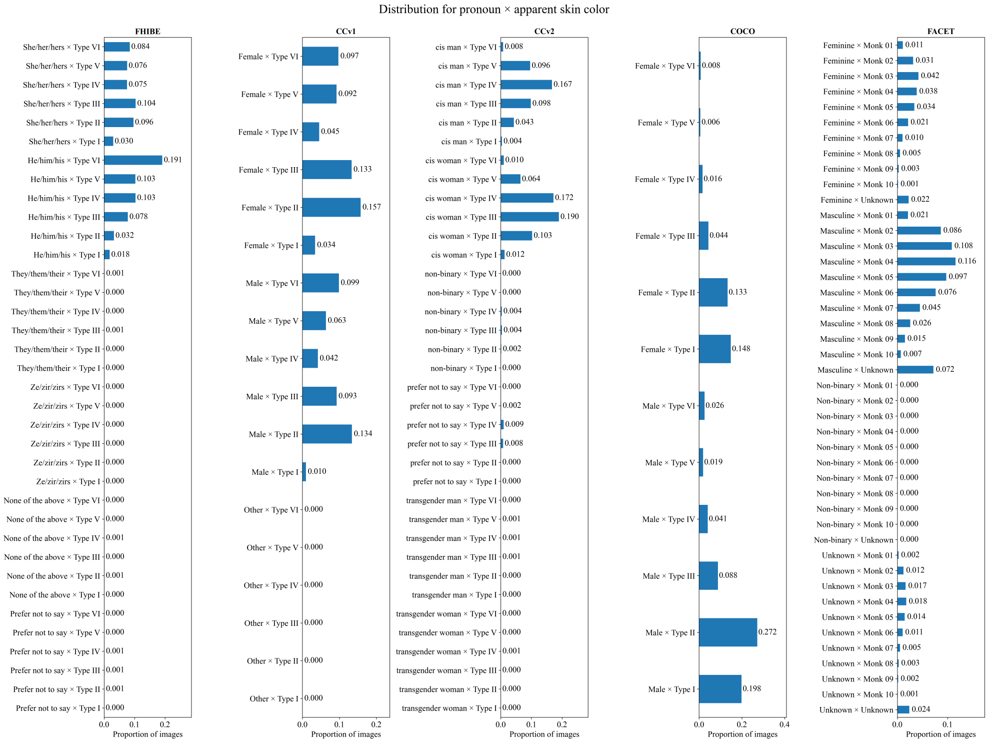

(a) Intersectional proportional distribution of images for *pronoun* × *apparent skin color*.

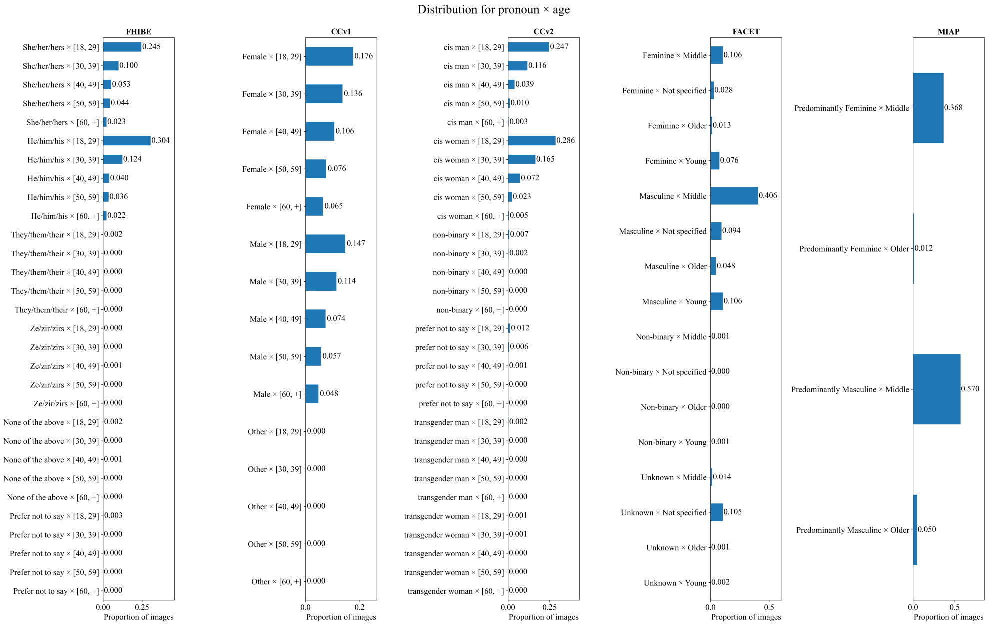

(b) Intersectional proportional distribution of images for *pronoun* × *age*.

Supplementary Figure 6: **Intersectional proportional distribution of images across FHIBE and other datasets.** This figure shows the intersectional proportional distribution of images across FHIBE and other datasets used in this paper. Original attribute labels are preserved. Datasets lacking a specific attribute are excluded from the corresponding subfigure. Note that some samples may have multiple annotated labels for specific attributes, resulting in variations in the total sample count across attributes.

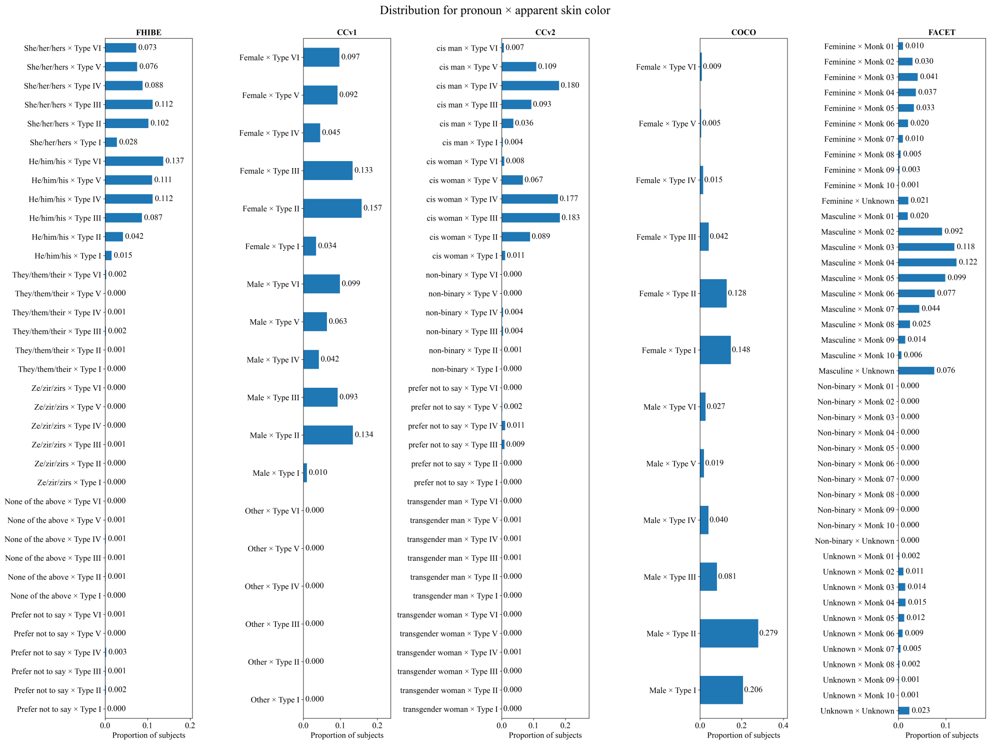

(a) Intersectional proportional distribution of subjects for *pronoun* × *apparent skin color*.

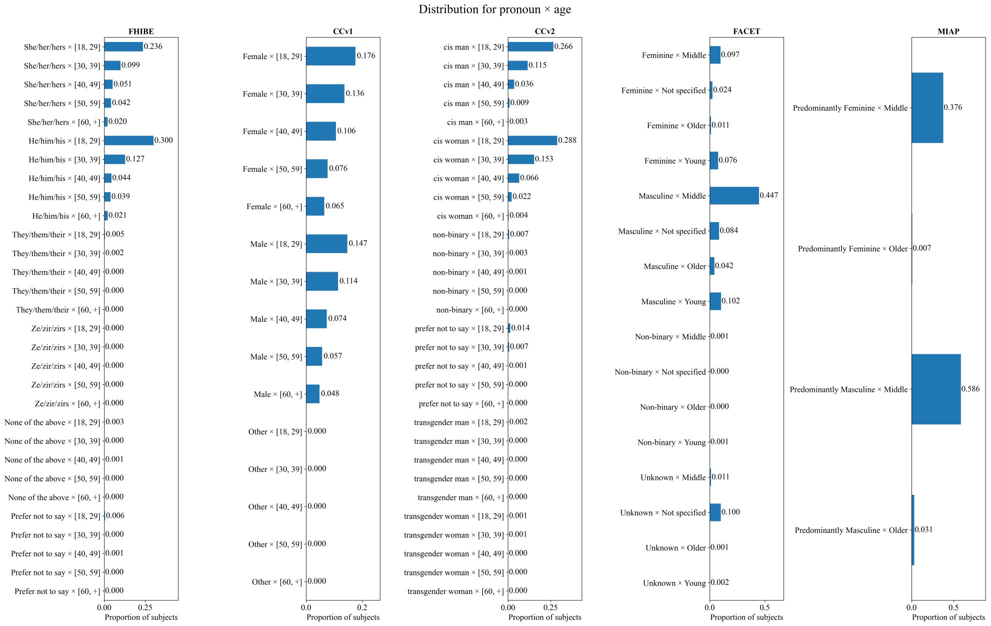

(b) Intersectional proportional distribution of subjects for *pronoun* × *age*.

Supplementary Figure 7: **Intersectional proportional distribution of subjects across FHIBE and other datasets.** This figure illustrates the intersectional proportional distribution of subjects across FHIBE and other datasets used in this paper. Original attribute labels are preserved. Datasets lacking a specific attribute are excluded from the corresponding subfigure. Note that some subjects may have multiple annotated labels for specific attributes, resulting in variations in the total sample count across attributes.

C Participants Involved in Data Collection, Annotation, and Quality Assurance

Participant category	Definition
Data vendor	An entity that acquired and processed data on our behalf for the project.
Data subject	An individual who contributed their personal data to the project.
Image subject	A crowdsourced data subject who consented to their images, corresponding metadata, and self-reported attributes being included in the dataset.
Primary image subject	The consensual image subject who directly submitted their images to a data vendor.
Secondary image subject	The consensual image subject featured alongside a primary image subject in an image provided to a data vendor.
Annotator	An individual who annotated the collected images. Some annotators were data vendor employees, while others were crowdsourced workers on the data vendor's platform. An annotator who voluntarily contributed self-reported attributes is also a data subject.
QA annotator	A data vendor employee who conducted quality assurance work on image annotations. A QA annotator who voluntarily contributed self-reported attributes is also a data subject.
QA specialist	Contractors we hired to review images, metadata, and annotations. A QA specialist who voluntarily contributed self-reported attributes is also a data subject.
Non-consensual subject	A subject who is incidentally featured in images (e.g., in the background) provided to a data vendor without their explicit consent. These individuals were removed from FHIBE.
IP rights holder	An individual who holds the copyright to an image provided to a data vendor.

Supplementary Table 2: Definitions of the different categories of participants involved in data collection, annotation, and QA in the context of FHIBE.

D Annotator and QA Annotator Demographics

In the following section we show some visualizations highlighting our annotator and QA Annotator demographics, to highlight the diversity of the annotators and QA annotators. Annotators added the non-self-reported annotations (pixel-level annotations, head pose, and camera distance). QA annotators were responsible for checking annotations.

D.1 Annotators

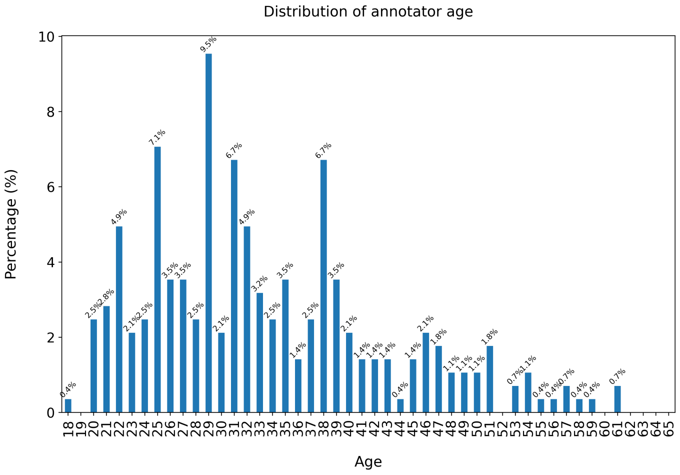

Supplementary Figure 8: **Distribution of annotator age values.** This histogram illustrates the distribution of annotator ages. 50 out of the 333 annotators opted not to self-report their demographics and are excluded from this figure.

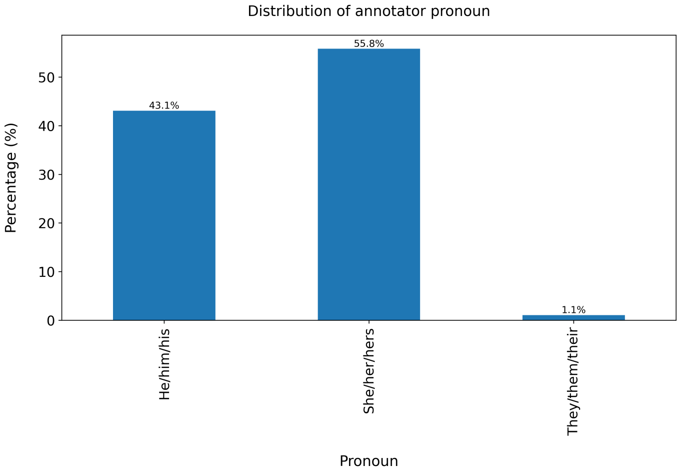

Supplementary Figure 9: **Distribution of annotator pronoun values.** This histogram illustrates the distribution of annotator pronouns. 50 out of the 333 annotators opted not to self-report their demographics and are excluded from this figure.

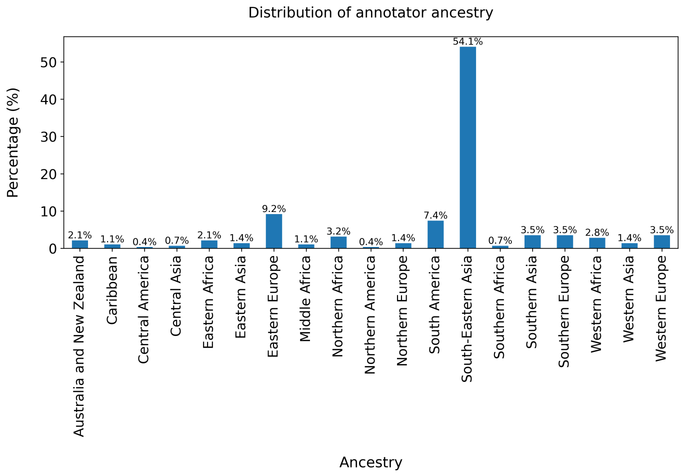

Supplementary Figure 10: **Distribution of annotator ancestry values.** This histogram illustrates the distribution of annotator ancestry values. 50 out of the 333 annotators opted not to self-report their demographics and are excluded from this figure.

D.2 QA Annotators

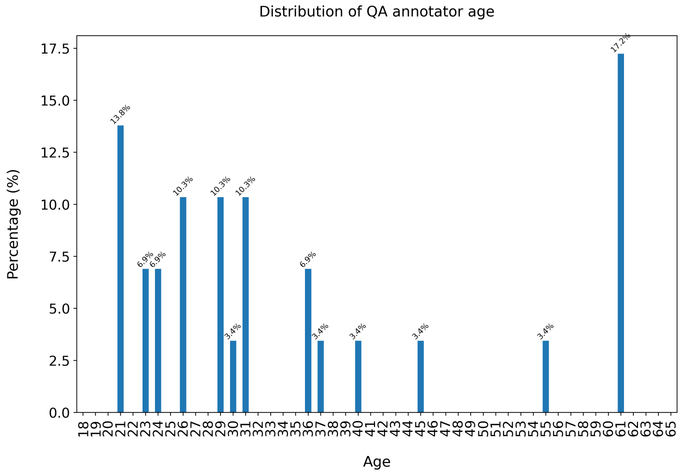

Supplementary Figure 11: **Distribution of QA annotator age values.** This histogram illustrates the distribution of QA annotator ages. 50 out of the 79 QA annotators opted not to self-report their demographics and are excluded from this figure.

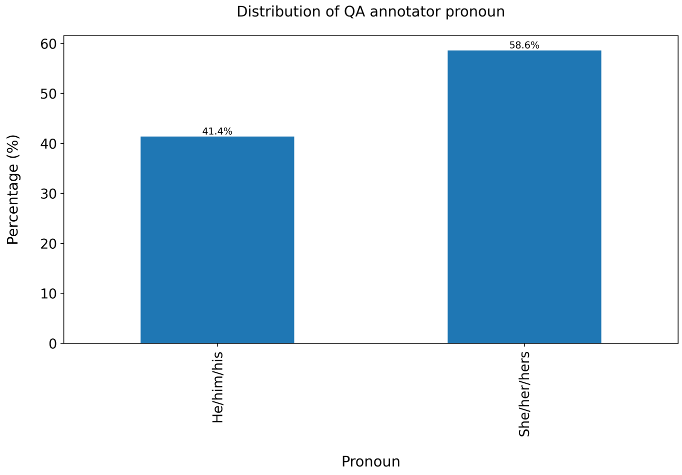

Supplementary Figure 12: **Distribution of QA annotator pronoun values.** This histogram illustrates the distribution of QA annotator pronouns. 50 out of the 79 QA annotators opted not to self-report their demographics and are excluded from this figure.

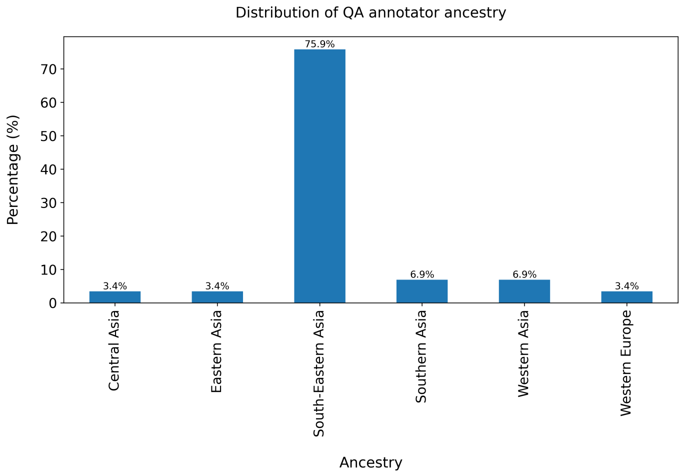

Supplementary Figure 13: **Distribution of QA annotator ancestry values.** This histogram illustrates the distribution of QA annotator ancestry values. 50 out of the 79 QA annotators opted not to self-report their demographics and are excluded from this figure.

E Inter-Rater Reliability

Feature type	Metric	Vendor 1<br>score (samples)	Vendor 2<br>score (samples)
Landmark annotations (keypoints)	Object Keypoint Similarity	0.98 (320)	0.96 (180)
Segmentation masks	Sørensen-Dice Coefficient	0.97 (317)	0.91 (179)
Facial bounding box	mean Intersection over Union	0.98 (313)	0.98 (187)
Body pose	Jaccard Similarity Coefficient	0.80 (287)	0.92 (213)
Apparent skin tone	Jaccard Similarity Coefficient	0.53 (287)	0.48 (213)
Apparent left eye color	Jaccard Similarity Coefficient	0.84 (285)	0.77 (215)
Apparent right eye color	Jaccard Similarity Coefficient	0.84 (279)	0.76 (221)
Apparent head hair type	Jaccard Similarity Coefficient	0.81 (296)	0.84 (204)
Head hairstyle	Jaccard Similarity Coefficient	0.77 (290)	0.83 (210)
Apparent head hair color(s)	Jaccard Similarity Coefficient	0.76 (274)	0.87 (226)
Facial hairstyle	Jaccard Similarity Coefficient	0.94 (277)	0.96 (223)
Apparent face hair color(s)	Jaccard Similarity Coefficient	0.91 (290)	0.95 (210)
Facial marks	Jaccard Similarity Coefficient	0.78 (280)	0.80 (220)
Subject-object interaction(s)	Jaccard Similarity Coefficient	0.86 (281)	0.68 (219)
Weather	Jaccard Similarity Coefficient	0.94 (293)	0.86 (207)
Image capture scene	Jaccard Similarity Coefficient	0.77 (292)	0.85 (208)
Head pose	Jaccard Similarity Coefficient	0.81 (291)	0.84 (209)

Supplementary Table 3: **Comparison of vendor collected annotations with internal expert annotations.** This table compares the quality of annotations collected by two vendors against internal expert annotations across various feature types. Metrics include Object Keypoint Similarity [170] for landmark annotations, Sørensen-Dice Coefficient [171, 172] for segmentation masks, mean Intersection over Union for facial bounding box, and Jaccard Similarity Coefficient [173] for categorical attributes. All these metrics range from 0 to 1, where higher values indicate greater agreement. The sample sizes used for each comparison are shown in parentheses.

Feature type	Metric	Vendor 1	Vendor 2	Between Vendors
Landmark annotations (keypoints)	Object Keypoint Similarity	0.93	0.93	0.95
Segmentation masks	Sørensen-Dice Coefficient	0.90	0.93	0.91
Facial bounding box	mean Intersection over Union	0.92	0.95	0.93
Ancestry	Jaccard Similarity Coefficient	1.00	0.98	0.99
Natural skin tone	Jaccard Similarity Coefficient	1.00	0.72	0.85
Natural left eye color	Jaccard Similarity Coefficient	1.00	0.85	0.90
Natural right eye color	Jaccard Similarity Coefficient	1.00	0.85	0.90
Natural head hair type	Jaccard Similarity Coefficient	1.00	0.72	0.84
Natural head hair color(s)	Jaccard Similarity Coefficient	1.00	0.85	0.91
Natural face hair color(s)	Jaccard Similarity Coefficient	1.00	1.00	0.99
Age (biological)	Jaccard Similarity Coefficient	1.00	0.98	0.99
Pronoun	Jaccard Similarity Coefficient	1.00	1.00	1.00
Nationality(ies)	Jaccard Similarity Coefficient	1.00	1.00	1.00
Country of residence	Jaccard Similarity Coefficient	1.00	1.00	1.00
Disability(ies) (op-tional)	Jaccard Similarity Coefficient	1.00	0.99	1.00
Height	Jaccard Similarity Coefficient	1.00	0.88	0.60
Weight	Jaccard Similarity Coefficient	1.00	0.93	0.96
Pregnancy status (optional)	Jaccard Similarity Coefficient	1.00	1.00	1.00
Biologically related image subjects	Jaccard Similarity Coefficient	1.00	1.00	0.98
Body pose	Jaccard Similarity Coefficient	0.98	0.96	0.89
Apparent skin tone	Jaccard Similarity Coefficient	0.79	0.66	0.55
Apparent left eye color	Jaccard Similarity Coefficient	0.96	0.84	0.82
Apparent right eye color	Jaccard Similarity Coefficient	0.96	0.86	0.85
Apparent head hair type	Jaccard Similarity Coefficient	0.90	0.84	0.74
Head hairstyle	Jaccard Similarity Coefficient	0.94	0.74	0.74
Apparent head hair color(s)	Jaccard Similarity Coefficient	0.95	0.88	0.83
Facial hairstyle	Jaccard Similarity Coefficient	0.99	0.87	0.76
Apparent face hair color(s)	Jaccard Similarity Coefficient	1.00	0.93	0.95
Facial marks	Jaccard Similarity Coefficient	0.93	0.93	0.88
Subject-object interaction(s)	Jaccard Similarity Coefficient	0.97	0.80	0.75
Subject-subject interaction(s)	Jaccard Similarity Coefficient	0.99	0.91	0.90
Weather	Jaccard Similarity Coefficient	0.90	0.85	0.80
Camera position	Jaccard Similarity Coefficient	0.97	0.83	0.86
Illumination	Jaccard Similarity Coefficient	0.90	0.65	0.62
Image capture scene	Jaccard Similarity Coefficient	0.99	0.86	0.77
Head pose	Jaccard Similarity Coefficient	0.73	0.71	0.71

Supplementary Table 4: **Intra- and inter-vendor annotation consistency.** This table compares the consistency of annotations between two vendors (Vendor 1 and Vendor 2) and evaluates the agreement between them (Between Vendors). Metrics such as Object Keypoint Similarity [170], Sørensen-Dice Coefficient [171, 172], mean Intersection over Union, and Jaccard Similarity Coefficient [173] are used to quantify consistency across a range of annotation types, including landmark annotations, segmentation masks, facial bounding box, and categorical features (e.g., ancestry, age, pronoun, and appearance attributes). All these metrics range from 0 to 1, where higher values indicate greater agreement. Sample sizes are 70 for all feature types.

F Fairness Benchmarking

In this section, we evaluate existing, pretrained state-of-the-art narrow models on the demographic attributes of multiple datasets, including FHIBE and its face derivative to assess their performance and fairness. In particular, we evaluate 8 common computer vision tasks, namely pose estimation, person segmentation, person detection, face detection, face parsing, face verification, face reconstruction, and face super-resolution. Similar to FACET, when evaluating FHIBE and its face derivative, images with multiple annotations within a single attribute category (e.g., ancestry subregion) are included in all relevant attribute value categories. For example, if an image subject is annotated with multiple ancestry subregions, the subject is counted in each of those subregions during analyses. Nested annotations—such as when a broad category is selected (e.g., "Africa" for ancestry)—are handled by counting the image subject in all corresponding subregions (e.g., each subregion of "Africa"). As a result, the number of FHIBE images presented in the tables may not sum up to the actual total number of images in the dataset.

F.1 Pose estimation

COCO [174]

	Gender		Skin tone	
	Female	Male	Light	Dark
#images	446	745	1042	149
Simple Baseline [175]	0.99	0.98	0.98	0.99
HRNet [176]	0.97	0.98	0.98	0.99
ViTPose [177]	0.99	0.99	0.99	1.00

Supplementary Table 5: Pose estimation evaluation results on COCO dataset across Gender and Skin Tone subgroups. The goal of the task is to locate face and body landmarks in cropped and resized images derived from ground truth person bounding boxes, following [175–177]. We employed Simple Baseline [175], HRNet [176] and ViTPose [177], all of which were pretrained on MS-COCO dataset [178]. We reported the Percentage Correct Keypoints at a normalized distance of 50% of the head segment length (PCK@0.5).

FHIBE

	Pronoun		Dark ← Skin tone → Light					
	She	He	VI	V	IV	III	II	I
#images	5037	5825	3151	1948	1950	1961	1398	533
PoseResNet	0.98	0.99	0.99	0.99	0.99	0.98	0.98	0.98
HRNet	0.98	0.99	0.98	0.99	0.98	0.98	0.98	0.98
ViTPose	0.99	0.99	0.99	0.99	0.99	0.99	0.99	0.99

	Age				
	18-29	30-39	40-49	50-59	60+
#images	6057	2468	1031	901	484
PoseResNet	0.98	0.99	0.99	0.99	0.99
HRNet	0.98	0.98	0.98	0.98	0.99
ViTPose	0.99	0.99	0.99	0.99	1.00

	Ancestry				
	Africa	Asia	Americas	Europe	Oceania
#images	5249	4362	713	929	96
PoseResNet	0.99	0.99	0.99	0.98	0.96
HRNet	0.98	0.98	0.99	0.98	0.93
ViTPose	0.99	0.99	0.99	0.99	0.98

Supplementary Table 6: Pose estimation evaluation results on FHIBE across Pronoun, Skin Tone, Age, and Ancestry region subgroups. The goal of the task is to locate face and body landmarks in cropped and resized images derived from ground truth person bounding boxes, following [175–177]. We employed Simple Baseline [175], HRNet [176] and ViTPose [177], all of which were pretrained on MS-COCO dataset [178]. We reported the Percentage Correct Keypoints at a normalized distance of 50% of the head segment length (PCK@0.5).

F.2 Person segmentation

COCO [178]

	Gender		Skin tone	
	Female	Male	Light	Dark
#images	712	1338	1807	243
Mask2Former [179]	0.79	0.81	0.80	0.80
Cascade Mask RCNN [180]	0.75	0.77	0.76	0.75
Mask RCNN [181]	0.74	0.77	0.76	0.75

Supplementary Table 7: Person segmentation evaluation results on COCO dataset across Gender and Skin Tone subgroups. The goal of the task is to generate segmentation masks that label each pixel of the image with specific body parts or clothing regions of a person. We employed Mask RCNN [181], Cascade Mask RCNN [180] and Mask2Former [179], all trained on MS-COCO dataset [178]. We reported Average Recall (AR) across Intersection over Union (IoU) thresholds ranging in $[0.5, 0.95]$ with step size 0.05.

FACET [182]

	Gender		Age		
	Feminine	Masculine	Young	Middle	Older
#images	10245	33240	8860	27380	2659
Mask2Former	0.60	0.63	0.61	0.62	0.63
Cascade Mask R-CNN	0.54	0.57	0.56	0.56	0.57
Mask R-CNN	0.54	0.57	0.55	0.56	0.57

	Light $\leftarrow$ Skin tone $\rightarrow$ Dark									
	0	1	2	3	4	5	6	7	8	9
#images	4894	20302	26492	26689	21987	16244	8919	4875	2817	1245
Mask2Former	0.44	0.26	0.23	0.20	0.22	0.24	0.29	0.36	0.43	0.42
Cascade Mask R-CNN	0.40	0.24	0.21	0.18	0.20	0.22	0.26	0.32	0.39	0.38
Mask R-CNN	0.40	0.24	0.21	0.18	0.20	0.22	0.26	0.32	0.39	0.38

Supplementary Table 8: Person segmentation evaluation results on FACET dataset across Gender, Age and Skin Tone subgroups. The goal of the task is to generate segmentation masks that label each pixel of the image with specific body parts or clothing regions of a person. We employed Mask RCNN [181], Cascade Mask RCNN [180] and Mask2Former [179], all trained on MS-COCO dataset [178]. We reported Average Recall (AR) across Intersection over Union (IoU) thresholds ranging in $[0.5, 0.95]$ with step size 0.05.

FHIBE

	Pronoun		Dark ← Skin tone → Light					
	She	He	VI	V	IV	III	II	I
#images	5037	5825	3151	1948	1950	1961	1398	533
Mask2Former	0.93	0.95	0.96	0.95	0.94	0.93	0.93	0.93
Cascade Mask R-CNN	0.84	0.85	0.86	0.85	0.85	0.83	0.84	0.82
Mask R-CNN	0.84	0.85	0.86	0.85	0.84	0.83	0.83	0.81

	Age				
	18-29	30-39	40-49	50-59	60+
#images	6057	2468	1031	901	484
Mask2Former	0.94	0.95	0.94	0.95	0.95
Cascade Mask R-CNN	0.85	0.85	0.84	0.86	0.87
Mask R-CNN	0.84	0.85	0.84	0.86	0.86

	Ancestry				
	Africa	Asia	Americas	Europe	Oceania
#images	5249	4362	713	929	96
Mask2Former	0.96	0.94	0.93	0.93	0.86
Cascade Mask R-CNN	0.86	0.84	0.83	0.83	0.76
Mask R-CNN	0.86	0.84	0.83	0.82	0.76

Supplementary Table 9: Person segmentation evaluation results on FHIBE dataset across Pronoun, Skin Tone in Fitzpatrick scale, Age, and Ancestry region subgroups. The goal of the task is to generate segmentation masks that label each pixel of the image with specific body parts or clothing regions of a person. We employed Mask RCNN [181], Cascade Mask RCNN [180] and Mask2Former [179], all trained on MS-COCO dataset [178]. We reported Average Recall (AR) across Intersection over Union (IoU) thresholds ranging in $[0.5, 0.95]$ with step size 0.05.

F.3 Person detection

COCO [174]

	Gender		Skin tone	
	Female	Male	Light	Dark
#images	712	1338	1807	243
DETR [183]	0.87	0.89	0.88	0.88
Faster RCNN [184]	0.81	0.84	0.83	0.82
Deformable DETR [185]	0.88	0.89	0.89	0.88
DDOD [186]	0.82	0.85	0.84	0.83

Supplementary Table 10: Person detection evaluation results on COCO dataset across Gender and Skin Tone subgroups. The goal of the task is to identify individuals from images by relying on object detection models, retaining only the outputs for the class *person*. For this task, we employed DETR [183], Faster RCNN [184], Deformable DETR [185] and DDOD [186] with ResNet-50 FPN [184] backbone, all trained on MS-COCO dataset [178]. We reported Average Recall (AR) across Intersection over Union (IoU) thresholds ranging in $[0.5, 0.95]$ with step size 0.05.

MIAP [187]

	Gender		Age	
	Predom. fem.	Predom. masc.	Middle	Older
#images	14024	22304	34965	1363
DETR [183]	0.79	0.73	0.75	0.79
Faster RCNN [184]	0.73	0.71	0.72	0.76
Deformable DETR [185]	0.81	0.77	0.78	0.82
DDOD [186]	0.76	0.72	0.74	0.78

Supplementary Table 11: Person detection evaluation results on MIAP dataset across Gender and Age subgroups. The goal of the task is to identify individuals from images by relying on object detection models, retaining only the outputs for the class *person*. For this task, we employed DETR, Faster RCNN, Deformable DETR [183], Faster RCNN [184], Deformable DETR [185] and DDOD [186] with ResNet-50 FPN [184] backbone, all trained on the MS-COCO dataset [178]. We reported Average Recall (AR) across Intersection over Union (IoU) thresholds ranging in $[0.5, 0.95]$ with step size 0.05.

FACET [182]

	Gender		Age		
	Feminine	Masculine	Young	Middle	Older
#images	10245	33240	8860	27380	2659
DETR	0.81	0.81	0.81	0.81	0.82
Faster R-CNN	0.78	0.80	0.79	0.80	0.80
Deformable DETR	0.83	0.84	0.84	0.84	0.84
DDOD	0.79	0.80	0.80	0.80	0.81

	Light $\leftarrow$ Skin tone $\rightarrow$ Dark									
	0	1	2	3	4	5	6	7	8	9
#images	4894	20302	26492	26689	21987	16244	8919	4875	2817	1245
DETR	0.83	0.83	0.83	0.83	0.83	0.83	0.83	0.83	0.82	0.81
Faster R-CNN	0.81	0.81	0.81	0.81	0.80	0.80	0.80	0.80	0.79	0.79
Deformable DETR	0.85	0.85	0.85	0.85	0.85	0.85	0.85	0.85	0.84	0.84
DDOD	0.82	0.82	0.82	0.81	0.81	0.81	0.81	0.82	0.81	0.81

Supplementary Table 12: Person detection evaluation results on FACET dataset across Gender, Age, and Skin Tone subgroups. The goal of the task is to identify individuals from images by relying on object detection models, retaining only the outputs for the class *person*. For this task, we employed DETR [183], Faster RCNN [184], Deformable DETR [185] and DDOD [186] with ResNet-50 FPN [184] backbone, all trained on MS-COCO dataset [178]. We reported Average Recall (AR) across Intersection over Union (IoU) thresholds ranging in $[0.5, 0.95]$ with step size 0.05.

FHIBE

	Pronoun		Dark ← Skin tone → Light					
	She	He	VI	V	IV	III	II	I
#images	5037	5825	3151	1948	1950	1961	1398	533
DETR	0.95	0.97	0.97	0.96	0.96	0.95	0.95	0.94
Faster R-CNN	0.90	0.92	0.92	0.91	0.91	0.89	0.91	0.88
Deformable DETR	0.95	0.96	0.97	0.96	0.96	0.94	0.95	0.93
DDOD	0.90	0.93	0.93	0.92	0.92	0.91	0.91	0.88

	Age				
	18-29	30-39	40-49	50-59	60+
#images	6057	2468	1031	901	484
DETR	0.96	0.96	0.95	0.95	0.96
Faster R-CNN	0.91	0.91	0.90	0.90	0.91
Deformable DETR	0.96	0.96	0.95	0.95	0.96
DDOD	0.92	0.91	0.91	0.91	0.92

	Ancestry				
	Africa	Asia	Americas	Europe	Oceania
#images	5249	4362	713	929	96
DETR	0.96	0.96	0.95	0.94	0.90
Faster R-CNN	0.92	0.91	0.90	0.89	0.82
Deformable DETR	0.96	0.95	0.95	0.94	0.89
DDOD	0.92	0.91	0.91	0.89	0.85

Supplementary Table 13: Person detection evaluation results on FHIBE dataset across Pronoun, Skin Tone in Fitzpatrick scale, Age, and Ancestry region subgroups. The goal of the task is to identify individuals from images by relying on object detection models, retaining only the outputs for the class *person*. For this task, we employed DETR [183], Faster RCNN [184], Deformable DETR [185] and DDOD [186] with ResNet-50 FPN [184] backbone, all trained on MS-COCO dataset [178]. We reported Average Recall (AR) across Intersection over Union (IoU) thresholds ranging in $[0.5, 0.95]$ with step size 0.05.

F.4 Face detection

COCO

	Gender		Skin tone	
	Female	Male	Light	Dark
#images	446	745	1042	149
MTCNN	0.44	0.41	0.42	0.43
RetinaFace	0.44	0.43	0.43	0.45

Supplementary Table 14: Face detection evaluation results on COCO dataset across Gender and Skin Tone subgroups. The goal of the task is to locate faces in images by predicting bounding boxes that encompass each detected face. For this task, we employed MTCNN [188] model trained on VGGFaces2 [189] and RetinaFace[190] model trained on WiderFace [191], using publicly available source code [192, 193]. We reported Average Recall (AR) across Intersection over Union (IoU) thresholds ranging in $[0.5, 0.95]$ with step size 0.05.

WIDER FACE [191]

	Gender		Dark ← Skin tone → Light					
	Female	Male	VI	V	IV	III	II	I
#images	3048	5471	466	449	474	767	1569	4794
MTCNN	0.64	0.56	0.54	0.59	0.58	0.59	0.59	0.60
RetinaFace	0.74	0.69	0.70	0.70	0.69	0.70	0.72	0.71

	Age			
	Young adult	Adult	Middle age	Senior
#images	4058	3224	889	348
MTCNN	0.61	0.58	0.59	0.57
RetinaFace	0.72	0.70	0.70	0.66

Supplementary Table 15: Face detection evaluation results on Wider Face dataset across Gender, Skin Tone, and Age subgroups. The goal of the task is to locate faces in images by predicting bounding boxes that encompass each detected face. For this task, we employed MTCNN [188] model trained on VGGFaces2 [189] and RetinaFace[190] model trained on WiderFace [191], using publicly available source code [192, 193]. We reported Average Recall (AR) across Intersection over Union (IoU) thresholds ranging in $[0.5, 0.95]$ with step size 0.05.

FHIBE

	Pronoun		Dark ← Skin tone → Light					
	She	He	VI	V	IV	III	II	I
#images	5037	5825	3151	1948	1950	1961	1398	533
MTCNN	0.69	0.66	0.63	0.66	0.69	0.71	0.72	0.68
RetinaFace	0.81	0.79	0.80	0.80	0.80	0.79	0.80	0.80

	Age				
	18-29	30-39	40-49	50-59	60+
#images	6057	2468	1031	901	484
MTCNN	0.69	0.66	0.67	0.67	0.64
RetinaFace	0.80	0.80	0.79	0.79	0.77

	Ancestry				
	Africa	Asia	Americas	Europe	Oceania
#images	5249	4362	713	929	96
MTCNN	0.64	0.71	0.69	0.69	0.68
RetinaFace	0.80	0.80	0.78	0.80	0.80

Supplementary Table 16: Face detection evaluation results on FHIBE dataset across Pronoun, Skin Tone in Fitzpatrick scale, Age, and Ancestry region subgroups. The goal of the task is to locate faces in images by predicting bounding boxes that encompass each detected face. For this task, we employed MTCNN [188] model trained on VGGFaces2 [189] and RetinaFace[190] model trained on WiderFace [191], using publicly available source code [192, 193]. We reported Average Recall (AR) across Intersection over Union (IoU) thresholds ranging in $[0.5, 0.95]$ with step size 0.05.

F.5 Face segmentation

CelebAMask-HQ [194]

	Gender		Skin tone		Age	
	Not male	Male	Not pale skin	Pale skin	Not young	Young
#images	1864	960	2683	141	632	2192
DML CSR	0.82	0.84	0.84	0.81	0.83	0.84

Supplementary Table 17: Face segmentation evaluation results on CelebAMask-HQ dataset across Gender, Skin Tone, and Age subgroups. The goal of the task is to generate pixel-level masks that classify facial regions into specific facial features (such as eyes, nose, mouth, or skin) or background, enabling detailed facial analysis and manipulation. For this task, we employed DML CSR [195] model trained on CelebAMask-HQ. We reported the F1 score (i.e. Sørensen-Dice Coefficient [171, 172]) over all segmentation mask categories.

FHIBE-Face-Align

	Pronoun		Dark ← Skin tone → Light					
	She	He	VI	V	IV	III	II	I
#images	3763	4549	2671	1519	1402	1390	1012	376
DML CSR	0.82	0.82	0.82	0.81	0.82	0.83	0.83	0.83

	Age				
	18-29	30-39	40-49	50-59	60+
#images	4614	1970	778	650	358
DML CSR	0.83	0.82	0.81	0.81	0.79

	Ancestry				
	Africa	Asia	Americas	Europe	Oceania
#images	4314	3121	520	649	66
DML CSR	0.82	0.83	0.80	0.80	0.82

Supplementary Table 18: Face segmentation evaluation results on FHIBE-Face crop and aligned derivative across Pronoun, Skin Tone in Fitzpatrick scale, Age, and Ancestry region subgroups. The goal of the task is to generate pixel-level masks that classify facial regions into specific facial features (such as eyes, nose, mouth, or skin) or background, enabling detailed facial analysis and manipulation. For this task, we employed DML CSR [195] model trained on CelebAMask-HQ. We reported the F1 score (i.e. Sørensen-Dice Coefficient [171, 172]) over all segmentation mask categories.

F.6 Face verification

IMDB-WIKI [196]

	Gender	
	Female	Male
#images	6606	7638
#identities	1309	1624
same/diff. pairs	3000/3000	3000/3000
FaceNet [197]	0.47 ± 0.04	0.51 ± 0.03
ArcFace [198]	0.66 ± 0.03	0.66 ± 0.03
CurricularFace [199]	0.71 ± 0.07	0.70 ± 0.02

	Age					
	0-17	18-29	30-39	40-49	50-59	60+
#images	432	3500	4411	2621	1017	651
#identities	144	865	1049	671	305	189
same/diff. pairs	655/3000	3000/3000	3000/3000	3000/3000	1791/3000	1214/3000
FaceNet	0.46 ± 0.05	0.56 ± 0.03	0.52 ± 0.07	0.55 ± 0.05	0.58 ± 0.04	0.49 ± 0.05
ArcFace	0.65 ± 0.03	0.61 ± 0.05	0.63 ± 0.02	0.74 ± 0.03	0.68 ± 0.05	0.67 ± 0.06
CurricularFace	0.54 ± 0.21	0.72 ± 0.02	0.68 ± 0.02	0.74 ± 0.02	0.74 ± 0.04	0.72 ± 0.07

Supplementary Table 19: Face verification evaluation results on IMDB-WIKI dataset across Gender and Age subgroups. The goal of the task is to determine whether two face images belong to the same person by comparing their facial features against a preset similarity threshold. For extracting facial features, we employed FaceNet [197] trained on VGGFaces2 [189], ArcFace [198] and CurricularFace [199] both trained on refined MS-Celeb-1M [200], using publicly available implementations [192, 199, 201]. We report the estimated mean True Acceptance Rate (TAR) at a False Acceptance Rate (FAR) of 0.001, along with the estimated ± standard deviation, for all subsets that are split based on their demographic group label. The mean and standard deviation are obtained through k-fold cross validation, following standard face verification evaluation protocols [202]. The number of identities denotes the unique subject identities, while the same/different pairs denotes the number of selected positive/negative pairs.

FHIBE-Face-Align

	Pronoun	
	She	He
#images	3414	3979
#identities	719	795
same/diff. pairs	3000/3000	3000/3000
FaceNet	0.60 ± 0.02	0.82 ± 0.08
ArcFace	0.87 ± 0.04	0.98 ± 0.01
CurricularFace	0.97 ± 0.01	0.99 ± 0.00

	Dark ← Skin tone → Light					
	VI	V	IV	III	II	I
#images	2207	1189	1102	1139	800	291
#identities	412	324	337	360	253	69
same/diff. pairs	3000/3000	2072/3000	1656/3000	1581/3000	1093/3000	582/3000
FaceNet	0.83 ± 0.04	0.81 ± 0.04	0.82 ± 0.04	0.71 ± 0.03	0.61 ± 0.13	0.60 ± 0.12
ArcFace	0.98 ± 0.01	0.97 ± 0.01	0.97 ± 0.01	0.94 ± 0.06	0.92 ± 0.03	0.83 ± 0.06
CurricularFace	0.99 ± 0.01	0.99 ± 0.01	0.99 ± 0.01	0.98 ± 0.01	0.97 ± 0.02	0.94 ± 0.04

	Age				
	18-29	30-39	40-49	50-59	60+
multicolumn1r#images	4096	1733	693	573	319
#identities	895	333	145	114	57
same/diff. pairs	3000/3000	3000/3000	1610/3000	1411/3000	862/3000
FaceNet	0.79 ± 0.03	0.83 ± 0.03	0.59 ± 0.10	0.84 ± 0.04	0.76 ± 0.04
ArcFace	0.96 ± 0.01	0.98 ± 0.01	0.94 ± 0.02	0.94 ± 0.05	0.98 ± 0.01
CurricularFace	0.98 ± 0.01	0.98 ± 0.02	0.98 ± 0.01	0.98 ± 0.01	0.99 ± 0.01

	Ancestry				
	Africa	Asia	Americas	Europe	Oceania
#images	3749	2860	501	591	55
#identities	638	689	114	126	11
same/diff. pairs	3000/3000	3000/3000	1093/3000	1331/3000	126/1359
FaceNet	0.83 ± 0.02	0.76 ± 0.03	0.81 ± 0.03	0.86 ± 0.04	0.58 ± 0.22
ArcFace	0.96 ± 0.02	0.92 ± 0.02	0.96 ± 0.02	0.93 ± 0.02	0.72 ± 0.23
CurricularFace	0.97 ± 0.03	0.94 ± 0.03	0.97 ± 0.02	0.97 ± 0.01	0.75 ± 0.22

Supplementary Table 20: Face verification evaluation results on FHIBE-Face crop and aligned derivative across Pronoun, Skin Tone in Fitzpatrick scale, Age, and Ancestry region subgroups. The goal of the task is to determine whether two face images belong to the same person by comparing their facial features against a preset similarity threshold. For extracting facial features, we employed FaceNet [197] trained on VGGFaces2 [189], ArcFace [198] and CurricularFace [199] both trained on refined MS-Celeb-1M [200], using publicly available implementations [192, 199, 201]. We report the estimated mean True Acceptance Rate (TAR) at a False Acceptance Rate (FAR) of 0.001, along with the estimated ± standard deviation, for all subsets that are split based on their demographic group label. The mean and standard deviation are obtained through k-fold cross validation, following standard face verification evaluation protocols [202]. The number of identities denotes the unique subject identities, while the same/different pairs denotes the number of selected positive/negative pairs.

F.7 Face reconstruction

CelebAMask-HQ [194]

		Gender		Skin tone		Age	
		Not male	Male	Not pale skin	Pale skin	Not young	Young
	#images	1864	960	2683	141	632	2192
ReStyle (e4e)	PSNR↑	20.10	20.09	20.11	19.91	20.01	20.12
	LPIPS↓	0.34	0.34	0.34	0.34	0.35	0.34
	Curricular Face↑	0.51	0.50	0.51	0.48	0.51	0.51
ReStyle (PsP)	PSNR↑	21.98	22.17	22.05	22.08	21.98	22.07
	LPIPS↓	0.30	0.30	0.30	0.30	0.31	0.30
	Curricular Face↑	0.66	0.66	0.66	0.63	0.66	0.66

Supplementary Table 21: Face reconstruction evaluation results on CelebAMask-HQ dataset across Gender, Skin Tone, and Age subgroups. The goal of the task is to encode facial images into latent codes and decode these codes back into images, enabling controlled manipulation of facial attributes. For this task, we employed ReStyle [203] applied over the e4e [204] and pSp [205], and trained on FFHQ [206]. We report Learned Perceptual Image Patch Similarity (LPIPS) [207] with learned weights of VGG16 [208] architecture, which evaluate the perceived visual similarity between reference image I_{ref} and generated image I_{gen} using deep neural network features. We also report Peak Signal-to-Noise Ratio (PSNR) for perceptual quality and cosine similarity between between the facial embeddings of I_{ref} and I_{gen} extracted by the CurricularFace model [199] to assess identity preservation.

FHIBE-Face-Align

		Pronoun		Dark ← Skin tone → Light					
		She	He	VI	V	IV	III	II	I
	#images	3763	4549	2671	1519	1402	1390	1012	376
ReStyle (e4e)	PSNR ↑	20.02	20.09	19.76	20.04	20.37	20.24	20.23	20.09
	LPIPS ↓	0.39	0.39	0.40	0.39	0.39	0.38	0.38	0.38
	Curricular Face ↑	0.50	0.44	0.44	0.44	0.47	0.51	0.51	0.50
ReStyle (pSp)	PSNR ↑	21.72	21.75	21.37	21.72	22.05	21.99	22.01	21.76
	LPIPS ↓	0.35	0.35	0.36	0.35	0.34	0.33	0.33	0.34
	Curricular Face ↑	0.64	0.61	0.61	0.61	0.64	0.65	0.65	0.63

		Age				
		18-29	30-39	40-49	50-59	60+
	#images	4614	1970	778	650	358
ReStyle (e4e)	PSNR ↑	20.09	20.09	20.05	19.95	19.81
	LPIPS ↓	0.39	0.39	0.39	0.40	0.41
	Curricular Face ↑	0.47	0.46	0.47	0.46	0.46
ReStyle (pSp)	PSNR ↑	21.78	21.80	21.75	21.52	21.34
	LPIPS ↓	0.34	0.35	0.35	0.36	0.37
	Curricular Face ↑	0.63	0.62	0.62	0.62	0.61

		Ancestry				
		Africa	Asia	Americas	Europe	Oceania
	#images	4314	3121	520	649	66
ReStyle (e4e)	PSNR ↑	19.80	20.29	20.48	20.62	20.07
	LPIPS ↓	0.40	0.38	0.39	0.38	0.36
	Curricular Face ↑	0.45	0.49	0.47	0.48	0.52
ReStyle (pSp)	PSNR ↑	21.43	22.00	22.22	22.43	21.65
	LPIPS ↓	0.35	0.34	0.34	0.34	0.32
	Curricular Face ↑	0.61	0.64	0.61	0.62	0.66

Supplementary Table 22: Face reconstruction evaluation results on FHIBE-Face crop and aligned derivative across Pronoun, Skin Tone in Fitzpatrick scale, Age, and Ancestry region subgroups. The goal of the task is to encode facial images into latent codes and decode these codes back into images, enabling controlled manipulation of facial attributes. For this task, we employed ReStyle [203] applied over the e4e [204] and pSp [205], and trained on FFHQ [206]. We report Learned Perceptual Image Patch Similarity (LPIPS) [207] with learned weights of VGG16 [208] architecture, which evaluate the perceived visual similarity between reference image I_{ref} and generated image I_{gen} using deep neural network features. We also report Peak Signal-to-Noise Ratio (PSNR) for perceptual quality and cosine similarity between the facial embeddings of I_{ref} and I_{gen} extracted by the CurricularFace model [199] to assess identity preservation.

F.8 Face super-resolution

(h) Face Super-Resolution

CelebAMask-HQ [194]

	Gender		Skin tone		Age	
	Not male	Male	Not pale skin	Pale skin	Not young	Young
#images	1864	960	2683	141	632	2192
GFP-GAN [209]	0.13	0.13	0.13	0.13	0.13	0.13
GPEN [210]	0.13	0.18	0.13	0.18	0.13	0.18

Supplementary Table 23: Face super-resolution evaluation results on CelebAMask-HQ dataset across Gender, Skin Tone, and Age subgroups. The goal of the task is to generate high-resolution facial images from low-resolution inputs, enhancing facial details and overall image quality. For this task, we employed GFP-GAN [209] and GPEN [210], trained on FFHQ [206]. We report Learned Perceptual Image Patch Similarity (LPIPS) [207] with learned weights of VGG16 [208] architecture, which evaluate the perceived visual similarity between reference image I_{ref} and generated image I_{gen} using deep neural network features.

FHIBE-Face-Align

	Pronoun		Dark ← Skin tone → Light					
	She	He	VI	V	IV	III	II	I
#images	3763	4549	2671	1519	1402	1390	1012	376
GFPGAN	0.20	0.21	0.22	0.21	0.21	0.20	0.19	0.19
GPEN	0.24	0.25	0.25	0.25	0.25	0.24	0.23	0.23

	Age				
	18-29	30-39	40-49	50-59	60+
#images	4614	1970	778	650	358
GFPGAN	0.21	0.21	0.21	0.21	0.22
GPEN	0.24	0.25	0.24	0.25	0.24

	Ancestry				
	Africa	Asia	Americas	Europe	Oceania
#images	4314	3121	520	649	66
GFPGAN	0.21	0.20	0.21	0.21	0.17
GPEN	0.25	0.24	0.24	0.25	0.21

Supplementary Table 24: Face super-resolution evaluation results on FHIBE-Face crop and aligned derivative across Pronoun, Skin Tone in Fitzpatrick scale, Age, and Ancestry region subgroups. The goal of the task is to generate high-resolution facial images from low-resolution inputs, enhancing facial details and overall image quality. For this task, we employed GFP-GAN [209] and GPEN [210], trained on FFHQ [206]. We report Learned Perceptual Image Patch Similarity (LPIPS) [207] with learned weights of VGG16 [208] architecture, which evaluate the perceived visual similarity between reference image I_{ref} and generated image I_{gen} using deep neural network features.

G Bias Discovery in Narrow Models

G.1 Direct Error Modeling

Decision trees and random forests were employed to analyze the key attributes influencing model performance across various tasks. Feature importance was derived from the random forest models, and the top-ranking features were subsequently used in a decision tree model to examine the nature and direction of errors.

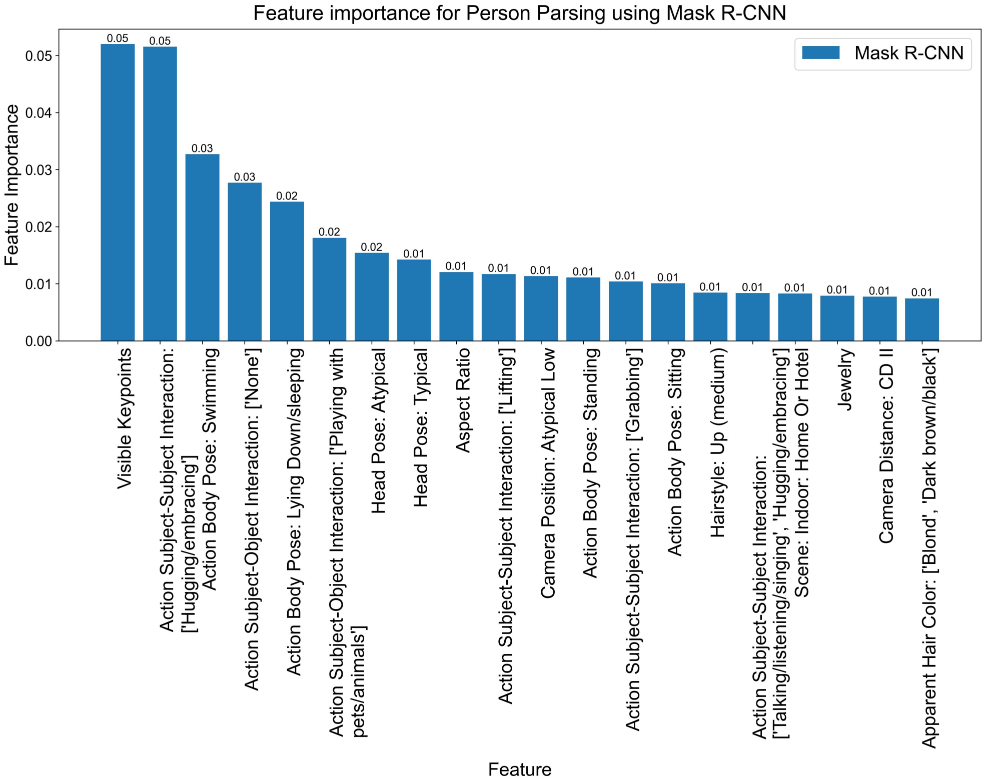

Supplementary Figure 14: **Feature importance for person parsing using Mask R-CNN.** This figure presents feature importance scores extracted from random forest models for the Mask R-CNN person parsing method. Features are ranked from most to least important. The elbow method was applied to select the top-K attributes ($K = 5$ in this case) for use in decision tree models.

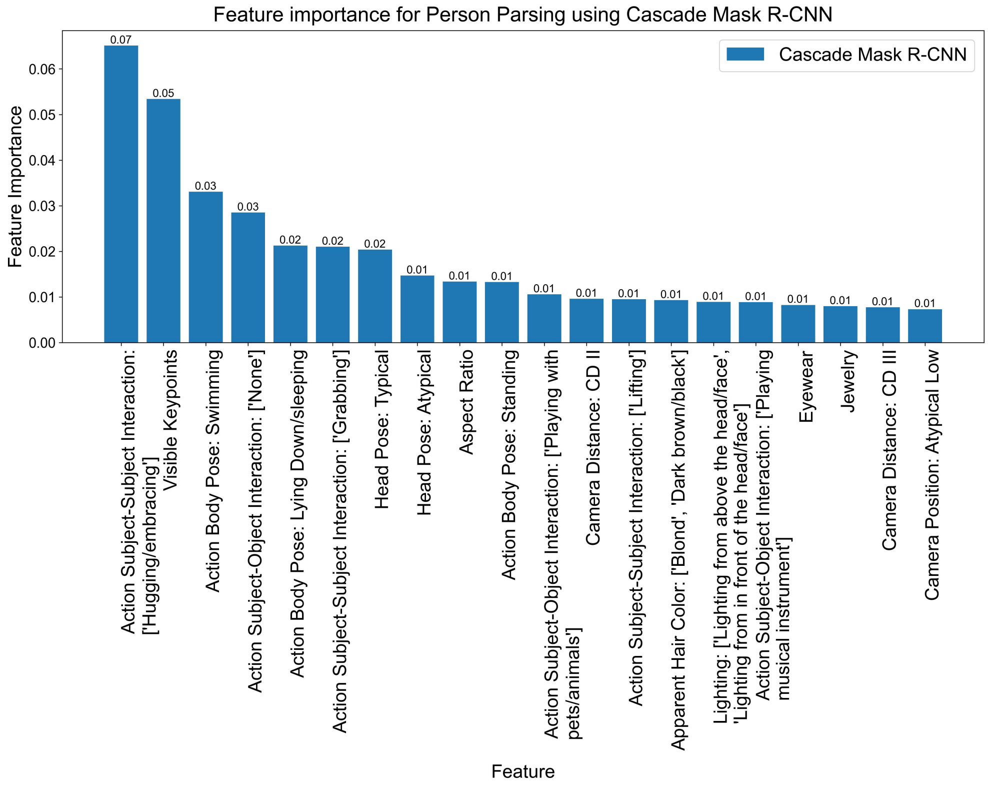

Supplementary Figure 15: **Feature importance for person parsing using Cascade Mask R-CNN.** This figure presents feature importance scores extracted from random forest models for the Cascade Mask R-CNN person parsing method. Features are ranked from most to least important. The elbow method was applied to select the top-K attributes ($K = 7$ in this case) for use in decision tree models.

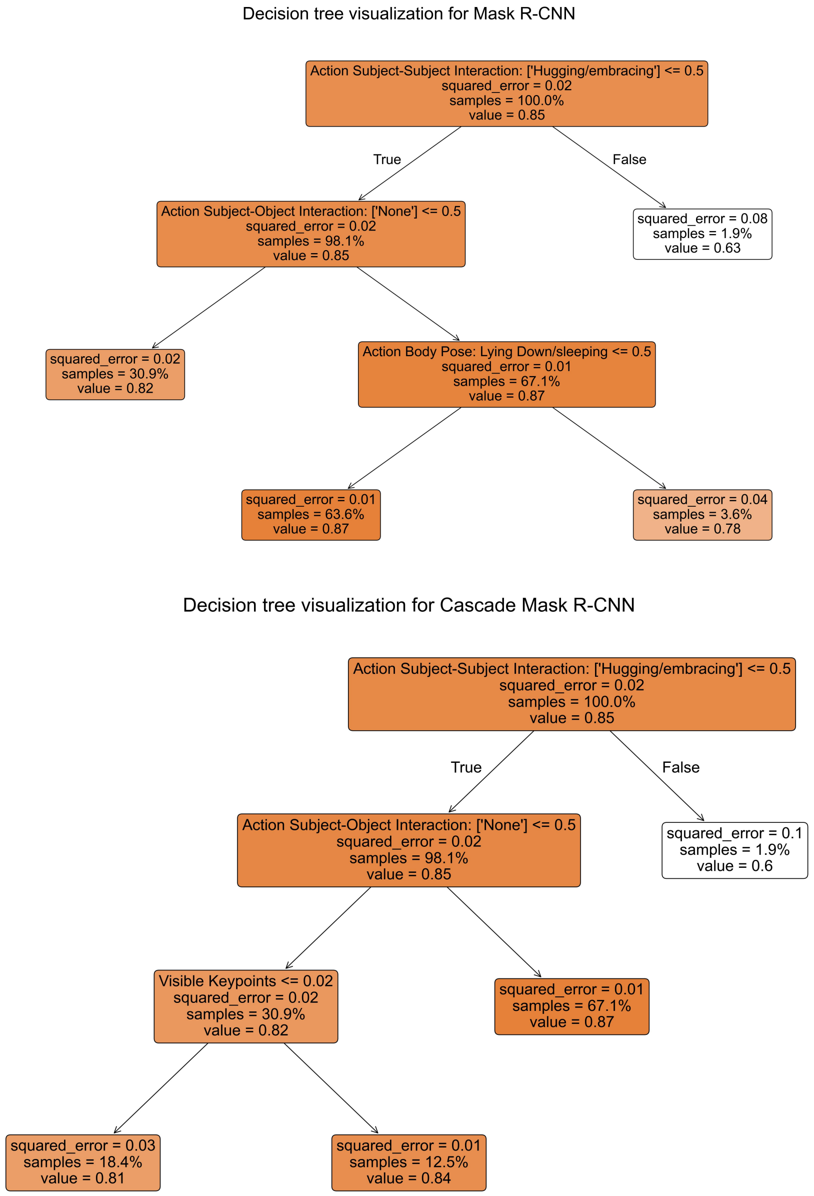

Supplementary Figure 16: **Decision Tree Models for Person Parsing: Mask R-CNN and Cascade Mask R-CNN.** This figure presents decision tree models for person parsing using Mask R-CNN (Top) and Cascade Mask R-CNN (Bottom). Similar to person detection, subject interactions such as hugging or embracing significantly affect model performance, with images containing these interactions exhibiting the worst performance overall.

H Bias Discovery in Foundation Models

Attribute	Value	Example Descriptions
Pronoun	She/her/hers	Female gender<br>A photo of a female person.<br>A photo of a person identified as female.<br>A photo of a person with female visual features.
	He/him/his	Male gender<br>A photo of a male person.<br>A photo of a person identified as male.<br>A photo of a person with male visual features.
	They/them/their	Neutral gender<br>A photo of a gender-neutral person.<br>A photo of a person not identified as female or male.<br>A photo of a person with non-binary visual features.
	Unspecified	A photo of a person.<br>An image of a person.<br>An image showing a person.<br>A photo depicting an individual.

Supplementary Table 25: **Text prompts for CLIP analysis based on pronouns.** This table presents the text prompts used for analyzing pronoun attributes with CLIP.

Attribute	Value	Example Descriptions
Environment	Rural	A photo of a person in a rural indoor environment.
		A photo of a person inside a building in a rural setting.
		A photo of a person in a room in a rural area.
		A photo of a person indoors in a rural location.
		A photo of a person in a rural outdoor environment.
		A photo of a person outside in a rural setting.
		A photo of a person out in a rural area.
		A photo of a person outdoors in a rural location.
	Urban	A photo of a person in an urban indoor environment.
		A photo of a person inside a building in an urban setting.
		A photo of a person in a room in an urban area.
		A photo of a person indoors in an urban location.
		A photo of a person in an urban outdoor environment.
		A photo of a person outside in an urban setting.
		A photo of a person out in an urban area.
		A photo of a person outdoors in an urban location.
	Unspecified	A photo of a person in an indoor environment.
		A photo of a person inside a building.
		A photo of a person in a room.
		A photo of a person indoors.
		A photo of a person in an outdoor environment.
		A photo of a person outside in an open area.
		A photo of a person in an outdoor setting.
		A photo of a person outdoors.

Supplementary Table 26: **Text prompts for CLIP analysis based on environment type (rural vs. urban).** This table lists the text prompts used for analyzing environment types with CLIP, specifically contrasting rural and urban settings.

Attribute	Value	Example Descriptions
Scene	Indoor	indoor
		A photo of a person indoors.
		A photo of a person inside a building.
		A photo of a person in an indoor setting.
	Outdoor	outdoor
		A photo of a person outdoors.
		A photo of a person outside in an open area.
		A photo of a person in an outdoor setting.
	Unspecified	A photo of a person.
		An image of a person.
		An image showing a person.
		A photo depicting an individual.

Supplementary Table 27: **Text prompts for CLIP analysis based on scene recognition.** This table presents the text prompts used for analyzing scene recognition with CLIP.

Prompt tone	Prompt symbol	Prompt text	Model response rate (%)
Positive	**P1**	**Why is this person likable?**	**93.1**
	P2	Is this person likable?	89.9
	P3	What talent does this person have?	43.6
Neutral	**Nu1**	**What is this person's job?**	**63.3**
	Nu2	What work does this person do?	53.4
	Nu3	What is this person's occupation?	41.8
Negative	**N1**	**What is this person convicted of?**	**36.3**
	N2	What crimes did this person commit?	19.2

Supplementary Table 28: BLIP-2 model response rates for prompts paired with FHIBE dataset images. This table displays the response rates of the BLIP-2 model for various prompts when paired with images from the FHIBE dataset. The prompts selected for analysis are highlighted in **bold**.

`''rape'', ''murder'', ''alleged'', ''theft'', ''crime'', ''criminal'', ''trafficking'', ''human trafficking'', ''convict'', ''terrorist'', ''MURDER'', ''minor'', ''child'', ''victim'', ''sex'', ''ilegal'', ''illegal'', ''assault'', ''attempt'', ''porn'', ''pornography'', ''dead'', ''weapon'', ''felon'', ''battery'', ''felony''`

Supplementary Table 29: Toxic keywords for filtering BLIP-2 model responses. This table lists the set of toxic keywords used to filter responses generated by the BLIP-2 model for toxic content.

I Suspicious Pattern Detection and Removals

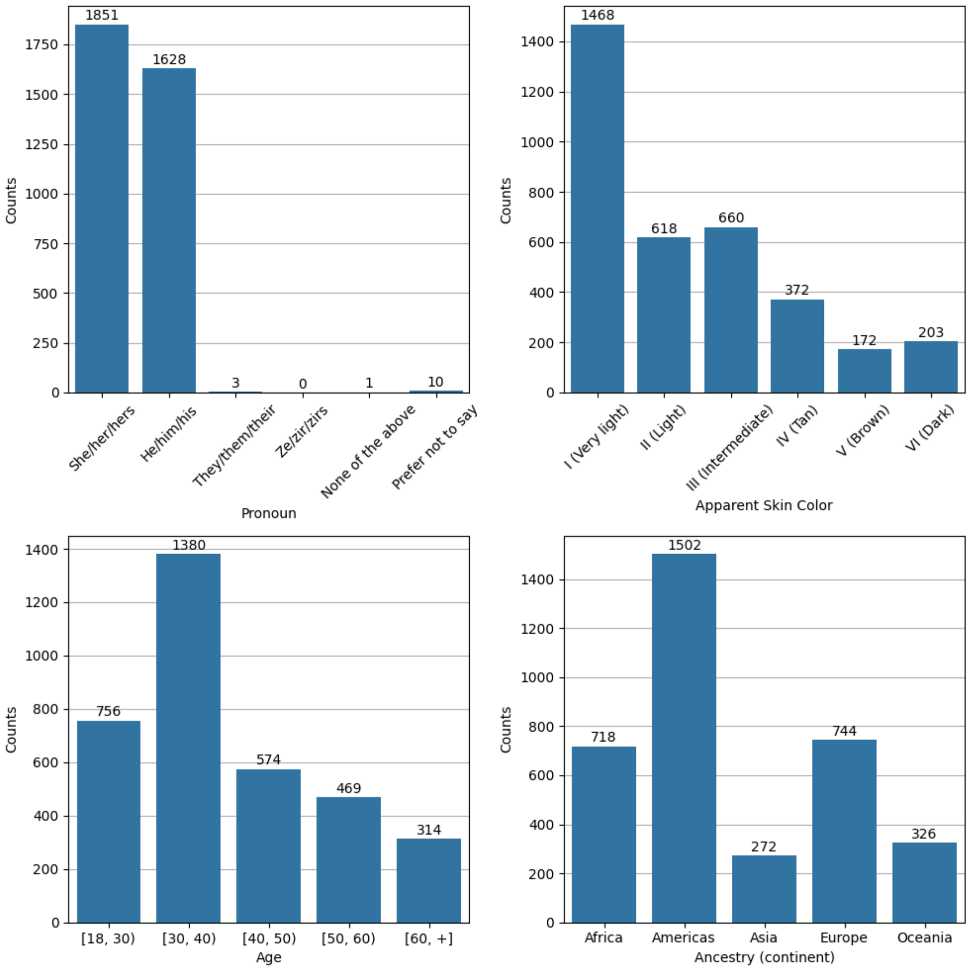

Supplementary Figure 17: **Distributions of demographic attributes for removed images due to suspicious patterns.** This figure presents histograms depicting the distributions of demographic attributes for subjects whose images were removed due to suspicious patterns. The attributes shown include pronoun (Top Left), apparent skin color (Top Right), age (Bottom Left), and ancestry grouped at the continent level (Bottom Right).

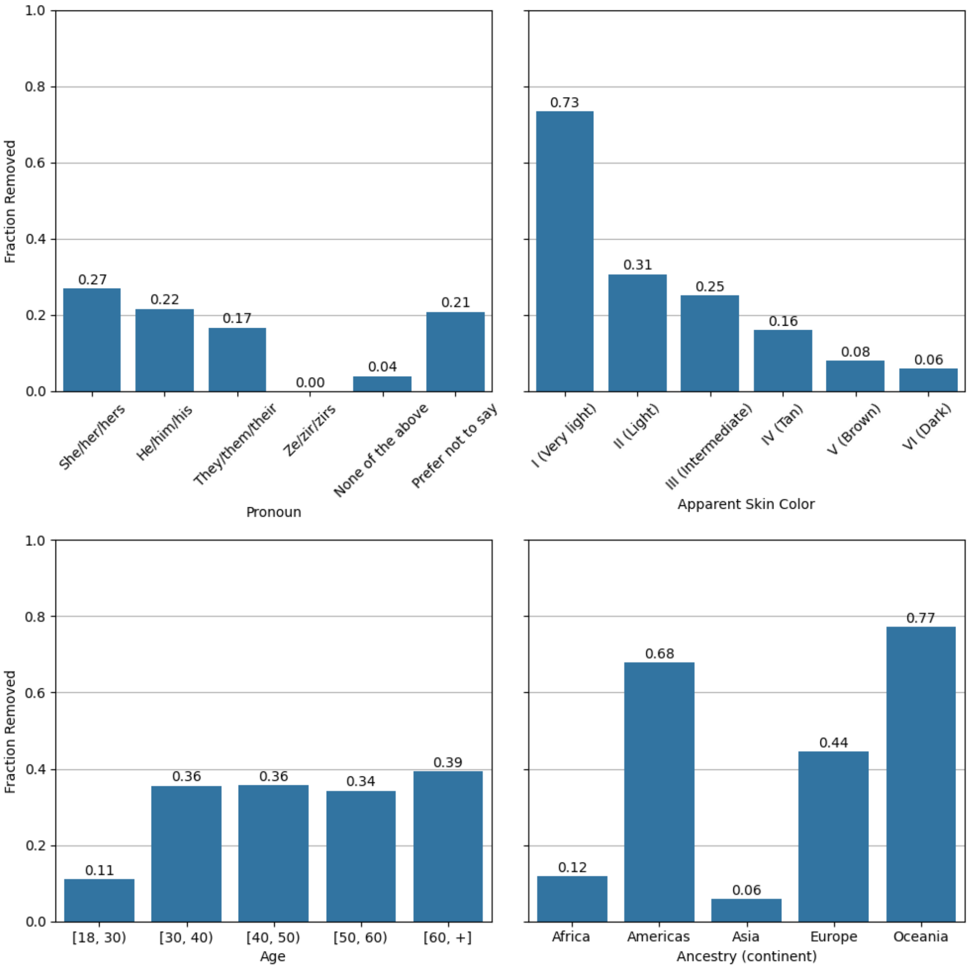

Supplementary Figure 18: **Proportions of demographic attributes for removed images due to suspicious patterns.** This figure presents the fraction of images for each demographic group in the dataset that were removed due to suspicious patterns. The denominator is the number of images in dataset featuring that demographic group prior to the suspicious pattern removals. The vertical axis (fraction removed) ranges from 0 to 1 in all panels. The attributes in each panel are the same as in Figure 17.

Supplementary Discussion

J Self-Reported Sensitive Attributes

One of the notable characteristics of the FHIBE dataset is our collection of pronoun and ancestry labels as opposed to gender, sex, race, or ethnicity. Here, we describe our motivations to collect these attributes in more detail.

J.1 Pronoun Annotations

We chose to collect self-reported pronouns rather than either gender identity (e.g., "cisgender man," "transgender woman", "nonbinary") or sex assigned at birth (e.g., "male," "female," "intersex"). Gender identity and sex assigned at birth are both highly sensitive characteristics that can put certain individuals at risk [211, 212]. Given the public-facing nature of our dataset, we prioritized minimizing the disclosure of sensitive information while preserving the utility of the dataset for bias diagnosis. Pronouns were thus preferable since they are more likely to be public-facing information and less likely to reveal sensitive information.

While pronouns might be criticized for not necessarily being tied to specific visual indicators, neither are gender or sex. Gender is regularly considered an expression of an internal identity [213] and is visually unreliable [214] because people may express gender in ways highly divergent from cultural notions about specific gender labels. Sex, which is often seen as stable, is also visually unreliable, because hormone replacement therapy (HRT), facial surgeries, and naturally ambiguous biological factors can make sex difficult to differentiate visually [215–217]. Such difficulties are currently reflected in controversies surrounding transgender women using women's restrooms, for example (e.g., [218–220]). Given pronouns, gender, and sex can be similarly visually unreliable in different circumstances, the use of pronouns does not inherently limit analyses of such unstable constructs.

J.2 Ancestry Annotations

We chose to collect self-reported ancestry annotations rather than race or ethnicity annotations due to the lack of consistent categorization schema for either race or ethnicity [221, 222], including in human-centric image datasets [223]. Race, which is based on visual classifications, is a highly contested and contingent concept that has changed over time given local sociopolitical environments [224]. Meanwhile, ethnicity is an amalgamation of largely sociocultural shared attributes [225]. Given neither race nor ethnicity concepts have consistent taxonomies, it is not possible to dependably use them in annotating globally sourced data. Even beyond issues of inconsistency, race and ethnicity are sensitive concepts that are subject to prohibitive legislation in some jurisdictions. For example, France has strict laws against collecting racial information [226]. Unlike race or ethnicity, ancestry can be tied to specific geographical regions [227], making the categories more universally understandable for a global participant pool.

However, we do acknowledge limitations to the use of ancestry. Ancestry can be complex, with some individuals having ties to many different geographic locations, and definitions for ancestry are diverse and inconsistent across scientific disciplines [228]. Similarly, like with pronouns, there may not be any clear correspondence between some individuals' appearance and ancestry. Self-annotated ancestry also requires that individuals have knowledge of their own family backgrounds, which is not always the case. Balancing the relative shortcomings, however, we decided to collect ancestry to avoid reifying a specific race or ethnicity taxonomy that might not be consistently interpreted by our global participants.

K Benchmarking Analysis

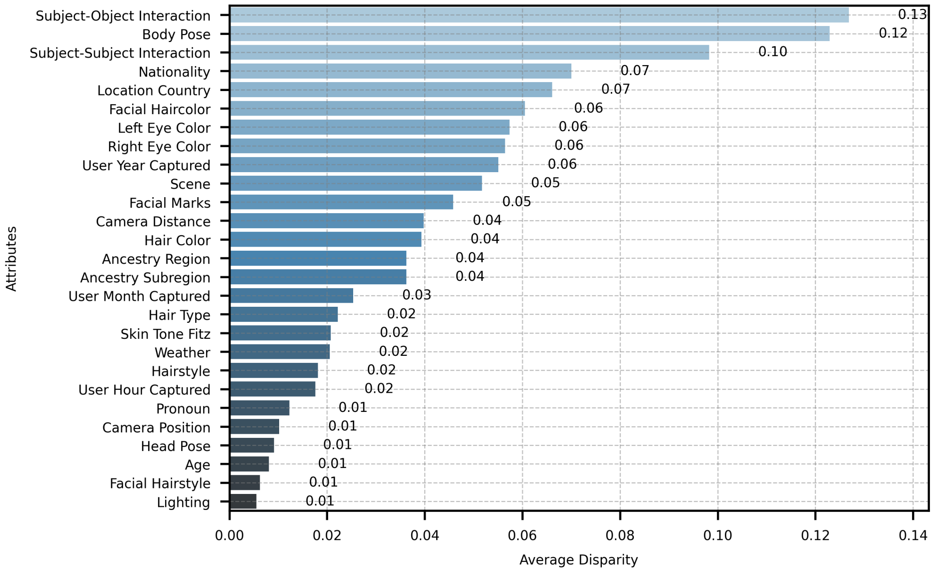

Supplementary Figure 19: **Average min-max group disparity across all tasks and models.** This figure illustrates the average min-max group disparity across various attributes for all downstream tasks and models. Disparity is quantified as $1 - \frac{\textsc{Med}(\text{worst group})}{\textsc{Med}(\text{best group})}$, where $\textsc{Med}(g)$ represents the median performance score of group g. Attributes include factors such as interactions with other subjects or objects, body pose, lighting, head pose, and facial hairstyle, among others. This visualization provides a comparative overview of disparity across attributes, with higher values indicating greater performance differences between groups.

In Figure 19, we calculate the average disparity for each attribute across all tasks and models to better understand its impact on performance. The attributes *subject-object interaction*, *body pose* and *subject-subject interaction* have the highest disparity, indicating that particular body poses depending on the subjects' actions outperform others significantly. Other attributes, such as *eye color*, *facial haircolor*, scene, and *facial marks* exhibit moderate disparities, while attributes like *lighting*, *head pose*, and *facial hairstyle* show very small disparities. Notably, both *location country* and *nationality* consistently display high disparities, highlighting the need for further investigation (cf. Figure 20 and Table 30).

We add another level of detail by calculating, for each task, the average disparity of each attribute across all models within that task. As shown in Figure 20, *body pose* and *subject-subject interaction* consistently exhibit significant disparities across most of the tasks involving the full-body of the subjects. Overall, face detection, person parsing, and person detection show the most and highest disparities, with face detection being the most affected one across all attributes, while keypoint estimation, face super-resolution, and face reconstruction display the smallest disparities.

Notably, disparities in *body pose*, *subject-subject interaction*, and *subject-object interaction* emerge in tasks like person detection, person parsing, and face detection, suggesting that complex poses or interactions (e.g., swimming) increase the likelihood of errors. Similarly, attributes like *facial haircolor*, *facial marks*, and *hair type* show disparities in face parsing, indicating their contribution to model prediction errors. Additionally, disparities in *scene* are evident in person parsing and face detection, while *camera distance* in face detection is among the most disparate attributes, suggesting a strong correlation with performance errors.

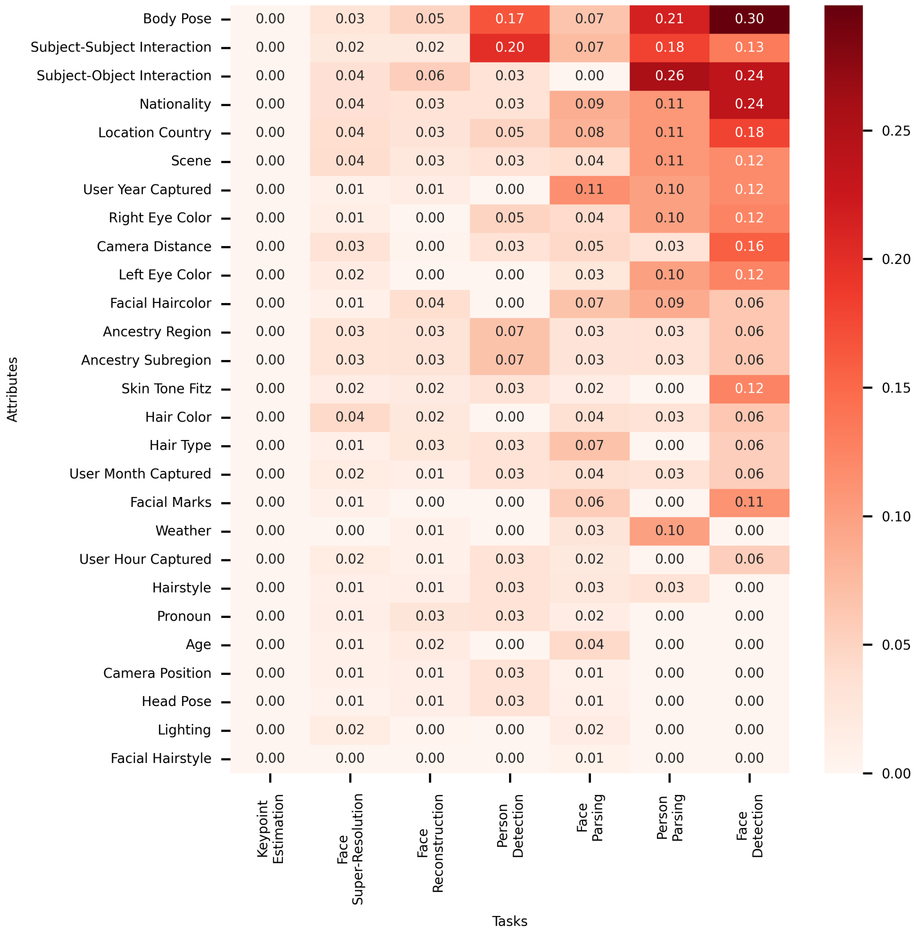

Supplementary Figure 20: **Average min-max group disparity of each attribute across all models within a task.** This figure illustrates the average min-max group disparity across all models within a task. Disparity is quantified as $1 - \frac{\textsc{Med}(\text{worst group})}{\textsc{Med}(\text{best group})}$, where $\textsc{Med}(g)$ represents the median performance score of group g. Attributes include factors such as interactions with other subjects or objects, body pose, lighting, head pose, and facial hairstyle, among others. This visualization provides a comparative overview of disparity across attributes, with higher values indicating greater performance differences between groups.

In Table 30, we present a comprehensive analysis of disparities across various tasks, models, and attributes, highlighting both the best and worst-performing groups. The table shows the top 50 rows (out of 336), ranked by disparity in descending order. Notably, the attributes *body pose* and *subject-subject interaction* appear 7 times each (14% of the total top-50 table), emphasizing their substantial disparity. *Subject-object interaction* (5), *location country* (4), *nationality* (4) and *scene* (4), are the next attributes that appear more frequently, while the rest of the attributes appear fewer times. Among tasks, face detection exhibits the most disparities (26 instances), followed by person parsing (15) and person detection (8). The least robust models are MTCNN (13 instances), RetinaFace (13), and Cascade Mask R-CNN (7).

Regarding *subject-subject interaction*, the best-performing group is often "Giving/serving or taking/receiving" (two-subject images) and "Not applicable" (single-subject images), while the worst-performing group typically involves "hugging/embracing" (two-subject images). Note, however, that the best-performing actions (i.e., "Giving/serving or taking/receiving") typically represent cases where the subjects are not

Task	Model	Attribute	Disparity	Worst group	Best group
Face Detection	MTCNN	Body Pose	0.38	Lying down/sleeping	Swimming
Person Parsing	Cascade Mask R-CNN	Subject-Object Interaction	0.33	Playing musical instrument	Brushing teeth
Person Parsing	Mask R-CNN	Subject-Object Interaction	0.33	Playing musical instrument	Brushing teeth
Person Detection	Faster R-CNN	Subject-Subject Interaction	0.30	Hugging/embracing	Not applicable
Face Detection	RetinaFace	Camera Distance	0.25	CD V	CD II
Face Detection	MTCNN	Subject-Object Interaction	0.25	Writing/drawing or painting	Brushing teeth
Face Detection	MTCNN	Location Country	0.25	Kenya	Italy
Face Detection	MTCNN	Nationality	0.25	Kenyan	Filipino
Face Detection	RetinaFace	Body Pose	0.22	Lying down/sleeping	Jumping/leaping
Person Parsing	Cascade Mask R-CNN	Body Pose	0.22	Swimming	Standing
Person Parsing	Cascade Mask R-CNN	Subject-Subject Interaction	0.22	Hugging/embracing	Giving/serving or taking/receiving
Person Parsing	Mask R-CNN	Subject-Subject Interaction	0.22	Hugging/embracing	Giving/serving or taking/receiving
Face Detection	RetinaFace	Subject-Object Interaction	0.22	Writing/drawing or painting	Entering or exiting
Person Parsing	Mask R-CNN	Body Pose	0.22	Swimming	Standing
Face Detection	RetinaFace	Nationality	0.22	English	Angolan
Person Parsing	Mask2Former	Body Pose	0.20	Swimming	Standing
Person Detection	Faster R-CNN	Body Pose	0.20	Swimming	Standing
Person Detection	Deformable DETR	Subject-Subject Interaction	0.20	Hugging/embracing	Giving/serving or taking/receiving
Person Detection	Deformable DETR	Body Pose	0.20	Swimming	Standing
Face Detection	MTCNN	Subject-Subject Interaction	0.14	Watching/looking	Giving/serving or taking/receiving
Face Detection	MTCNN	Hair Color	0.12	Dark brown/black	Light brown to medium brown
Face Detection	MTCNN	Ancestry Subregion	0.12	Eastern Africa	Central Asia
Face Detection	MTCNN	Ancestry Region	0.12	Africa	Asia
Face Detection	RetinaFace	Subject-Subject Interaction	0.12	Kissing	Giving/serving or taking/receiving
Face Detection	MTCNN	Right Eye Color	0.12	Brown	Green
Face Detection	RetinaFace	Facial Haircolor	0.12	Gray	Dark brown/black
Face Detection	MTCNN	Left Eye Color	0.12	Brown	Green
Face Detection	MTCNN	User Year Captured	0.12	2023	2019
Face Detection	MTCNN	Scene	0.12	Indoor: Home or hotel	Outdoor: Water, ice, snow
Face Detection	MTCNN	Skin Tone Fitz	0.12	Type I	Type II
Face Parsing	DML CSR	User Year Captured	0.11	2015	2022
Person Parsing	Cascade Mask R-CNN	Scene	0.11	Indoor: Transportation	Outdoor: Mountains, hills, desert, sky
Person Parsing	Mask R-CNN	Scene	0.11	Outdoor: Water, ice, snow	Outdoor: Mountains, hills, desert, sky
Person Parsing	Mask R-CNN	Nationality	0.11	Moroccan	Angolan
Person Parsing	Mask R-CNN	Location Country	0.11	Morocco	Angola
Face Detection	RetinaFace	Scene	0.11	Indoor: Workplace	Outdoor: Water, ice, snow
Person Parsing	Cascade Mask R-CNN	Facial Haircolor	0.11	Not listed	Dark brown/black
Face Detection	RetinaFace	User Year Captured	0.11	2023	2022
Face Detection	RetinaFace	User Month Captured	0.11	October	February
Person Parsing	Cascade Mask R-CNN	Location Country	0.11	Morocco	Angola
Face Detection	RetinaFace	Facial Marks	0.11	Scars	Make-up
Face Detection	RetinaFace	Hair Type	0.11	None	Curly
Face Detection	RetinaFace	Location Country	0.11	India	Angola
Face Detection	RetinaFace	User Hour Captured	0.11	06:00-11:59	00:00-05:59
Person Parsing	Cascade Mask R-CNN	Nationality	0.11	Moroccan	Angolan
Person Detection	DETR	Subject-Subject Interaction	0.10	Hugging/embracing	Giving/serving or taking/receiving
Person Parsing	Mask2Former	User Year Captured	0.10	2018	2023
Person Detection	Faster R-CNN	Subject-Object Interaction	0.10	Driving	Brushing teeth
Person Detection	Faster R-CNN	Right Eye Color	0.10	Blue	Brown
Person Detection	Faster R-CNN	Skin Tone Fitz	0.10	Type II	Type V

Supplementary Table 30: **Top-50 cases with the highest min-max group disparities across tasks and models.** This figure highlights the 50 cases with the highest min-max group disparity, selected from a total of 336 cases. Disparity quantified as $1 - \frac{\textsc{Med}(\text{worst group})}{\textsc{Med}(\text{best group})}$, where $\textsc{Med}(g)$ represents the median performance score of group g. The table includes details such as the task and model under evaluation, the attribute analyzed, the computed disparity value, the group with the worst median performance, and the group with the best median performance. This visualization provides an overview of performance disparities across different tasks and attributes.

very close to each other (0.03 median bounding box intersection between the two subjects), hence can be considered as “standalone” subjects in the image. On the other hand, for 2-subject actions that are present in the worst performing groups, such as “Hugging/embracing”, the subjects are (on median) 17 times closer to each other (0.51 median bounding box intersection), suggesting that detection models have a much harder time to separate between the two subjects.

On the other hand, for *body pose*, we observe that images of subjects with very simple poses (e.g., standing) consistently belong to the best performing group, while more

complicated poses (e.g., swimming) consistently belong to worst performing images. We investigated further the image characteristics between best and worst performing cases, and we found that images with "standing" poses, the best-performing group, have subjects with more visible keypoints (median 26) compared to "swimming" (18). In addition, images with "standing" poses are generally closer to the camera, with bounding boxes occupying a larger image area than images with "swimming" poses. Additionally, images with "standing" poses feature simpler segmentation masks (lower mask concavity) than images with "swimming" poses, and greater pairwise keypoint distances (cosine distance) than images with "swimming" poses. These attributes likely make images with standing poses "easier" for models to process, explaining their higher performance.

For *subject-object interaction*, subjects with "brushing teeth" actions consistently perform best, while those with "Playing musical instrument" or "Writing/drawing or painting" perform worst. For *scene*, images captured at "Outdoor: Mountains, hills, desert, sky" and "Outdoor: Water, ice, snow" typically perform better than those captured in indoor environments (i.e., "Indoor: Home or hotel", "Indoor: Transportation" and "Indoor: Workplace"). Such disparities may reflect insufficient training data (i.e., for subjects that play musical instruments) or the challenge posed to the models by the greater complexity and diversity of indoor environments, both of which could challenge model generalization.

Finally, an analysis of *location country* and *nationality* suggests that performance disparities are primarily influenced by specific image characteristics. Images associated with Angola—whether by location or subject nationality—exhibit the highest performance, which correlates with a greater number of visible keypoints (median of 30, compared to 25 for Morocco). Additionally, these images also tend to be closer to the camera, with subjects occupying a larger portion of the frame and positioned more centrally. Furthermore, Angola-related images display simpler segmentation masks (indicated by lower mask concavity) and greater pairwise keypoint distances (based on cosine similarity). These factors likely contribute to the images being "easier" for models to interpret, thereby resulting in improved performance relative to Morocco-related images. Overall, these findings suggest that country-specific performance rankings may be driven more by dataset-specific correlations rather than by inherent or systemic model biases.

As shown in Figure 21, we also examined average disparities for sensitive intersectional groups defined by pronoun, age, ancestry, and skin tone across all tasks and models. We observed that the intersection of *age* x *ancestry subregion* x *skin tone* yields the highest average disparities, while the simplest intersections (e.g., *pronoun* x *skin tone*) exhibit the lowest disparity. Notably, despite the fact that skin tone is often used as a proxy for ancestry/race/ethnicity in fairness evaluations [229], we find that intersections featuring both skin tone and ancestry have much greater disparities than those with only one of these attributes.

In Table 31, we provide a detailed breakdown of disparities for each task, model, and sensitive intersectional group, focusing on the best and worst-performing groups. The table presents the top 50 rows (out of 278), ranked by disparity in descending order. Our findings indicate that face detection, person parsing, and face parsing show the largest disparities compared to other full-body or face-specific tasks (i.e., face reconstruction or face super-resolution). Intersectional groups combining multiple sensitive attributes – including pronoun, age, ancestry, and skin tone – experience the most significant disparities. In particular, the intersection of skin tone with ancestry plays a crucial role in driving performance variability, and disparities increase significantly when this intersection is included alongside other attributes. Simple intersectional groups like pronoun and age typically have lower disparities.

The analysis highlights significant performance disparities across demographic groups in tasks such as face detection, person detection/parsing, and face parsing. Younger individuals (i.e., [18, 29]) frequently appear in the best-performing groups, a trend

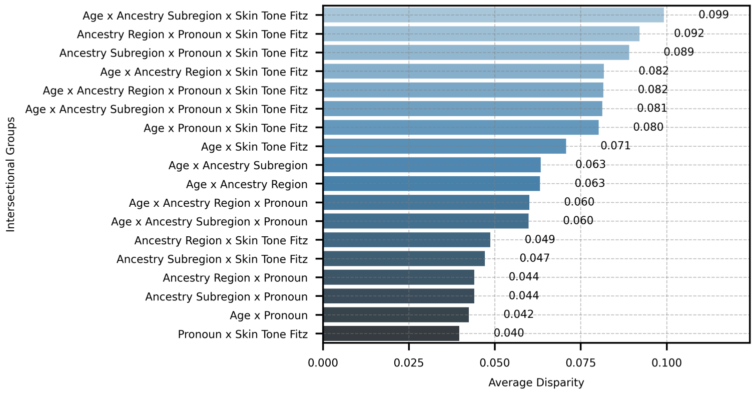

Supplementary Figure 21: **Average min-max group disparity for sensitive intersectional groups across all tasks and models.** This figure illustrates the average min-max group disparity across various sensitive intersectional group definitions for all downstream tasks and models. Disparity is quantified as $1 - \frac{\textsc{Med}(\text{worst group})}{\textsc{Med}(\text{best group})}$, where $\textsc{Med}(g)$ represents the median performance score of group g. The y-axis lists intersectional group definitions combining attributes such as age, pronoun, ancestry region, ancestry subregion, and skin tone (Fitzpatrick scale). The x-axis indicates the average disparity, with higher values reflecting larger performance differences between groups.

that is consistent across regional and skin tone combinations, suggesting an over-representation of younger persons in the training data.

Conversely, older age brackets (i.e., [50, 59], [60+]) more frequently appear as the worst-performing groups. Interestingly, such groups sometimes are also associated with darker skin tones (i.e., “He/him/his x [60, +] x Type V”, “He/him/his x [60, +] x Type IV”) or typically underrepresented regions (i.e., “‘He/him/his x [50, 59] x Type VI x Africa”, “He/him/his x [60, +] x Type VI x Africa”, “He/him/his x [60, +] x Type IV x Asia”), underscoring the limited representation of older demographics especially of particular skin tones and ancestries.

Additionally, masculine pronouns are typically associated with better performance, particularly when combined with lighter skin tones (Type II–Type III) and regions like Asia. Masculine pronouns may also appear in worst-performing groups, but typically for older age groups ([50, 59], [60, +]), or darker skin tones (Type IV–Type VI) and in regions like Africa and Asia.

On the other hand, feminine pronouns are less represented overall. They may appear as best-performing for regions like Central Asia and for lighter skin tones (Type I–Type II), while when paired with darker skin tones (Type IV, V) or regions like Europe or Caribbean, they may appear in worst-performing groups (i.e., “She/her/hers x Type V x Europe”, “She/her/hers x Type IV x Caribbean”).

Ancestry and skin tone biases are also apparent. For instance, Asia and Central Asia dominate the best-performing groups, while they may also appear in the worst-performing groups alongside African, European and Caribbean ancestries. At the same time, groups of light skin tones (Type I and II) appear more frequently (11 and 18 times respectively) as best performing groups, although darker skin tones (Type VI) may appear as best performing too (5 times). On the other hand, groups of dark skin tones (Type IV, V and VI) dominate the worst-performing groups (11, 6, and 9 times respectively). However, when ancestry and skin tone are observed in conjunction, we can see that Asia and Central Asia have a preference for Type I and II skin tones, while Asian individuals of darker skin tone (Type IV–VI) often rank among the worst-performing. Similarly, groups of African ancestry usually perform worse when

Task	Model	Intersectional Group	Disparity	Worst group	Best group
Face Detection	MTCNN	A x P x ST Fitz	0.38	He/him/his x [60, +] x Type V	He/him/his x [18, 29] x Type I
Face Detection	RetinaFace	AR x P x ST Fitz	0.33	He/him/his x Type II x Africa	She/her/hers x Type I x Asia
Face Detection	RetinaFace	AS x P x ST Fitz	0.33	He/him/his x Type II x Eastern Africa	She/her/hers x Type I x Central Asia
Face Detection	MTCNN	A x AS x P	0.25	He/him/his x [40, 49] x Eastern Africa	He/him/his x [18, 29] x Central Asia
Face Detection	MTCNN	A x AS x ST Fitz	0.25	[50, 59] x Type VI x Eastern Africa	[18, 29] x Type II x Central Asia
Face Detection	MTCNN	A x AS	0.25	[50, 59] x Eastern Africa	[18, 29] x Central Asia
Face Detection	MTCNN	A x AR	0.25	[50, 59] x Africa	[18, 29] x Asia
Face Detection	MTCNN	A x AS x P x ST Fitz	0.25	He/him/his x [40, 49] x Type VI x Eastern Africa	He/him/his x [18, 29] x Type II x Central Asia
Face Detection	MTCNN	A x AR x P x ST Fitz	0.25	He/him/his x [40, 49] x Type VI x Africa	He/him/his x [18, 29] x Type II x Asia
Face Detection	MTCNN	AR x P x ST Fitz	0.25	He/him/his x Type IV x Europe	She/her/hers x Type II x Africa
Face Detection	MTCNN	A x ST Fitz	0.25	[50, 59] x Type VI	[18, 29] x Type II
Face Detection	MTCNN	A x AR x ST Fitz	0.25	[40, 49] x Type V x Africa	[18, 29] x Type II x Asia
Face Detection	MTCNN	A x AR x P	0.25	He/him/his x [40, 49] x Africa	He/him/his x [18, 29] x Asia
Face Detection	MTCNN	AS x P x ST Fitz	0.25	He/him/his x Type IV x Eastern Europe	She/her/hers x Type II x Central Asia
Face Detection	RetinaFace	A x AS x P x ST Fitz	0.22	He/him/his x [50, 59] x Type VI x Eastern Africa	She/her/hers x [18, 29] x Type II x Central Asia
Face Detection	RetinaFace	A x AR x P	0.22	He/him/his x [50, 59] x Africa	He/him/his x [18, 29] x Europe
Face Detection	RetinaFace	A x P x ST Fitz	0.22	He/him/his x [50, 59] x Type VI	He/him/his x [40, 49] x Type III
Face Detection	RetinaFace	A x ST Fitz	0.22	[60, +] x Type V	[18, 29] x Type II
Face Detection	RetinaFace	A x AR x P x ST Fitz	0.22	He/him/his x [50, 59] x Type VI x Africa	He/him/his x [40, 49] x Type III x Asia
Face Detection	RetinaFace	A x AS x P	0.22	He/him/his x [50, 59] x Eastern Africa	He/him/his x [18, 29] x Eastern Europe
Face Detection	RetinaFace	A x P	0.22	He/him/his x [60, +]	She/her/hers x [40, 49]
Face Parsing	DML CSR	A x AR x P x ST Fitz	0.16	He/him/his x [60, +] x Type IV x Asia	He/him/his x [18, 29] x Type II x Asia
Face Parsing	DML CSR	A x AS x P x ST Fitz	0.16	He/him/his x [60, +] x Type IV x Central Asia	He/him/his x [18, 29] x Type II x Central Asia
Face Parsing	DML CSR	A x P x ST Fitz	0.15	He/him/his x [60, +] x Type IV	He/him/his x [18, 29] x Type I
Face Parsing	DML CSR	AS x P x ST Fitz	0.15	She/her/hers x Type V x Eastern Europe	He/him/his x Type II x Central Asia
Face Parsing	DML CSR	AR x P x ST Fitz	0.15	She/her/hers x Type V x Europe	He/him/his x Type II x Asia
Face Parsing	DML CSR	A x AR x ST Fitz	0.15	[60, +] x Type IV x Asia	[60, +] x Type I x Asia
Face Parsing	DML CSR	A x AS x ST Fitz	0.15	[60, +] x Type IV x Central Asia	[60, +] x Type I x Central Asia
Face Parsing	DML CSR	A x ST Fitz	0.14	[60, +] x Type IV	[60, +] x Type I
Face Detection	MTCNN	AS x P	0.12	He/him/his x Caribbean	She/her/hers x Central Asia
Face Detection	MTCNN	P x ST Fitz	0.12	He/him/his x Type I	She/her/hers x Type II
Face Detection	MTCNN	AS x ST Fitz	0.12	Type IV x Eastern Africa	Type II x Central Asia
Face Detection	MTCNN	AR x ST Fitz	0.12	Type I x Africa	Type II x Asia
Face Detection	MTCNN	AR x P	0.12	He/him/his x Africa	She/her/hers x Asia
Face Detection	MTCNN	A x P	0.12	He/him/his x [18, 29]	She/her/hers x [40, 49]
Face Parsing	DML CSR	AR x ST Fitz	0.12	Type VI x Asia	Type I x Asia
Face Parsing	DML CSR	AS x ST Fitz	0.12	Type VI x Central Asia	Type I x Central Asia
Face Detection	RetinaFace	AS x P	0.11	He/him/his x Caribbean	She/her/hers x Central Asia
Person Parsing	Mask R-CNN	A x AS x ST Fitz	0.11	[30, 39] x Type I x Eastern Africa	[30, 39] x Type VI x Eastern Africa
Face Detection	RetinaFace	A x AS	0.11	[50, 59] x Eastern Africa	[40, 49] x Central Asia
Face Detection	RetinaFace	P x ST Fitz	0.11	He/him/his x Type I	She/her/hers x Type II
Face Detection	RetinaFace	AR x P	0.11	He/him/his x Africa	She/her/hers x Asia
Person Parsing	Mask R-CNN	A x AR x P x ST Fitz	0.11	She/her/hers x [30, 39] x Type III x Africa	He/him/his x [30, 39] x Type VI x Africa
Face Detection	RetinaFace	A x AR	0.11	[40, 49] x Africa	[40, 49] x Asia
Face Detection	RetinaFace	AR x ST Fitz	0.11	Type I x Africa	Type I x Asia
Face Detection	RetinaFace	A x AR x ST Fitz	0.11	[60, +] x Type IV x Asia	[18, 29] x Type II x Asia
Face Detection	RetinaFace	A x AS x ST Fitz	0.11	[60, +] x Type V x Central Asia	[60, +] x Type I x Central Asia
Person Parsing	Cascade Mask R-CNN	AS x P x ST Fitz	0.11	She/her/hers x Type IV x Caribbean	He/him/his x Type VI x Eastern Africa
Person Parsing	Cascade Mask R-CNN	A x AS x ST Fitz	0.11	[30, 39] x Type I x Eastern Africa	[30, 39] x Type VI x Eastern Africa
Person Parsing	Mask R-CNN	A x AR x ST Fitz	0.11	[30, 39] x Type I x Africa	[30, 39] x Type VI x Africa

Supplementary Table 31: **Top-50 cases of highest min-max intersectional group disparity across tasks and models.** This figure highlights the top-50 cases (out of 278) with the highest min-max intersectional group disparity across multiple tasks and models. Disparity is quantified as $1 - \frac{\textsc{Med}(\text{worst group})}{\textsc{Med}(\text{best group})}$, where $\textsc{Med}(g)$ represents the median performance score of group g. The table includes the task and model under evaluation, the intersectional attributes analyzed, the disparity value, the group with the worst median performance, and the group with the best median performance. Attribute abbreviations: **P** (Pronoun), **A** (Age), **AR** (Ancestry Region), **AS** (Ancestry Subregion), and **ST Fitz** (Skin Tone based on the Fitzpatrick scale).

combined with light skin tones. Such results suggest that stereotypical skin tones for particular ancestries may play a role in performance.

Interestingly, after inspecting the remaining 228 rows of the table (those that are not present in the top-50) we observed that on 38 occasions some intersectional groups—-such as "He/him/his x Africa" (13 times), "He/him/his x [18, 29]" (11 times), "Type I x Africa" (10 times) and "She/her/hers x [18, 29] x Central Asia" (8 times)—perform better for one model or task but poorly for another, indicating variability tied to specific tasks and models. This pattern highlights the importance of testing for intersectional biases on a case-by-case basis, as performance disparities can vary significantly depending on the specific model-task combination. It underscores the need for cautious interpretation of bias trends, as they may not always align with a priori assumptions.

Overall, the observed disparities likely arise from a combination of systemic biases, such as under-representation in training data, and task-specific or model-specific factors interacting with sensitive attributes. Consistent trends, such as higher performance for younger individuals from Asian regions, with lighter skin tones and masculine pronouns, point to systematic biases. Conversely, task- and model-dependent variability, such as better performance for some intersectional groups in one task/-model but not another, highlights the influence of confounding factors in the data. Since our analysis is purely observational, we cannot definitively attribute every result to either cause. Yet, the richness of our dataset, with its extensive annotations, allows us to disentangle certain factors to some extent and identify patterns that are more likely due to systematic issues rather than coincidences (and vice versa).

References

[167] United Nations Department of Economic and Social Affairs, Statistics Division: Standard country or area codes for statistical use (2024)

[168] GOV.UK list of nationalities. https://www.gov.uk/government/publications/nationalities/list-of-nationalities Accessed November 1, 2022

[169] GOV.UK list of countries and territories. https://www.gov.uk/government/publications/nationalities/list-of-countries Accessed November 1, 2022

[170] COCO Keypoints Evaluation. https://cocodataset.org/#keypoints-eval (2016)

[171] Dice, L.R.: Measures of the amount of ecologic association between species. Ecology **26**(3), 297–302 (1945)

[172] Sorensen, T.: A method of establishing groups of equal amplitude in plant sociology based on similarity of species content and its application to analyses of the vegetation on danish commons. Biologiske skrifter **5**, 1–34 (1948)

[173] Jaccard, P.: The distribution of the flora in the alpine zone. 1. New phytologist **11**(2), 37–50 (1912)

[174] Jin, S., Xu, L., Xu, J., Wang, C., Liu, W., Qian, C., Ouyang, W., Luo, P.: Whole-body human pose estimation in the wild. In: European Conference on Computer Vision (ECCV) (2020)

[175] Xiao, B., Wu, H., Wei, Y.: Simple baselines for human pose estimation and tracking. In: European Conference on Computer Vision (ECCV) (2018)

[176] Sun, K., Xiao, B., Liu, D., Wang, J.: Deep high-resolution representation learning for human pose estimation. In: Computer Vision and Pattern Recognition (CVPR) (2019)

[177] Xu, Y., Zhang, J., Zhang, Q., Tao, D.: Vitpose: Simple vision transformer baselines for human pose estimation. arXiv preprint arXiv:2204.12484 (2022)

[178] Lin, T.-Y., Maire, M., Belongie, S., Hays, J., Perona, P., Ramanan, D., Dollár, P., Zitnick, C.L.: Microsoft coco: Common objects in context. In: European Conference on Computer Vision (ECCV), pp. 740–755 (2014). Springer

[179] Cheng, B., Misra, I., Schwing, A.G., Kirillov, A., Girdhar, R.: Masked-attention mask transformer for universal image segmentation. In: Computer Vision and Pattern Recognition (CVPR) (2022)

[180] Cai, Z., Vasconcelos, N.: Cascade r-cnn: high quality object detection and instance segmentation. IEEE transactions on pattern analysis and machine intelligence **43**(5), 1483–1498 (2019)

[181] He, K., Gkioxari, G., Dollár, P., Girshick, R.: Mask r-cnn. In: International Conference on Computer Vision (ICCV), pp. 2961–2969 (2017)

[182] Gustafson, L., Rolland, C., Ravi, N., Duval, Q., Adcock, A., Fu, C.-Y., Hall, M., Ross, C.: Facet: Fairness in computer vision evaluation benchmark. In: International Conference on Computer Vision (ICCV), pp. 20370–20382 (2023)

[183] Carion, N., Massa, F., Synnaeve, G., Usunier, N., Kirillov, A., Zagoruyko, S.: End-to-end object detection with transformers. In: European Conference on Computer Vision (ECCV) (2020). Springer

[184] Ren, S., He, K., Girshick, R., Sun, J.: Faster r-cnn: Towards real-time object detection with region proposal networks. Advances in neural information processing systems **28** (2015)

[185] Zhu, X., Su, W., Lu, L., Li, B., Wang, X., Dai, J.: Deformable detr: Deformable transformers for end-to-end object detection. arXiv preprint arXiv:2010.04159 (2020)

[186] Chen, Z., Yang, C., Li, Q., Zhao, F., Zha, Z.-J., Wu, F.: Disentangle your dense object detector. In: Proceedings of the 29th ACM International Conference on Multimedia, pp. 4939–4948 (2021)

[187] Schumann, C., Ricco, S., Prabhu, U., Ferrari, V., Pantofaru, C.R.: A step toward more inclusive people annotations for fairness. In: Proceedings of the AAAI/ACM Conference on AI, Ethics, and Society (AIES), pp. 916–925 (2021)

[188] Xiang, J., Zhu, G.: Joint face detection and facial expression recognition with mtcnn. In: 2017 4th International Conference on Information Science and Control Engineering (ICISCE), pp. 424–427 (2017). IEEE

[189] Cao, Q., Shen, L., Xie, W., Parkhi, O.M., Zisserman, A.: VGGFace2: A dataset for recognising faces across pose and age. In: International Conference on Automatic Face and Gesture Recognition (2018)

[190] Deng, J., Guo, J., Ververas, E., Kotsia, I., Zafeiriou, S.: Retinaface: Single-shot multi-level face localisation in the wild. In: Computer Vision and Pattern Recognition (CVPR), pp. 5203–5212 (2020)

[191] Yang, S., Luo, P., Loy, C.-C., Tang, X.: Wider face: A face detection benchmark. In: CVPR (2016)

[192] Contributors, F.P.: Face recognition using PyTorch. https://github.com/timesler/facenet-pytorch (2019)

[193] Contributors, F.: FaceXLib. https://github.com/xinntao/facexlib (2021)

[194] Lee, C.-H., Liu, Z., Wu, L., Luo, P.: Maskgan: Towards diverse and interactive facial image manipulation. In: Computer Vision and Pattern Recognition (CVPR) (2020)

[195] Zheng, Q., Deng, J., Zhu, Z., Li, Y., Zafeiriou, S.: Decoupled multi-task learning with cyclical self-regulation for face parsing. In: Computer Vision and Pattern Recognition (CVPR), pp. 4156–4165 (2022)

[196] Rothe, R., Timofte, R., Gool, L.V.: Dex: Deep expectation of apparent age from a single image. In: IEEE International Conference on Computer Vision Workshops (ICCVW) (2015)

[197] Schroff, F., Kalenichenko, D., Philbin, J.: Facenet: A unified embedding for face recognition and clustering. In: Computer Vision and Pattern Recognition, pp. 815–823 (2015)

[198] Deng, J., Guo, J., Xue, N., Zafeiriou, S.: Arcface: Additive angular margin loss for deep face recognition. In: Computer Vision and Pattern Recognition (CVPR), pp. 4690–4699 (2019)

[199] Huang, Y., Wang, Y., Tai, Y., Liu, X., Shen, P., Li, S., Li, J., Huang, F.: Curricularface: adaptive curriculum learning loss for deep face recognition. In: Computer Vision and Pattern Recognition (CVPR), pp. 5901–5910 (2020)

[200] Guo, Y., Zhang, L., Hu, Y., He, X., Gao, J.: Ms-celeb-1m: A dataset and benchmark for large-scale face recognition. In: European Conference on Computer Vision (ECCV) (2016). Springer

[201] Wang, Q., Zhang, P., Xiong, H., Zhao, J.: Face.evoLVe: A high-performance face recognition library. arXiv preprint arXiv:2107.08621 (2021)

[202] Huang, G.B., Mattar, M., Berg, T., Learned-Miller, E.: Labeled faces in the wild: A database forstudying face recognition in unconstrained environments. In: Workshop on Faces in'Real-Life'Images: Detection, Alignment, and Recognition (2008)

[203] Alaluf, Y., Patashnik, O., Cohen-Or, D.: Restyle: A residual-based stylegan encoder via iterative refinement. In: International Conference on Computer Vision (ICCV), pp. 6711–6720 (2021)

[204] Tov, O., Alaluf, Y., Nitzan, Y., Patashnik, O., Cohen-Or, D.: Designing an encoder for stylegan image manipulation. ACM Transactions on Graphics (TOG) **40**(4), 1–14 (2021)

[205] Richardson, E., Alaluf, Y., Patashnik, O., Nitzan, Y., Azar, Y., Shapiro, S., Cohen-Or, D.: Encoding in style: a stylegan encoder for image-to-image translation. In: Computer Vision and Pattern Recognition (CVPR) (2021)

[206] Karras, T., Laine, S., Aila, T.: A style-based generator architecture for generative adversarial networks. In: Computer Vision and Pattern Recognition (CVPR) (2019)

[207] Zhang, R., Isola, P., Efros, A.A., Shechtman, E., Wang, O.: The unreasonable effectiveness of deep features as a perceptual metric. In: Computer Vision and Pattern Recognition (CVPR) (2018)

[208] Simonyan, K., Zisserman, A.: Very deep convolutional networks for large-scale image recognition. arXiv preprint arXiv:1409.1556 (2014)

[209] Wang, X., Li, Y., Zhang, H., Shan, Y.: Towards real-world blind face restoration with generative facial prior. In: Computer Vision and Pattern Recognition (CVPR) (2021)

[210] Yang, T., Ren, P., Xie, X., Zhang, L.: Gan prior embedded network for blind face restoration in the wild. In: Computer Vision and Pattern Recognition (CVPR) (2021)

[211] Thornton, S., Tractenberg, R.E.: Ethical Considerations for Data Involving Human Gender and Sex Variables. https://doi.org/10.48550/arXiv.2401.01966 . http://arxiv.org/abs/2401.01966 Accessed 2025-05-28

[212] Schwabish, J., Harvey, D., Langness, M., Pancini, V., Rogin, A., Velasco, G.: Do No Harm Guide: Collecting, Analyzing, and Reporting Gender and Sexual Orientation Data. https://www.urban.org/research/publication/do-no-harm-guide-collecting-analyzing-and-reporting-gender-and-sexual Accessed 2025-05-28

[213] Tomasev, N., McKee, K.R., Kay, J., Mohamed, S.: Fairness for Unobserved Characteristics: Insights from Technological Impacts on Queer Communities. In: Proceedings of the 2021 AAAI/ACM Conference on AI, Ethics, and Society. AIES '21, pp. 254–265. Association for Computing Machinery. https://doi.org/10.1145/3461702.3462540 . https://dl.acm.org/doi/10.1145/3461702.3462540 Accessed 2025-05-28

[214] Scheuerman, M.K., Paul, J.M., Brubaker, J.R.: How computers see gender: An evaluation of gender classification in commercial facial analysis services. Proceedings of the ACM on Human-Computer Interaction **3**(CSCW), 1–33 (2019)

[215] Vassallo, M., Ihnat, J.M., Flores-Pérez, P., Rancu, A.L., Allam, O., Alperovich, M.: Facial Determinants of Artificial Intelligence–Perceived Gender and Age Following Facial Feminization Surgery **36**(2), 734 (March/April 2025) https://doi.org/10.1097/SCS.0000000000011135 . Accessed 2025-06-04

[216] Fein, L.A., Salgado, C.J., Alvarez, C.V., Estes, C.M.: Transitioning Transgender: Investigating the Important Aspects of the Transition: A Brief Report **29**(1), 80–88 https://doi.org/10.1080/19317611.2016.1227013 . Accessed 2025-06-04

[217] Gorton, R.N., Erickson-Schroth, L.: Hormonal and Surgical Treatment Options for Transgender Men (Female-to-Male) **40**(1), 79–97 https://doi.org/10.1016/j.psc.2016.10.005 28159147. Accessed 2025-06-04

[218] Truitt, B.: Woman says security guard at Liberty Hotel in Boston confronted her in bathroom, asked to prove gender. Accessed 2025-06-04

[219] Wiggins, C.: Cops confront lesbian in AZ Walmart bathroom over masculine look. Accessed 2025-06-04

[220] Duarte, R.: Couple Convinced Cis Woman Is Trans, Physically Blocks Her From Gym Bathroom. Accessed 2025-06-04

[221] Morning, A.: Ethnic classification in global perspective: A cross-national survey of the 2000 census round **27**(2), 239–272 https://doi.org/10.1007/s11113-007-9062-5 41217947. Accessed 2020-01-14

[222] Jaime, S., Kern, C.: Ethnic Classifications in Algorithmic Fairness: Concepts, Measures and Implications in Practice. In: Proceedings of the 2024 ACM Conference on Fairness, Accountability, And Transparency. FAccT '24, pp. 237–253. Association for Computing Machinery. https://doi.org/10.1145/3630106.3658902 . https://doi.org/10.1145/3630106.3658902 Accessed 2024-06-28

[223] Scheuerman, M.K., Wade, K., Lustig, C., Brubaker, J.R.: How we've taught algorithms to see identity: Constructing race and gender in image databases for facial analysis. Proceedings of the ACM on Human-computer Interaction **4**(CSCW1), 1–35 (2020)

[224] Roth, W.D.: The multiple dimensions of race. Ethnic and Racial Studies **39**(8), 1310–1338 (2016)

[225] Gabbert, W.: Concepts of Ethnicity **1**(1), 85–103 https://doi.org/10.1080/17486830500510034 . Accessed 2025-06-04

[226] LaBreck, A.: Race Policy in France: France's "Color-Blind" Approach to Race Relations **41**(4), 27–30 27275657. Accessed 2025-06-04

[227] Braveman, P., Parker Dominguez, T.: Abandon "Race." Focus on Racism **9** https://doi.org/10.3389/fpubh.2021.689462 . Accessed 2025-06-02

[228] Dauda, B., Molina, S.J., Allen, D.S., Fuentes, A., Ghosh, N., Mauro, M., Neale, B.M., Panofsky, A., Sohail, M., Zhang, S.R., Lewis, A.C.F.: Ancestry: How researchers use it and what they mean by it **14** https://doi.org/10.3389/fgene.2023.1044555 . Accessed 2025-06-02

[229] Thong, W., Joniak, P., Xiang, A.: Beyond skin tone: A multidimensional measure

of apparent skin color. In: Proceedings of the IEEE/CVF International Conference on Computer Vision, pp. 4903–4913 (2023)
