# Supplementary material for: Fair human-centric image dataset for ethical AI benchmarking
Source: Nature. 2025 Nov 5;648(8092):97–108. doi: 10.1038/s41586-025-09716-2 (PMC12675298; doi:10.1038/s41586-025-09716-2)
Supplement: Supplementary file 2 — Reporting Summary [file 41586_2025_9716_MOESM2_ESM.pdf]

nature portfolio

Corresponding author(s): Alice Xiang

Last updated by author(s): Oct 29, 2025

Reporting Summary

Nature Portfolio wishes to improve the reproducibility of the work that we publish. This form provides structure for consistency and transparency in reporting. For further information on Nature Portfolio policies, see our Editorial Policies and the Editorial Policy Checklist.

Statistics

For all statistical analyses, confirm that the following items are present in the figure legend, table legend, main text, or Methods section.

n/a	Confirmed	
☐	☒	The exact sample size (n) for each experimental group/condition, given as a discrete number and unit of measurement
☒	☐	A statement on whether measurements were taken from distinct samples or whether the same sample was measured repeatedly
☐	☒	The statistical test(s) used AND whether they are one- or two-sided<br>*Only common tests should be described solely by name; describe more complex techniques in the Methods section.*
☐	☒	A description of all covariates tested
☐	☒	A description of any assumptions or corrections, such as tests of normality and adjustment for multiple comparisons
☐	☒	A full description of the statistical parameters including central tendency (e.g. means) or other basic estimates (e.g. regression coefficient) AND variation (e.g. standard deviation) or associated estimates of uncertainty (e.g. confidence intervals)
☒	☐	For null hypothesis testing, the test statistic (e.g. F, t, r) with confidence intervals, effect sizes, degrees of freedom and P value noted<br>*Give P values as exact values whenever suitable.*
☒	☐	For Bayesian analysis, information on the choice of priors and Markov chain Monte Carlo settings
☒	☐	For hierarchical and complex designs, identification of the appropriate level for tests and full reporting of outcomes
☒	☐	Estimates of effect sizes (e.g. Cohen's d, Pearson's r), indicating how they were calculated

Our web collection on statistics for biologists contains articles on many of the points above.

Software and code

Policy information about availability of computer code

Data collection

The data collection process did not involve the use of commercial or open-source software for data capture, as images were submitted directly by participants using their own devices to data vendors. Annotations were performed using vendor-provided platforms.

Data analysis

Data analysis was conducted using a combination of open-source tools and custom scripts. Python (version 3.10) served as the primary programming language, with key dependencies including Pandas (2.2.1) for data manipulation, NumPy (1.26.4) for numerical computations, Scikit-learn (1.5.0) for statistical and machine learning analyses, and Torch (2.2.0 for the machine learning and deep learning framework. Image processing tasks were handled using OpenCV (4.10.0.84) and Pillow (10.2.0), while visualization was performed with Matplotlib (3.8.3) and Seaborn (0.13.2). For annotation processing, Flask-based infrastructure (2.2.2) was employed to facilitate internal manual review. The analysis pipeline was managed using Poetry (2.1.1) for dependency control, ensuring reproducibility.

For the utility evaluations, we used torchvision for pre-trained models. For bias diagnosis, we used jupyterlab (4.2.5) for analysis, the FP Growth algorithm via mlxtend (0.23.1), CLIP from OpenAI, transformers from Hugging Face, and the Mann-Whitney U test algorithm from scipy (1.13.1). For measuring dataset diversity, we used vendi-score (0.0.3). For inpainting using Stable Diffusion, we used transformers (4.39.1), diffusers (0.27.2). For logo detection, we utilized MM2021 Robust Logo Detector and the Google Vision API for logo detection.

The custom code we developed for running the fairness benchmarks across various computer vision tasks on the FHIBE dataset is publicly available at https://github.com/SonyResearch/fairness-benchmark-public.

For manuscripts utilizing custom algorithms or software that are central to the research but not yet described in published literature, software must be made available to editors and reviewers. We strongly encourage code deposition in a community repository (e.g. GitHub). See the Nature Portfolio guidelines for submitting code & software for further information.

Data

Policy information about availability of data

All manuscripts must include a data availability statement. This statement should provide the following information, where applicable:

- Accession codes, unique identifiers, or web links for publicly available datasets
- A description of any restrictions on data availability
- For clinical datasets or third party data, please ensure that the statement adheres to our policy

The FHIBE dataset is publicly available at https://fairnessbenchmark.ai.sony. At this site, users are required to register an account with a valid email address and to agree to the Terms of Use, after which access is immediately provided. Such controls ensure that data protection terms and other legal provisions are agreed to and that notices and obligations related to the handling of the dataset can be communicated.

The Terms of Use only permit FHIBE to be used for fairness/bias mitigation purposes. FHIBE cannot be used for training, with the narrow exception of training bias mitigation tools. This restriction preserves the utility of FHIBE as an evaluation set (models cannot be first trained on and then evaluated on FHIBE). It also reduces potential harms, such as the use of the data to train prediction algorithms for sensitive (e.g., gender, race, sexual orientation) or objectionable (e.g., attractiveness, criminality) attributes or the reproduction of individuals' likeness through being included in generative AI training sets.

Individuals may request the removal of their data and the dataset will be updated and re-released (to maintain size and diversity), as appropriate, in response to removal requests. Users with access to the dataset will then be notified and directed to delete portions of the dataset or to delete it in its entirety and use the updated version of the dataset, as required in our Terms of Use.

In addition to FHIBE, the following datasets were used in the study. All of these datasets were publicly available at the time the study was conducted.

COCO 2014 Validation: http://images.cocodataset.org/annotations/annotations_trainval2014.zip
COCO Whole Body: https://drive.google.com/file/d/1thErEToRbmM9uLNi1JXXfOsaS5VK2FXf, https://drive.google.com/file/d/1N6VgwKnj8DeyGXCvp1eYgNbRmw6jdfrb
Annotations for COCO Whole Body: https://docs.google.com/forms/d/e/1FAIpQLSdjLGJ2AhOKBGou_VqaWpLJUAL3ieJ2WNmEmGnuIZgCvjbx2Q/viewform

FACET: https://ai.meta.com/datasets/facet-downloads

Open Images MIAP: https://storage.googleapis.com/openimages/open_images_extended_miap/open_images_extended_miap_images_train.lst, https://storage.googleapis.com/openimages/open_images_extended_miap/open_images_extended_miap_images_val.lst, https://storage.googleapis.com/openimages/open_images_extended_miap/open_images_extended_miap_images_test.lst, https://storage.googleapis.com/openimages/open_images_extended_miap/open_images_extended_miap_boxes_train.csv, https://storage.googleapis.com/openimages/open_images_extended_miap/open_images_extended_miap_boxes_val.csv, https://storage.googleapis.com/openimages/open_images_extended_miap/open_images_extended_miap_boxes_test.csv

WiderFace: https://drive.google.com/file/d/15hGDLhsx8bLgLcIRD5DhYt5iBxnjNF1M, https://drive.google.com/file/d/1GUCogbp16PMGa39thoMMeWxp7Rp5oM8Q, https://drive.google.com/file/d/1HIfDbVEWKmsYKJZm4lchTBDLW5N7dY5T

CelebAMask-HQ: https://drive.google.com/file/d/1badu11NqxGf6qM3PTTooQDJvQbejgbTv

CCv1: https://ai.facebook.com/datasets/casual-conversations-dataset/
CCv2: https://ai.meta.com/datasets/casual-conversations-v2-downloads/

IMDB-WIKI: https://data.vision.ee.ethz.ch/cvl/rrothe/imdb-wiki/static/imdb_crop.tar, https://data.vision.ee.ethz.ch/cvl/rrothe/imdb-wiki/static/wiki_crop.tar

Research involving human participants, their data, or biological material

Policy information about studies with human participants or human data. See also policy information about sex, gender (identity/presentation), and sexual orientation and race, ethnicity and racism.

Reporting on sex and gender	Participants did not report sex or gender. They did, however, self-report their gender pronouns. We allowed for multiple selections from a predefined list (or "Prefer not to say"). No inferences were made about participants' sex or gender based on pronoun selections. Subjects consented to the release of this information as part of the public dataset. The distribution of images and subjects across pronouns can be found in Supplement F.2. Analysis results using the pronoun data can be found in the Evaluation Results section.
Reporting on race, ethnicity, or other socially relevant groupings	Race and ethnicity were not collected in this study. Participants self-reported their ancestry at a required regional level and an optional sub-regional level based on United Nations Statistics Division (UNSD) categories (see Supplement A). This was done to provide a consistent frame of reference. Participants were asked, "Where do your ancestors (e.g., great-grandparents) come from?" These responses were self-reported and were not used as proxies for race, ethnicity, or socioeconomic status. Other socially relevant groupings collected included self-reported nationality, and country of residence, and apparent and natural skin tone (using predefined RGB-based categories inspired by the Fitzpatrick scale).
Population characteristics	Collected participant characteristics included self-reported age, pronouns, nationality, country/territory of residence, ancestry (regional and sub-regional), skin tone, eye color, hair type, hair style, hair color, facial hairstyle, facial hair color, height, weight, facial marks, biologically related subject, disability/difficulty status, pregnancy status, subject-object interaction, and subject-subject interaction (See Supplement A). These attributes were self-reported by participants and provided directly through a data vendor's platform. They were not inferred from images.

	Additionally, image annotations such as time and date of capture, place of capture, weather, facial illumination, scene, and camera position were collected. In cases where an image contained two consensual image subjects, separate annotations were obtained for each subject. Head pose and camera distance were further annotated by data annotators after submission.
Recruitment	Participants were recruited through external data vendors, who were required to ensure that all image subjects provided explicit informed consent. Only individuals above the age of majority in their country of residence were eligible to participate. Vendors were instructed not to use referral programs or provide recruitment incentives beyond standard compensation. To ensure understanding of the study terms, participants had to demonstrate basic English proficiency by answering at least two out of three multiple-choice questions correctly before participation. Potential self-selection biases include the requirement for English proficiency, which may have limited participation from non-English-speaking populations.
Ethics oversight	Data collection commenced after April 23, 2023, following Institutional Review Board (IRB) approval from WCG Clinical, Inc. (study number 1352290). All participants provided informed consent, and image subjects additionally consented to their identifiable images being included in the dataset.

Note that full information on the approval of the study protocol must also be provided in the manuscript.

Field-specific reporting

Please select the one below that is the best fit for your research. If you are not sure, read the appropriate sections before making your selection.

☐ Life sciences ☒ Behavioural & social sciences ☐ Ecological, evolutionary & environmental sciences

For a reference copy of the document with all sections, see nature.com/documents/nr-reporting-summary-flat.pdf

Behavioural & social sciences study design

All studies must disclose on these points even when the disclosure is negative.

Study description	This study involves the collection of quantitative data through self-reported attributes, image submissions, and additional annotations. Participants provided structured responses via a data vendor's platform, and the research team and QA workers conducted additional annotations and quality control.
Research sample	The research sample consists of crowdsourced image subjects who voluntarily participated through data vendor platforms. Participants provided images along with demographic and physical characteristic annotations, including age, pronouns, nationality, country/territory of residence, ancestry, and other self-reported attributes. See Supplement F.2 for information on the demographic distribution. The dataset was designed to maximize diversity across multiple attributes rather than represent a specific population.
Sampling strategy	Participants were recruited through external data vendors following predefined inclusion criteria. The vendors ensured compliance with guidelines prioritizing diversity across demographic, environmental, and imaging conditions, roughly resulting in stratified sampling across these dimensions. Given that FHIBE was collected with the aim of being used to detect bias across a wide variety of tasks and models (many possible hypotheses), and FHIBE sampled from a distribution distinct from existing publicly available datasets, power analyses at the outset of the project using existing datasets were unreliable. The initial sample size was determined based on previously collected proprietary datasets and budget constraints. We have verified, however, the utility of FHIBE in our analyses showing that FHIBE is able to detect statistically significant biases for many human-centric computer vision task-model pairs.
Data collection	Images and self-reported annotations were collected via vendor platforms, with participants submitting both image data and attribute information. Vendors facilitated the collection of consent forms and copyright agreements. To avoid potentially coercive practices, we instructed vendors not to provide participants support (beyond platform tutorials and general technical support) in signing up for or submitting to the project. After data submission, additional annotations—including apparent attributes and environmental metadata—were collected through a combination of manual annotation by QA workers and automated methods. The research team conducted additional validation and quality control.
Timing	Data collection commenced after April 23, 2023, following IRB approval from WCG Clinical, Inc. (study number 1352290). The dataset consists of images and annotations collected within a defined period through vendor-managed platforms. Note, however, participants were allowed to submit historical images, i.e., images taken prior to April 23, 2023. The final delivery of images used in the initial launch of FHIBE was on June 26, 2024. More images might be collected going forward for future versions of the dataset.
Data exclusions	Overall, in order to arrive at the 10,319 images for the initial launch of FHIBE, we collected a total of 28,703 images from three data vendors. 6,868 images were removed due to noncompliance with project guidelines and quality specifications, while 5,855 images were excluded due to issues with consent or copyright forms. An additional 3,848 images were identified as potentially fraudulent and removed following a combination of automated and manual verification methods. A small number of images (~11) were excluded due to minor annotation inconsistencies (e.g., missing skin color annotations), ~27 were removed for containing offensive content, and ~2 were excluded for other reasons such as duplicate subject IDs.<br><br>These exclusions were implemented as part of predefined quality control measures conducted by vendors and the research team to ensure the integrity and ethical compliance of the dataset. More information about these exclusions can be found in Methods.
Non-participation	Participants can voluntarily choose to withdraw their data from the study at any point for any reason, without any impact on the compensation they received for their participation. So far, three participants have withdrawn their data.

Randomization	Participants were not allocated into experimental groups.

Reporting for specific materials, systems and methods

We require information from authors about some types of materials, experimental systems and methods used in many studies. Here, indicate whether each material, system or method listed is relevant to your study. If you are not sure if a list item applies to your research, read the appropriate section before selecting a response.

Materials & experimental systems

n/a	Involved in the study
☒	☐ Antibodies
☒	☐ Eukaryotic cell lines
☒	☐ Palaeontology and archaeology
☒	☐ Animals and other organisms
☒	☐ Clinical data
☒	☐ Dual use research of concern
☒	☐ Plants

Methods

n/a	Involved in the study
☒	☐ ChIP-seq
☒	☐ Flow cytometry
☒	☐ MRI-based neuroimaging

Plants

Seed stocks	N/A
Novel plant genotypes	N/A
Authentication	N/A
